# Supplementary material for: Isomeranzin activates Gnas-AMPK signaling to drive white adipose browning and curb obesity in mice
Source: EMBO Mol Med. 2025 Nov 26;18(1):55–90. doi: 10.1038/s44321-025-00335-y (PMC12808274; doi:10.1038/s44321-025-00335-y)
Supplement: Supplementary file 1 — Table EV1 [file 44321_2025_335_MOESM1_ESM.docx]

Table EV1-the other genes

| GSE164219 | | | GSE133619 | | | GSE129083 | | | GSE98132 | | |
| --- | --- | --- | --- | --- | --- | --- | --- | --- | --- | --- | --- |
| Gene | log2FC | padj | Gene | log2FC | padj | Gene | log2FC | padj | Gene | log2FC | padj |
| Slc38a4 | 2.548955 | 4.58E-45 | Sidt1 | 4.537555 | 0.036761 | Ttc25 | 3.437483 | 8.98E-17 | Map7d2 | 2.131393 | 9.33E-05 |
| Myl3 | 2.547036 | 0.00013 | Gm45745 | 4.515815 | 0.007067 | Hfe2 | 3.421492 | 0.001217 | Cox8b | 2.126675 | 1.77E-06 |
| Cox7a1 | 2.530856 | 2.03E-09 | A630023P12Rik | 4.511093 | 0.016589 | Rsph4a | 3.421315 | 0.029609 | Rad51ap2 | 2.122527 | 0.002694 |
| Slc22a2 | 2.518201 | 0.011315 | Klhl14 | 4.5054 | 0.001619 | Insig1 | 3.420672 | 7.62E-39 | Gm5523 | 2.1186 | 0.006256 |
| Slc6a19 | 2.503039 | 0.003994 | Zfp831 | 4.490097 | 0.018926 | Hrasls | 3.415852 | 0.000531 | Wnt8b | 2.112531 | 0.014292 |
| Fgg | 2.470904 | 5.37E-13 | Dusp2 | 4.484227 | 0.015597 | Acta1 | 3.415713 | 0.00263 | Rfx4 | 2.079353 | 0.006122 |
| Serpina1d | 2.468285 | 2.72E-12 | Gm14085 | 4.453765 | 0.012566 | Mir675 | 3.408848 | 4.86E-06 | Cyp2c70 | 2.079353 | 0.010209 |
| Ehhadh | 2.448594 | 0.000346 | Vpreb3 | 4.448932 | 0.003119 | 1700030F18Rik | 3.399794 | 0.01105 | Saa2 | 2.075771 | 0.024143 |
| Ces3a | 2.446056 | 0.009506 | Traj49 | 4.448711 | 0.003515 | Hapln4 | 3.399643 | 0.000808 | Cspg5 | 2.069284 | 4.93E-05 |
| Serpina1a | 2.426619 | 0.010273 | Themis | 4.443728 | 0.013676 | Popdc3 | 3.384971 | 0.00677 | Wdr38 | 2.068752 | 0.049722 |
| 2210418O10Rik | 2.393942 | 1.60E-28 | Cox7a1 | 4.437596 | 1.00E-08 | Plet1 | 3.374258 | 0.005048 | Dcx | 2.066382 | 0.02434 |
| Gchfr | 2.377497 | 0.007123 | Insm1 | 4.426252 | 0.000806 | Agpat2 | 3.368785 | 9.27E-25 | Gnao1 | 2.064871 | 2.06E-05 |
| Ak1 | 2.358162 | 1.98E-07 | A530030E21Rik | 4.426 | 0.029041 | AI317395 | 3.366504 | 2.99E-21 | Plcd4 | 2.062559 | 0.047192 |
| Spata17 | 2.341627 | 0.049948 | Bpifb3 | 4.41656 | 0.002877 | 6030408B16Rik | 3.348257 | 0.007942 | Olfr11 | 2.061864 | 5.86E-06 |
| Ankrd9 | 2.337906 | 0.002545 | 5830428M24Rik | 4.411727 | 0.015103 | Pvalb | 3.338319 | 0.005772 | Tpd52l1 | 2.058841 | 3.38E-05 |
| Slc29a4 | 2.324522 | 5.68E-11 | Trbc2 | 4.410297 | 0.010577 | Acly | 3.331748 | 9.89E-33 | Retsat | 2.056553 | 0.000118 |
| Acaa1b | 2.308787 | 0.002408 | Bcl11a | 4.39374 | 0.010831 | Ppp1r14c | 3.327543 | 2.45E-05 | Pik3c2g | 2.0529 | 0.002274 |
| Slc4a4 | 2.28333 | 1.44E-24 | Cd72 | 4.389053 | 0.026438 | Rpl3l | 3.323894 | 0.001658 | 1700061I17Rik | 2.0503 | 0.036437 |
| Ccdc18 | 2.280701 | 0.024098 | St8sia1 | 4.374322 | 0.000371 | Gm10768 | 3.292546 | 0.005585 | Tekt1 | 2.039191 | 0.01159 |
| Nrtn | 2.268608 | 2.49E-23 | Serpina1e | 4.362764 | 0.001119 | Abra | 3.291264 | 0.0006 | Gm10768 | 2.037509 | 0.014969 |
| Arg1 | 2.263081 | 2.47E-27 | Tspan10 | 4.354767 | 0.000508 | Myot | 3.290389 | 0.00843 | Fsd1 | 2.032993 | 0.000772 |
| Tenm2 | 2.255729 | 0.001175 | 1700062C10Rik | 4.349564 | 0.002156 | Kcna7 | 3.289633 | 1.91E-05 | Poln | 2.016254 | 3.22E-06 |
| Mup7 | 2.251835 | 0.001021 | Gm32468 | 4.344956 | 0.007362 | Yipf7 | 3.285164 | 0.000811 | Gm6498 | 2.015962 | 0.006214 |
| St3gal5 | 2.249543 | 0.013646 | Scml4 | 4.343798 | 0.006141 | Tmem52 | 3.278185 | 6.01E-09 | Acot12 | 2.015105 | 0.026852 |
| Pld6 | 2.241952 | 0.002012 | Otop1 | 4.339378 | 9.95E-06 | Apoa2 | 3.269836 | 0.0002 | Dnajc22 | 2.006987 | 4.53E-05 |
| Chrna2 | 2.21972 | 7.80E-05 | Arhgap27os3 | 4.317337 | 0.020966 | Hrc | 3.26407 | 0.006793 | 1700055N04Rik | 2.005478 | 3.40E-05 |
| Arhgef19 | 2.218784 | 1.26E-31 | Haao | 4.303082 | 0.029447 | Fasn | 3.253781 | 1.40E-06 | Wfdc12 | 2.005222 | 0.001095 |
| Adcy3 | 2.218334 | 0.002648 | Creg2 | 4.286329 | 0.001709 | Mir30d | 3.251357 | 0.046676 | Mup3 | 1.996256 | 6.87E-05 |
| Cyp2b10 | 2.213677 | 5.75E-17 | Il4i1 | 4.254979 | 0.021241 | Casq1 | 3.244213 | 0.002961 | Perm1 | 1.996035 | 0.002081 |
| Dbp | 2.209898 | 4.81E-12 | Kynu | 4.254086 | 0.000814 | Tnni2 | 3.234956 | 0.001844 | Olfr212 | 1.992953 | 0.000566 |
| Slc15a5 | 2.203398 | 2.02E-46 | Elovl6 | 4.233857 | 4.14E-06 | Pygm | 3.221766 | 0.000278 | Hoxa4 | 1.980593 | 0.00014 |
| Cyp2c70 | 2.194208 | 1.41E-06 | Lck | 4.226883 | 0.007851 | Myom2 | 3.218045 | 0.002318 | Chchd10 | 1.980535 | 0.000101 |
| Npnt | 2.175617 | 1.44E-15 | Nlrc3 | 4.224358 | 0.003403 | Cacng1 | 3.216996 | 0.016956 | Rpl39l | 1.977033 | 3.06E-06 |
| Tmem37 | 2.169739 | 0.01132 | Itk | 4.192342 | 0.026095 | Mab21l2 | 3.214285 | 0.000595 | Rnf207 | 1.976966 | 0.000548 |
| Pnldc1 | 2.166652 | 0.012592 | Gys2 | 4.191235 | 9.59E-06 | Sypl2 | 3.20369 | 0.000125 | Mogat2 | 1.976242 | 0.004734 |
| Pla2g2e | 2.148274 | 2.37E-23 | Gm37176 | 4.190546 | 0.008584 | Plet1os | 3.197534 | 0.000242 | Efhb | 1.973243 | 0.043915 |
| Pank1 | 2.130036 | 5.82E-13 | Lad1 | 4.1763 | 0.015506 | Chchd10 | 3.181895 | 3.75E-79 | Vipr2 | 1.972099 | 0.000215 |
| Serpinc1 | 2.111592 | 4.35E-11 | Pglyrp2 | 4.168881 | 0.006079 | Trdn | 3.17957 | 0.008142 | Gys2 | 1.971382 | 9.97E-05 |
| Pfkm | 2.099154 | 7.61E-12 | Bmp8b | 4.150719 | 0.000635 | 2010003K11Rik | 3.177711 | 3.69E-27 | Hephl1 | 1.962405 | 0.000906 |
| Tcea3 | 2.093637 | 1.36E-44 | Acly | 4.146163 | 6.96E-07 | H19 | 3.1775 | 3.12E-05 | Urah | 1.958655 | 0.019518 |
| Apoc2 | 2.083154 | 0.000377 | Fam169b | 4.118903 | 0.055498 | Kcnc4 | 3.171741 | 0.000536 | Tfr2 | 1.947845 | 0.001821 |
| Serpina1e | 2.07894 | 0.004605 | Grap2 | 4.108279 | 0.040626 | Tmprss6 | 3.149718 | 3.23E-08 | 4930500J02Rik | 1.941007 | 0.013619 |
| S100b | 2.071172 | 0.038436 | Ptgds | 4.099003 | 0.001271 | Tgm3 | 3.141297 | 7.93E-05 | Lrit1 | 1.941007 | 0.030332 |
| Tfrc | 2.069536 | 0.001088 | Art2a-ps | 4.088765 | 0.008896 | Ryr1 | 3.1193 | 0.00048 | Ucp1 | 1.919933 | 0.001288 |
| Slc25a42 | 2.054085 | 0.013514 | Lat | 4.088521 | 0.017454 | Mup17 | 3.118191 | 0.001416 | Chrdl2 | 1.915009 | 0.00603 |
| Arhgef37 | 2.046307 | 6.40E-06 | Skap1 | 4.084804 | 0.005073 | Hist1h4c | 3.104423 | 1.46E-05 | Adck3 | 1.912427 | 0.000743 |
| Apoc4 | 2.031746 | 0.005846 | Serpina3k | 4.080513 | 0.001407 | Kcnd2 | 3.102782 | 0.00361 | Pla2g2e | 1.9052 | 0.014285 |
| Hebp2 | 2.028593 | 3.17E-15 | Lta | 4.078508 | 0.019255 | Adtrp | 3.099657 | 1.08E-86 | Mir1941 | 1.90251 | 0.01511 |
| Ttc9 | 2.015319 | 2.35E-26 | E430014B02Rik | 4.057874 | 0.023049 | Jsrp1 | 3.097193 | 0.004209 | Myh7b | 1.898683 | 0.002924 |
| Atp4b | 2.010711 | 7.64E-79 | Ikzf3 | 4.037054 | 0.009489 | Sucla2 | 3.093848 | 2.89E-56 | Gchfr | 1.897913 | 0.021032 |
| Cpn2 | 2.009094 | 0.00022 | Gm26583 | 4.031742 | 0.002446 | Mylk2 | 3.090548 | 0.003164 | Rasef | 1.897523 | 0.049011 |
| Slc47a1 | 2.005962 | 0.000116 | BC025920 | 4.017999 | 0.002075 | Myo18b | 3.086242 | 0.000492 | Cfhr2 | 1.896698 | 0.003262 |
| Acot11 | 2.002255 | 2.10E-05 | Tmem150c | 4.012965 | 0.006063 | Fgf21 | 3.07642 | 0.035091 | Casq1 | 1.892165 | 0.044418 |
| Mup3 | 1.999449 | 0.00821 | Gm8670 | 4.012486 | 0.001732 | Apon | 3.068616 | 0.039544 | Myl3 | 1.886018 | 0.036739 |
| Msantd5l | 1.998419 | 2.99E-15 | Ncan | 4.005124 | 0.000257 | Aldh3b2 | 3.067961 | 1.40E-31 | Olfr20 | 1.884365 | 0.004041 |
| Letmd1 | 1.988456 | 0.018012 | Dab1 | 3.999818 | 0.001206 | Nrap | 3.035899 | 0.001719 | Sult2b1 | 1.872944 | 0.00737 |
| Mup15 | 1.979315 | 7.34E-06 | Cyp2b10 | 3.998109 | 0.001355 | Gm867 | 3.026841 | 3.65E-28 | Cacng5 | 1.872855 | 0.008151 |
| Ppargc1b | 1.973033 | 9.08E-22 | Traf3ip3 | 3.977839 | 0.011778 | Acadm | 3.024349 | 2.93E-44 | Pnpla5 | 1.870818 | 0.002752 |
| Ccno | 1.968891 | 0.000961 | Gpr83 | 3.974633 | 0.003208 | Hao1 | 3.019069 | 0.048865 | Adra2b | 1.866876 | 0.000515 |
| Slc25a20 | 1.96108 | 2.22E-14 | Cideb | 3.973116 | 0.001877 | Art1 | 3.017773 | 0.020363 | Ppp1r3b | 1.860194 | 0.009615 |
| Aspg | 1.96032 | 3.33E-07 | Mcemp1 | 3.963584 | 0.018418 | Neb | 3.00869 | 0.000371 | Acly | 1.841668 | 0.006642 |
| Shisa2 | 1.956984 | 0.000595 | Gm4759 | 3.955076 | 0.005402 | Gm15179 | 3.004509 | 0.000305 | Olfr18 | 1.84158 | 0.004371 |
| Aldh1b1 | 1.933355 | 2.67E-24 | Grb7 | 3.950012 | 0.000733 | Slc25a1 | 3.003501 | 4.79E-36 | Dhrs7 | 1.83932 | 2.88E-05 |
| Mfsd4b1 | 1.928285 | 0.01132 | E330011O21Rik | 3.944423 | 0.000306 | Klhl31 | 2.992269 | 0.005005 | Trpv3 | 1.833324 | 0.036302 |
| Plin5 | 1.922155 | 7.86E-20 | Cox8b | 3.942489 | 1.00E-08 | Jph2 | 2.981256 | 0.000284 | Slc25a20 | 1.831328 | 0.000177 |
| Mmd2 | 1.914548 | 0.015846 | Sit1 | 3.929492 | 0.008718 | Gnao1 | 2.978392 | 2.32E-20 | Gprin2 | 1.830046 | 0.023863 |
| Cidea | 1.913443 | 7.07E-31 | Gm15232 | 3.925592 | 0.004585 | Cspg5 | 2.97768 | 1.24E-13 | Saa4 | 1.825832 | 0.047048 |
| Lrrc20 | 1.904116 | 1.24E-11 | Ccl22 | 3.925101 | 0.000372 | Ppp1r3b | 2.977606 | 6.39E-17 | Ankrd9 | 1.825811 | 0.001627 |
| Acot1 | 1.90386 | 0.036394 | Serpina10 | 3.918359 | 0.010997 | Tmem79 | 2.97542 | 1.30E-34 | Kcnj11 | 1.817161 | 0.005414 |
| Me3 | 1.89924 | 0.019707 | Iglc2 | 3.907135 | 0.018532 | Krt8 | 2.959499 | 0.025054 | Ocstamp | 1.815548 | 0.016687 |
| Serpine1 | 1.88926 | 6.60E-12 | Cxcr2 | 3.900454 | 0.013479 | Tfr2 | 2.955948 | 1.69E-11 | Etfdh | 1.81358 | 3.68E-05 |
| Poln | 1.871918 | 1.46E-16 | Trat1 | 3.89561 | 0.012742 | 2310015D24Rik | 2.936618 | 0.005988 | A230009B12Rik | 1.810235 | 0.021037 |
| Pim1 | 1.864659 | 0.003565 | Gstp3 | 3.893235 | 0.001425 | Csrp3 | 2.936583 | 0.004027 | Jsrp1 | 1.810235 | 0.034396 |
| 3930402G23Rik | 1.850009 | 8.74E-13 | Gm30211 | 3.892993 | 0.007792 | Poln | 2.931289 | 5.18E-25 | Clec18a | 1.810235 | 0.029645 |
| Cited1 | 1.840979 | 4.76E-05 | Dtx1 | 3.888206 | 0.016712 | Fam151a | 2.928022 | 2.22E-25 | Sec14l4 | 1.807764 | 0.000146 |
| Fhdc1 | 1.83578 | 6.14E-07 | Cabcoco1 | 3.882204 | 0.000629 | Cntnap2 | 2.924547 | 0.038407 | Ak1 | 1.797884 | 0.000246 |
| Adtrp | 1.835002 | 8.51E-13 | Il21r | 3.873627 | 0.015397 | Elovl6 | 2.920263 | 5.53E-17 | Gm20594 | 1.797036 | 0.000781 |
| Lipg | 1.826877 | 4.33E-07 | Rpgrip1 | 3.847383 | 0.009543 | Hspb7 | 2.911078 | 0.003049 | S100b | 1.796508 | 0.000813 |
| Rab20 | 1.822191 | 2.46E-14 | Parvg | 3.84103 | 0.025352 | Acadl | 2.897781 | 4.67E-35 | Letmd1 | 1.795071 | 9.77E-06 |
| Acss1 | 1.819388 | 1.52E-13 | Gm47015 | 3.829145 | 0.006745 | Klhl41 | 2.894317 | 0.002729 | Alpl | 1.788813 | 8.94E-06 |
| Pla2g7 | 1.817762 | 0.001525 | Atg9b | 3.821244 | 0.000716 | Tmem233 | 2.889058 | 0.001302 | Prg4 | 1.782103 | 0.015913 |
| Mab21l2 | 1.812606 | 0.024525 | Tagap | 3.819231 | 0.010465 | Asb16 | 2.888483 | 0.004552 | Gapdh | 1.780211 | 0.000401 |
| Itih2 | 1.811502 | 1.09E-05 | Gm30054 | 3.818953 | 0.008931 | A830009L08Rik | 2.886539 | 0.026163 | Pcdh11x | 1.779198 | 0.018432 |
| Ldhb | 1.806086 | 2.99E-15 | A430078G23Rik | 3.81817 | 0.01164 | Aco2 | 2.88068 | 5.75E-27 | Aqp2 | 1.778924 | 0.008918 |
| Adprhl1 | 1.80551 | 1.61E-05 | Gm5159 | 3.818052 | 0.001094 | Bves | 2.869005 | 0.01888 | Mapk13 | 1.774082 | 0.024178 |
| Ppargc1a | 1.801321 | 8.15E-08 | Pkp3 | 3.817551 | 0.023096 | Tcap | 2.868764 | 0.012541 | Sucla2 | 1.76936 | 2.14E-05 |
| Azgp1 | 1.763782 | 9.15E-35 | Slain1 | 3.812615 | 0.007934 | Acadvl | 2.863701 | 7.38E-50 | Snora26 | 1.763636 | 0.03229 |
| Ak4 | 1.763098 | 1.18E-12 | Adcy10 | 3.803107 | 0.000219 | Slc9a2 | 2.863204 | 0.000808 | Acot10 | 1.762429 | 0.000693 |
| Slc38a3 | 1.753062 | 0.005773 | A430093F15Rik | 3.802715 | 0.001283 | Synpo2l | 2.862113 | 0.013382 | Pla2g4e | 1.761342 | 0.0379 |
| Pde1c | 1.752714 | 0.034166 | Syndig1l | 3.802109 | 0.002457 | Ociad2 | 2.853332 | 6.12E-18 | Gm4841 | 1.760615 | 0.038904 |
| Gapdh | 1.747361 | 2.26E-16 | Cpn2 | 3.789922 | 0.000298 | Klb | 2.850154 | 2.26E-21 | Acot11 | 1.759215 | 0.003137 |
| Mup11 | 1.743359 | 0.000465 | 2900052N01Rik | 3.782358 | 0.041588 | Acot11 | 2.846489 | 2.27E-28 | Acot11 | 2.846489 | 2.27E-28 |
| Tectb | 1.732361 | 5.35E-11 | Colq | 3.779493 | 0.006301 | Col10a1 | 2.841504 | 0.000106 | Col10a1 | 2.841504 | 0.000106 |
| Rmdn1 | 1.729205 | 0.001864 | Myb | 3.778021 | 0.012868 | Slc25a20 | 2.835908 | 1.44E-38 | Slc25a20 | 2.835908 | 1.44E-38 |
| Apold1 | 1.728299 | 4.21E-05 | Qrfp | 3.777483 | 0.010698 | Apoc2 | 2.835706 | 4.40E-19 | Apoc2 | 2.835706 | 4.40E-19 |
| Ppif | 1.724324 | 0.001228 | A530021J07Rik | 3.766082 | 0.004713 | Gyk | 2.833314 | 2.88E-63 | Gyk | 2.833314 | 2.88E-63 |
| Rbp7 | 1.724196 | 0.002003 | H2-DMb2 | 3.763688 | 0.023249 | Odf3l2 | 2.828751 | 0.019635 | Odf3l2 | 2.828751 | 0.019635 |
| Oscar | 1.716739 | 7.25E-11 | Bank1 | 3.7373 | 0.01151 | Bank1 | 3.7373 | 0.01151 | Bank1 | 3.7373 | 0.01151 |
| Tmem253 | 1.712513 | 0.000421 | Gm28693 | 3.731825 | 0.013968 | Gm28693 | 3.731825 | 0.013968 | Gm28693 | 3.731825 | 0.013968 |
| Acadvl | 1.712482 | 0.001412 | Pdk4 | 3.728832 | 0.000287 | Pdk4 | 3.728832 | 0.000287 | Pdk4 | 3.728832 | 0.000287 |
