# Supplementary material for: Isomeranzin activates Gnas-AMPK signaling to drive white adipose browning and curb obesity in mice
Source: EMBO Mol Med. 2025 Nov 26;18(1):55–90. doi: 10.1038/s44321-025-00335-y (PMC12808274; doi:10.1038/s44321-025-00335-y)
Supplement: Supplementary file 2 — Table EV2 [file 44321_2025_335_MOESM2_ESM.docx]

| ID | Molecular name | Score in cold-1 | Score in cold-2 | Score in CL316-1 | Score in CL316-1 |
| --- | --- | --- | --- | --- | --- |
| BRD-K87909389 | alvocidib | 98.24 | 97.25 | 98.39 | 98.34 |
| BRD-A60245366 | AS-601245 | 97.53 | 98.24 | 98.45 | 98.41 |
| BRD-K13390322 | AT-7519 | 98.2 | 98.13 | 98.24 | 98.38 |
| BRD-K13566078 | BMS-345541 | 97.92 | 97.71 | 97.99 | 97.92 |
| BRD-K79090631 | CGP-60474 | 98.41 | 97.78 | 98.24 | 98.06 |
| BRD-K43389675 | daunorubicin | 98.38 | 97.82 | 98.34 | 98.38 |
| BRD-K11927976 | ER-27319 | 98.27 | 98.2 | 98.38 | 98.34 |
| BRD-A73909368 | isomerazin | 98.03 | 98.17 | 98.34 | 98.24 |
| BRD-K11636097 | JNJ-7706621 | 98.1 | 96.61 | 98.1 | 98.1 |
| BRD-K21680192 | mitoxantrone | 97.89 | 97.99 | 98.09 | 98.24 |
| BRD-K83794624 | pirarubicin | 97.88 | 97.78 | 98.27 | 98.34 |

Table EV2-candidate compounds
