# Supplementary material for: Isomeranzin activates Gnas-AMPK signaling to drive white adipose browning and curb obesity in mice
Source: EMBO Mol Med. 2025 Nov 26;18(1):55–90. doi: 10.1038/s44321-025-00335-y (PMC12808274; doi:10.1038/s44321-025-00335-y)
Supplement: Supplementary file 3 — Table EV3 [file 44321_2025_335_MOESM3_ESM.docx]

Table EV3-potential target proteins

| Peptide Sequence | Protein ID | Gene name | p-Value |
| --- | --- | --- | --- |
| IVGLIQK | O08529 | Capn2 | 0.02323 |
| ATEDYFDPK | O35604 | Npc1 | 0.046342 |
| LEPLIPR | O55222 | Ilk | 0.028412 |
| DHGDWDVDR | O88668 | Creg1 | 0.038256 |
| GYVDFESAEDLEK | P09405 | Ncl | 0.024983 |
| HVWFGES | P11087 | Col1a1 | 0.020389 |
| AYNPDEEEDDAESR | P31324 | Prkar2b | 0.008563 |
| ELPIVTPALR | P52293 | Kpna2 | 0.003403 |
| EKSYELPDGQVITIGNER | P60710 | Actb | 0.045259 |
| IEDYFPEFAR | P63094 | Gnas | 0.01085 |
| GEHPGLSIGDVA | P63158 | Hmgb1 | 0.043526 |
| GNPTPMLEWIGGPSGQLPAK | Q05793 | Hspg2 | 0.045988 |
| DLDECALKPSVC | Q08761 | Pros1 | 0.021159 |
| KYFPETWIW | Q61838 | Pzp | 0.001542 |
| DITTGQPPR | Q6P5E4 | Uggt1 | 0.02894 |
| LEGEDSAQETPIGLVPK | Q8BH04 | Pck2 | 0.044377 |
| GIIDLIEER | Q8K0D5 | Gfm1 | 0.023647 |
| FVNPAGEVSAPSYPGYLR | Q8R1X6 | Spart | 0.009254 |
| IEPGVDPDDTYNETPYEK | Q8VCT3 | Rnpep | 0.025495 |
| KQELEEICHDLEAR | Q8VDD5 | Myh9 | 0.042923 |
| GMPITEVFK | Q91V92 | Acly | 0.042283 |
| KIEIDNGDELTAD | Q91YR1 | Twf1 | 0.039946 |
| GLPDLQR | Q9D2R0 | Aacs | 0.003254 |
| FLEQNYDTIFEDYEK | Q9DB16 | Cab39l | 0.002342 |
| KPSETQELVQ | Q9DBG3 | Ap2b1 | 0.006149 |
| FAPPDTSDMEAVWK | Q9QY76 | Vapb | 0.015964 |
