# Supplementary material for: Isomeranzin activates Gnas-AMPK signaling to drive white adipose browning and curb obesity in mice
Source: EMBO Mol Med. 2025 Nov 26;18(1):55–90. doi: 10.1038/s44321-025-00335-y (PMC12808274; doi:10.1038/s44321-025-00335-y)
Supplement: Supplementary file 4 — Table EV4 [file 44321_2025_335_MOESM4_ESM.docx]

Table EV4-Primers Sequence

| **Primer** | **Sequence（5’–3’）** |
| --- | --- |
| 18S-Forward | TAAACGATGCCGACTGGCGA |
| 18S-Reverse | CAAATTAAGCCGCAGGCCCA |
| Mouse-Ucp1-Forward | TGGTGAACCCGACAACTTCC |
| Mouse-Ucp1- Reverse | GGCCTTCACCTTGGATCTGAA |
| Mouse- Pgc1α-Forward | CCCTGCCATTGTTAAGACC |
| Mouse- Pgc1α-Reverse | TGCTGCTGTTCCTGTTTTC |
| Mouse- Prdm16-Forward | GACATTCCAATCCCACCAGA |
| Mouse- Prdm16-Reverse | CACCTCTGTATCCGTCAGCA |
| Mouse- Cidea-Forward | ATCACAACTGGCCTGGTTACG |
| Mouse- Cidea-Reverse | TACTACCCGGTGTCCATTTCT |
| Mouse- Dio2-Forward | CAGTGTGGTGCACGTCTCCAATC |
| Mouse- Dio2-Reverse | TGAACCAAAGTTGACCACCAG |
| Mouse- Elvol3-Forward | TGGAGGAGTACTGGGTAAGC |
| Mouse- Elvol3-Reverse | GTAGGTCTGGCCAACAACGA |
| Mouse- Pparα-Forward | AGAGCCCCATCTGTCCTCTC |
| Mouse- Pparα-Reverse | ACTGGTAGTCTGCAAAACCAAA |
| Mouse- Acox1-Forward | GGTGGACATTAACAGCCTGGACAG |
| Mouse- Acox1-Reverse | CACGCCACTTCCTTGCTCTTCC |
| Mouse-Cox4b-Forward | GTGGCAGCGAGTCTATGTGT |
| Mouse-Cox4b-Reverse | CGTGGGACGTTGGTCACTTT |
| Mouse-Atp5a1-Forward | GCCATTTTGTGCCAGTCGT |
| Mouse-Atp5a1-Reverse | TGCTTAACACACGCCCAGTC |
| Mouse-MCAD-Forward | AAAAGAGCCTGGGAACTCGG |
| Mouse-MCAD-Reverse | CCATACGCCAACTCTTCGGT |
| Mouse-LCAD-Forward | CCGCCCGATGTTCTCATTCT |
| Mouse-LCAD-Reverse | CGCCATGTTTCTCTGCGATG |
| Mouse-Cpt1a-Forward | AGATCAATCGGACCCTAGACAC |
| Mouse-Cpt1a-Reverse | CAGCGAGTAGCGCATAGTCA |
| Mouse- Pparγ-Forward | GTGCCAGTTTCGATCCGTAGA |
| Mouse- Pparγ-Reverse | GGCCAGCATCGTGTAGATGA |
| Mouse- C/Ebpα-Forward | CAAGAACAGCAACGAGTACCG |
| Mouse- C/Ebpα-Reverse | GTCACTCGTCAACTCCAGCAC |
| Mouse- Fabp4-Forward | CAAGAACAGCAACGAGTACCG |
| Mouse- Fabp4-Reverse | GTCACTCGTCAACTCCAGCAC |
| Mouse- Gnas-Forward | CAACAGTAAGACCGAGGACCA |
| Mouse- Gnas-Reverse | TAGTGGCCTTCTCACCATCG |
| Human-Ucp1-Forward | AGGTCCAAGGTGAATGCCC |
| Human-Ucp1- Reverse | TTACCACAGCGGTGATTGTTC |
| Human- Pgc1α-Forward | TCTGAGTCTGTATGGAGTGACAT |
| Human- Pgc1α-Reverse | CCAAGTCGTTCACATCTAGTTCA |
| Human- Prdm16-Forward | CGAGGCCCCTGTCTACATTC |
| Human- Prdm16-Reverse | GCTCCCATCCGAAGTCTGTC |
| Human- Cidea-Forward | CTTGGGAGACAACACGCATTT |
| Human- Cidea-Reverse | TCTCGCTATTCCCGACCTCTT |
| Human- Dio2-Forward | TCCTCCTCGATGCCTACAAAC |
| Human- Dio2-Reverse | GTGAGTAGACCAGTAGTCTGCT |
| Human- Elvol3-Forward | TGGGGCATTATGGGGACTGT |
| Human- Elvol3-Reverse | AGGACCAGAATTTGACTGTGGA |
| Human- Pparα-Forward | TTCGCAATCCATCGGCGAG |
| Human- Pparα-Reverse | CCACAGGATAAGTCACCGAGG |
| Human- Acox1-Forward | AATCGGGACCCATAAGCCTTT |
| Human- Acox1-Reverse | GGGAATACGATGGTTGTCCATTT |
| Human-Cox4b-Forward | ACTACCCCATGCCAGAAGAG |
| Human-Cox4b-Reverse | TCATTGGAGCGACGGTTCATC |
| Human-Atp5a1-Forward | AACTGATTATTGGTGACCGACAG |
| Human-Atp5a1-Reverse | GGCAACAGTGGATCTCTTTTGA |
| Human-MCAD-Forward | TGGATAACCAACGGAGGAAAAG |
| Human-MCAD-Reverse | CTGGGGTATCTGCTTCCACA |
| Human-LCAD-Forward | TGCAATAGCAATGACAGAGCC |
| Human-LCAD-Reverse | CGCAACTACAATCACAACATCAC |
| Human-Cpt1a-Forward | ATCAATCGGACTCTGGAAACGG |
| Human-Cpt1a-Reverse | TCAGGGAGTAGCGCATGGT |
| Human-Nrf1-Forward | AGGAACACGGAGTGACCCAA |
| Human-Nrf1-Reverse | TATGCTCGGTGTAAGTAGCCA |
| Human-Tfam-Forward | GCGGGTTCCAGTTGTGATTG |
| Human-Tfam-Reverse | CCCCACATGCTTCGGAGAAA |
| Human- C/Ebpα-Forward | AAGCACGATCAGTCCATCCC |
| Human- C/Ebpα-Reverse | GGCACAGAGGCCAGATACAA |
| Human- Fabp4-Forward | ACTGGGCCAGGAATTTGACG |
| Human- Fabp4-Reverse | CTCGTGGAAGTGACGCCTT |
