# Supplementary material for: Isomeranzin activates Gnas-AMPK signaling to drive white adipose browning and curb obesity in mice
Source: EMBO Mol Med. 2025 Nov 26;18(1):55–90. doi: 10.1038/s44321-025-00335-y (PMC12808274; doi:10.1038/s44321-025-00335-y)
Supplement: Supplementary file 5 — Table EV5 [file 44321_2025_335_MOESM5_ESM.docx]

Table EV5-siRNA Sequence

| GNas (house mouse) siRNA-2717 | ACAAGAUUGAUGUGAUCAATT |
| --- | --- |
|  | UUGAUCACAUCAAUCUUGUTT |
