# Supplementary figures and images for: Isomeranzin activates Gnas-AMPK signaling to drive white adipose browning and curb obesity in mice
Source: EMBO Mol Med. 2025 Nov 26;18(1):55–90. doi: 10.1038/s44321-025-00335-y (PMC12808274; doi:10.1038/s44321-025-00335-y)

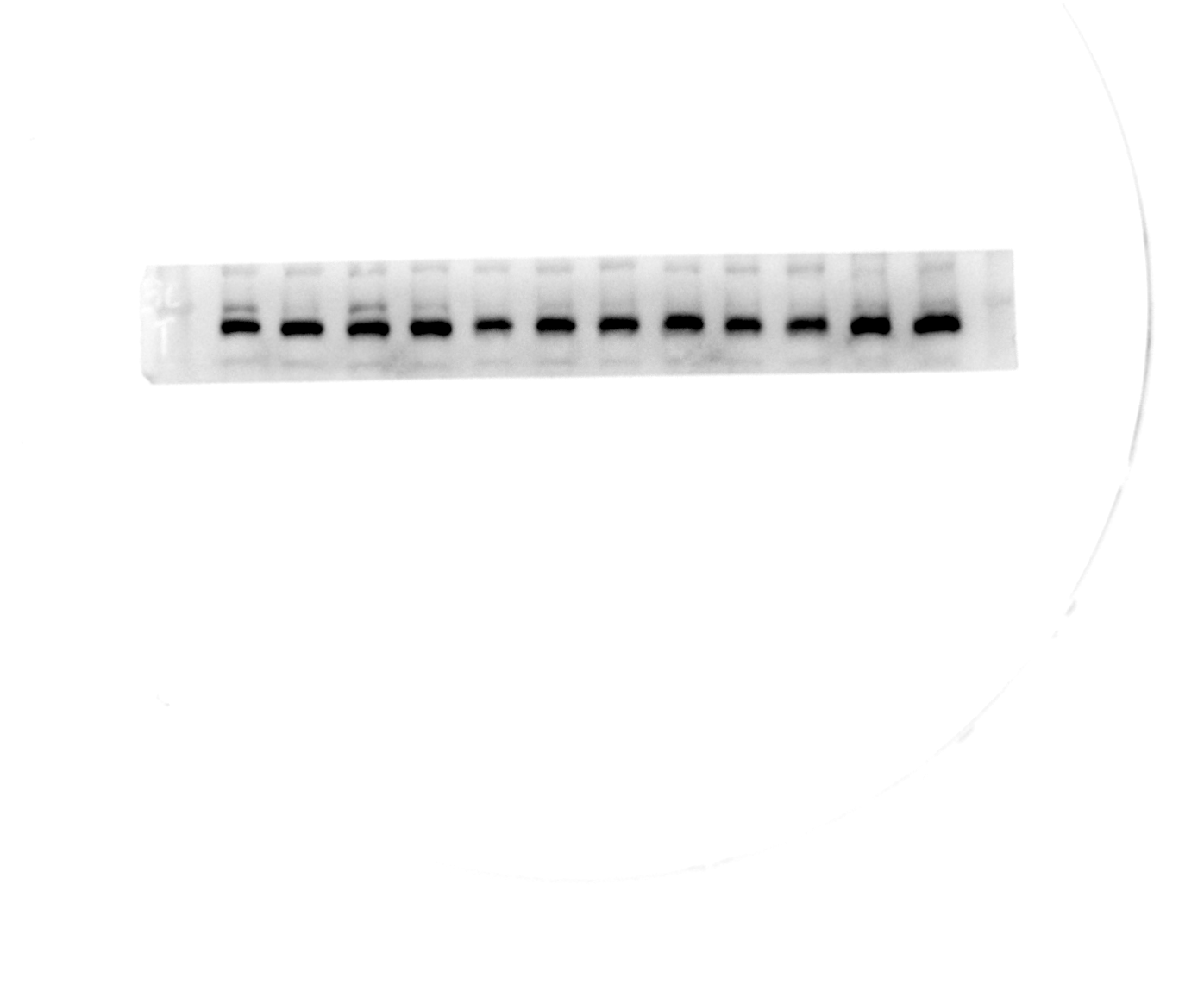

Supplement: Supplementary file 7 — Source data Fig. 1 [file 44321_2025_335_MOESM7_ESM.zip › Figure 1/Figure 1-D/western Tubulin.tif]

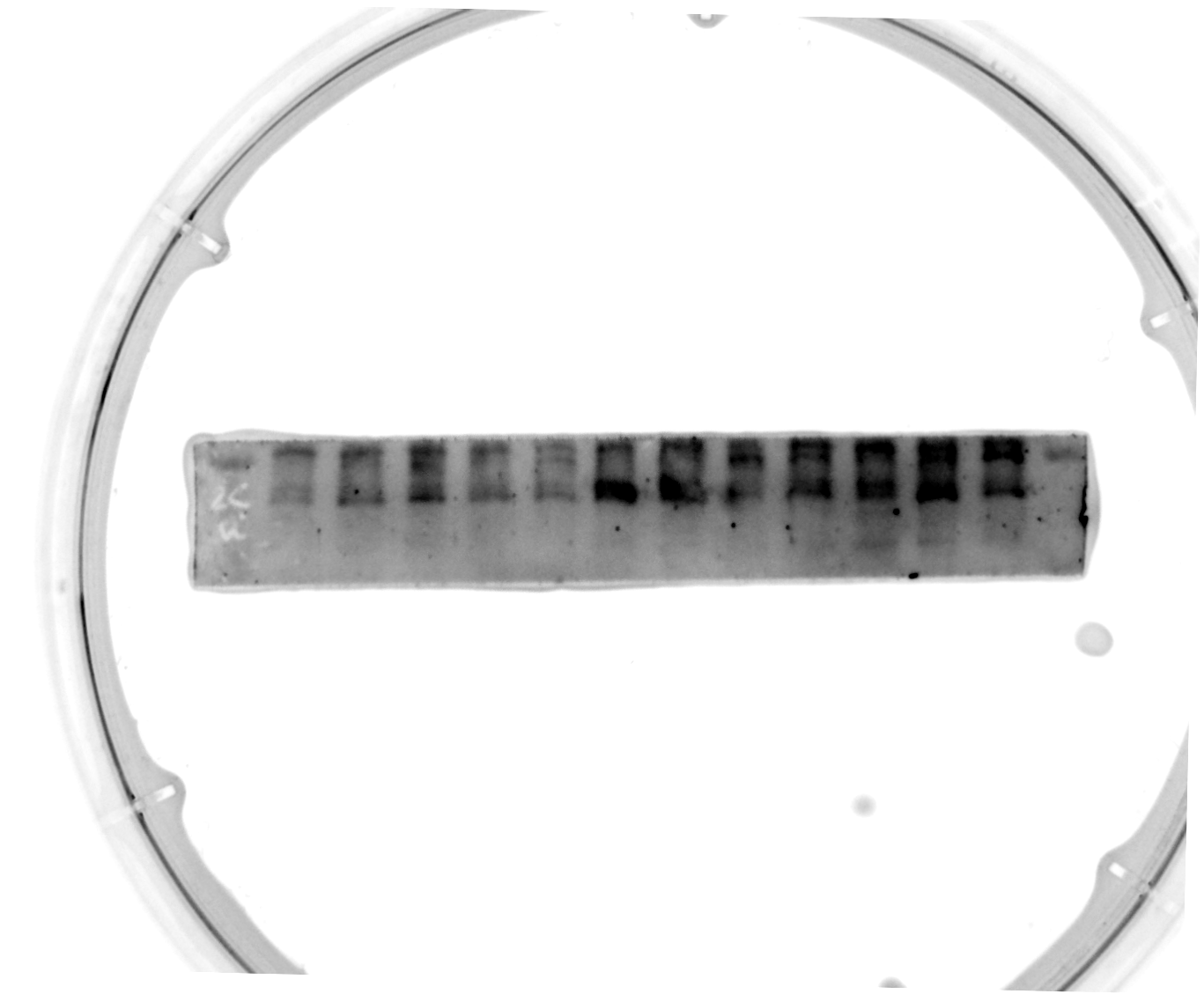

Supplement: Supplementary file 7 — Source data Fig. 1 [file 44321_2025_335_MOESM7_ESM.zip › Figure 1/Figure 1-D/western Ucp1.tif]

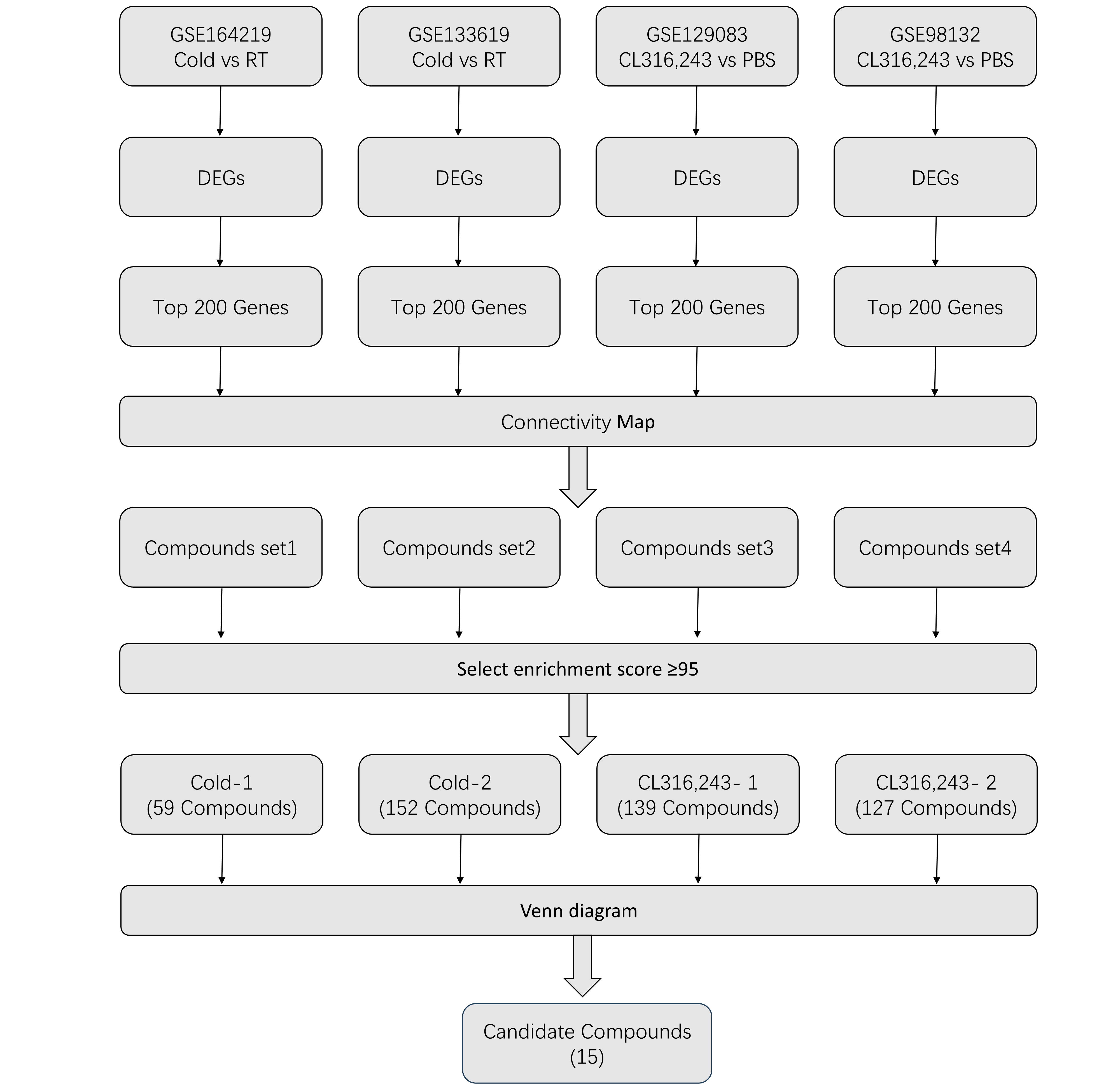

Supplement: Supplementary file 7 — Source data Fig. 1 [file 44321_2025_335_MOESM7_ESM.zip › Figure 1/Figure 1-A/Workflow diagram.tif]

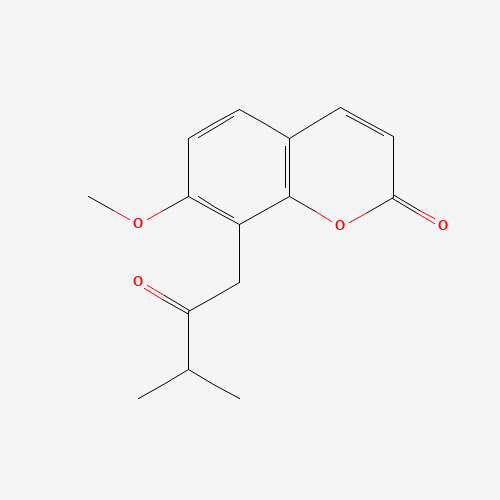

Supplement: Supplementary file 8 — Source data Fig. 2 [file 44321_2025_335_MOESM8_ESM.zip › Figure 2/Figure 2-A/Chemical Structure Depiction.png]

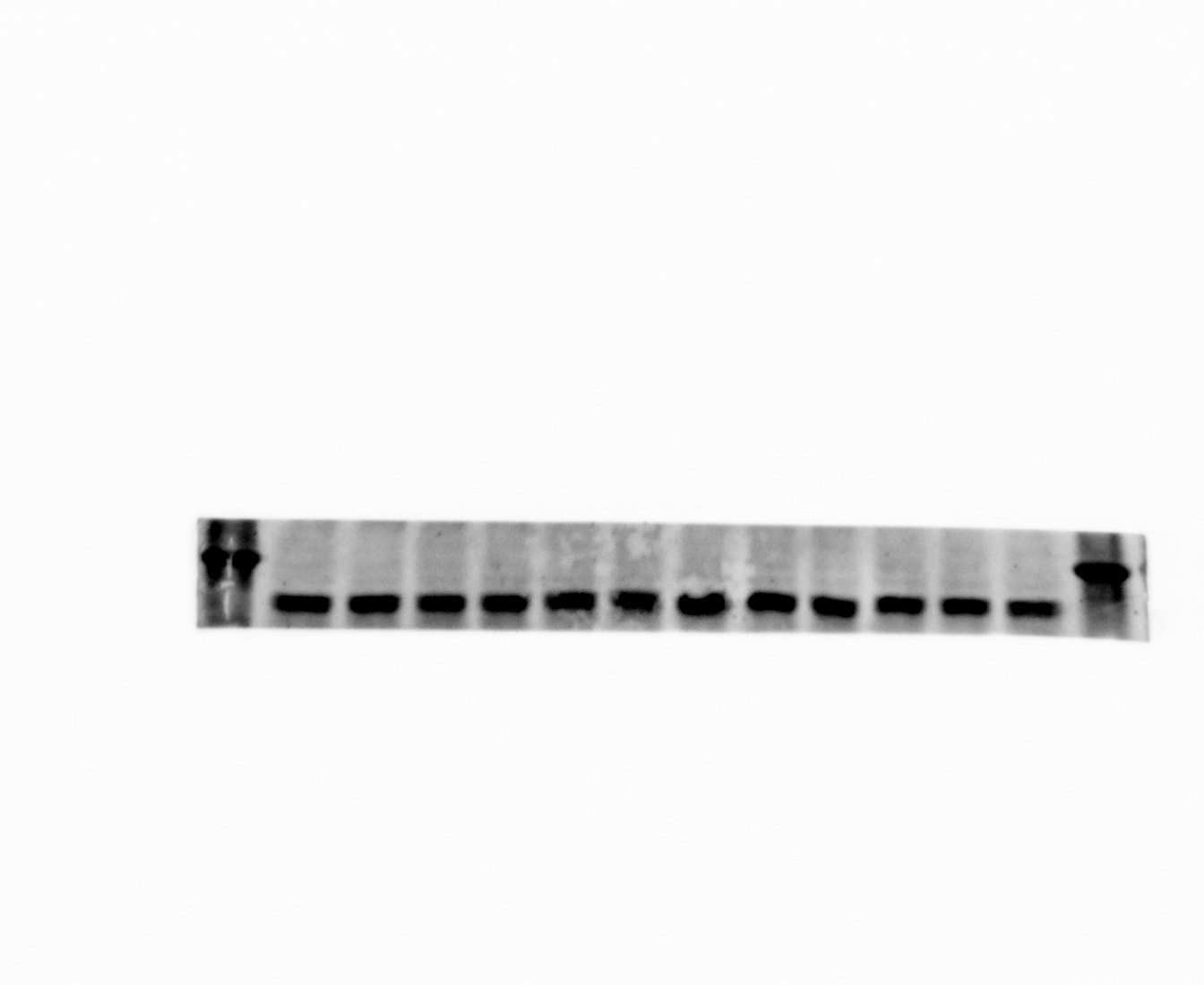

Supplement: Supplementary file 8 — Source data Fig. 2 [file 44321_2025_335_MOESM8_ESM.zip › Figure 2/Figure 2-E/western-tubulin.tif]

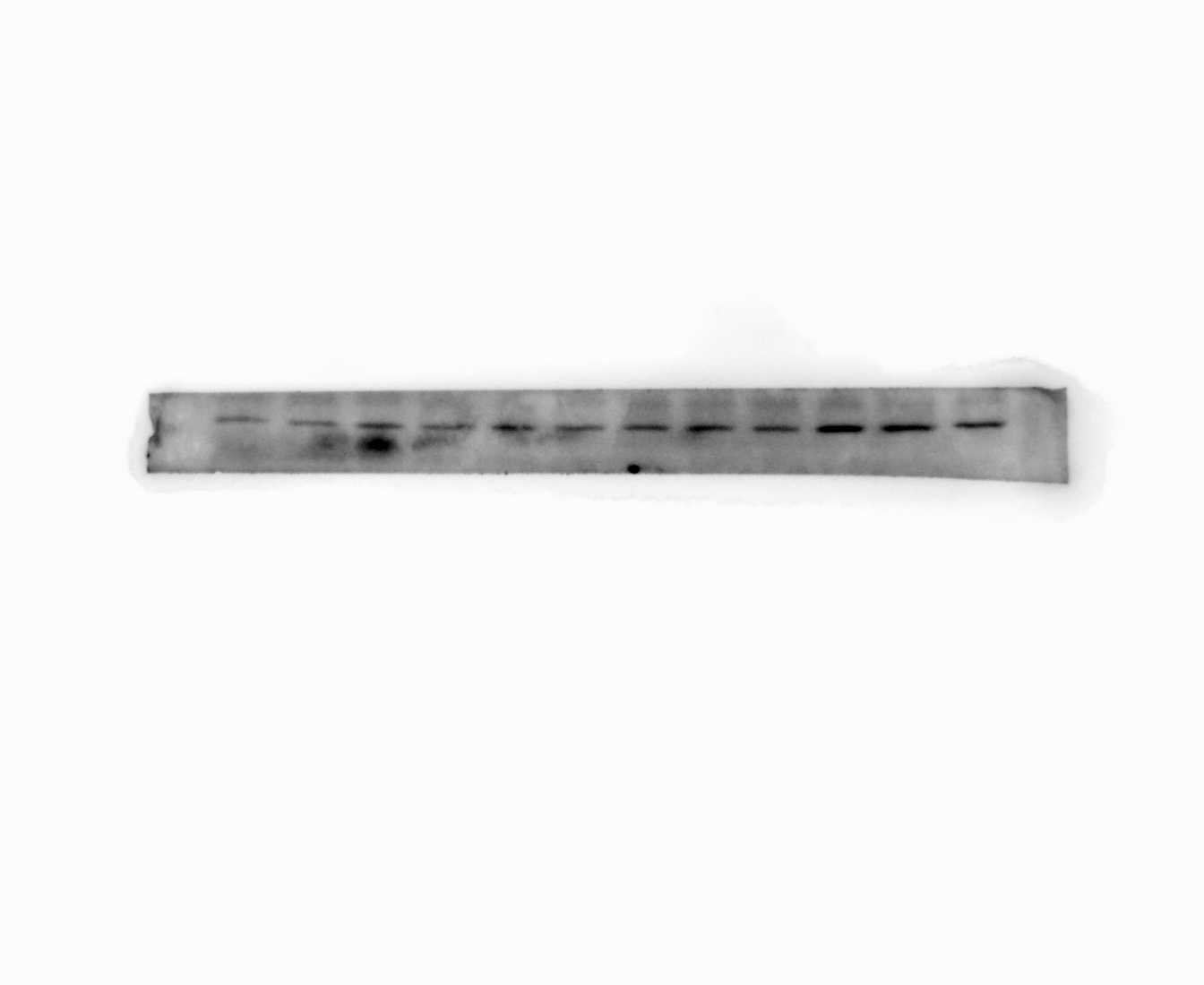

Supplement: Supplementary file 8 — Source data Fig. 2 [file 44321_2025_335_MOESM8_ESM.zip › Figure 2/Figure 2-E/western-ucp1.tif]

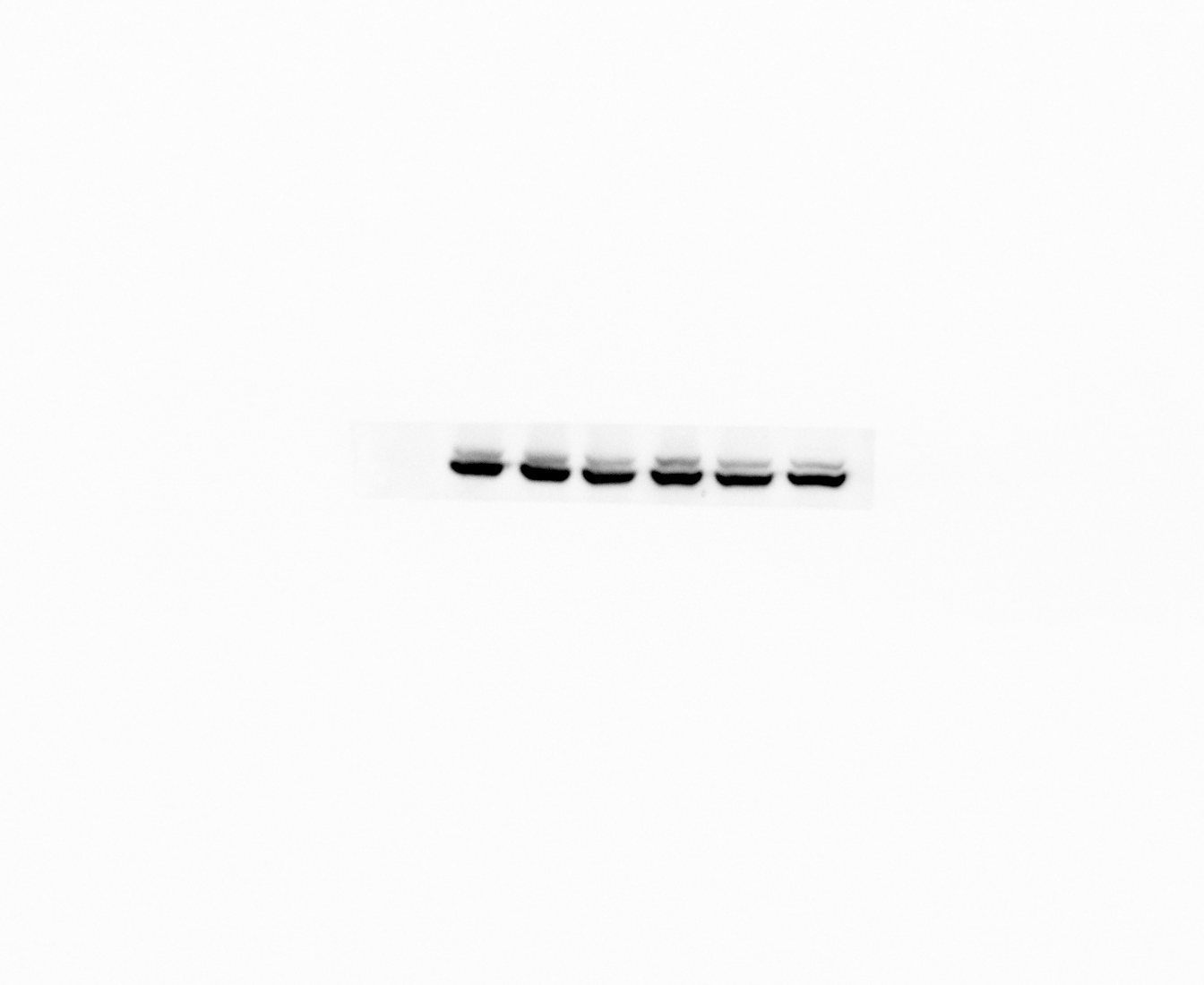

Supplement: Supplementary file 8 — Source data Fig. 2 [file 44321_2025_335_MOESM8_ESM.zip › Figure 2/Figure 2-F/western-Hsp90.tif]

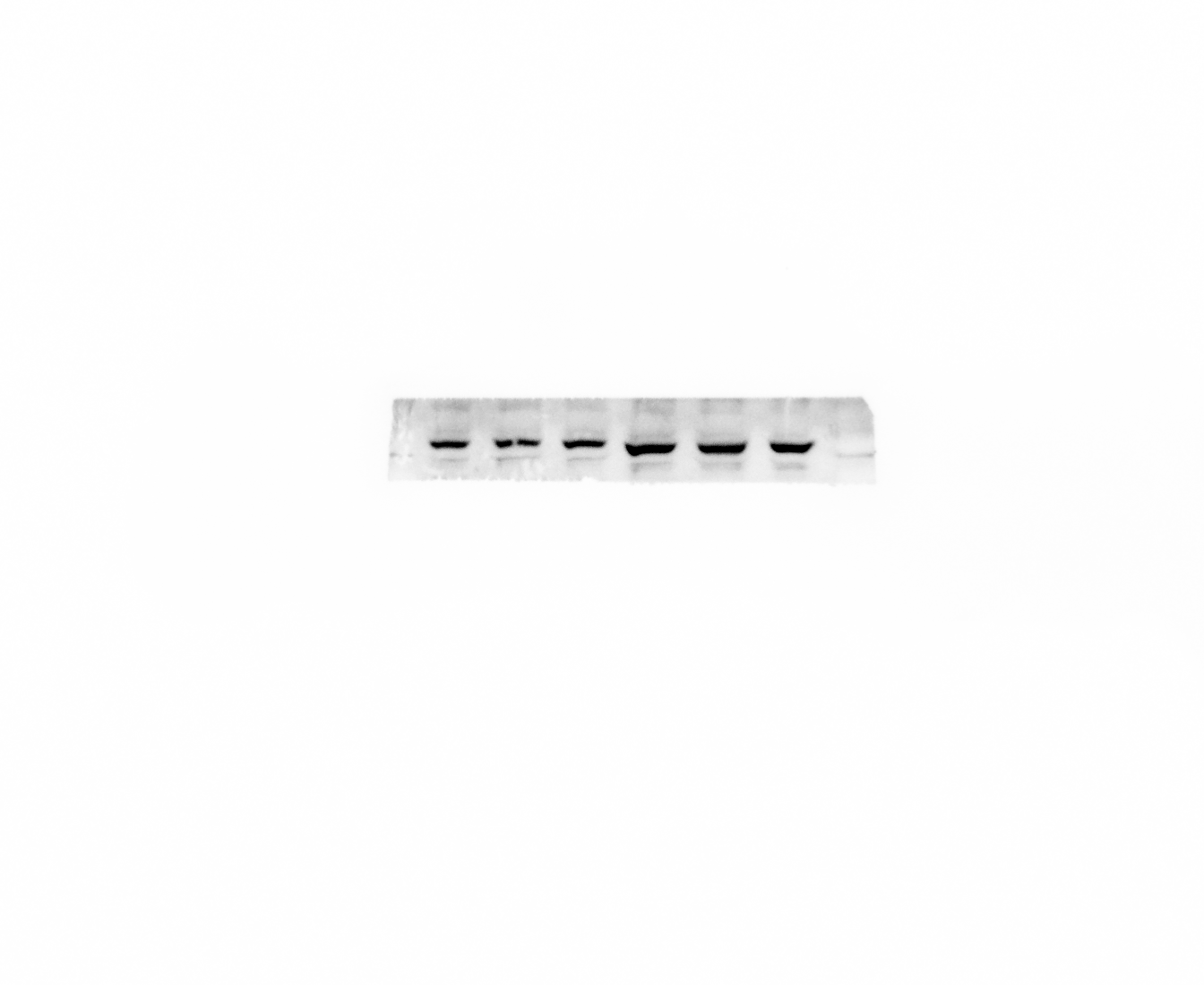

Supplement: Supplementary file 8 — Source data Fig. 2 [file 44321_2025_335_MOESM8_ESM.zip › Figure 2/Figure 2-F/western-Pgc1α.tif]

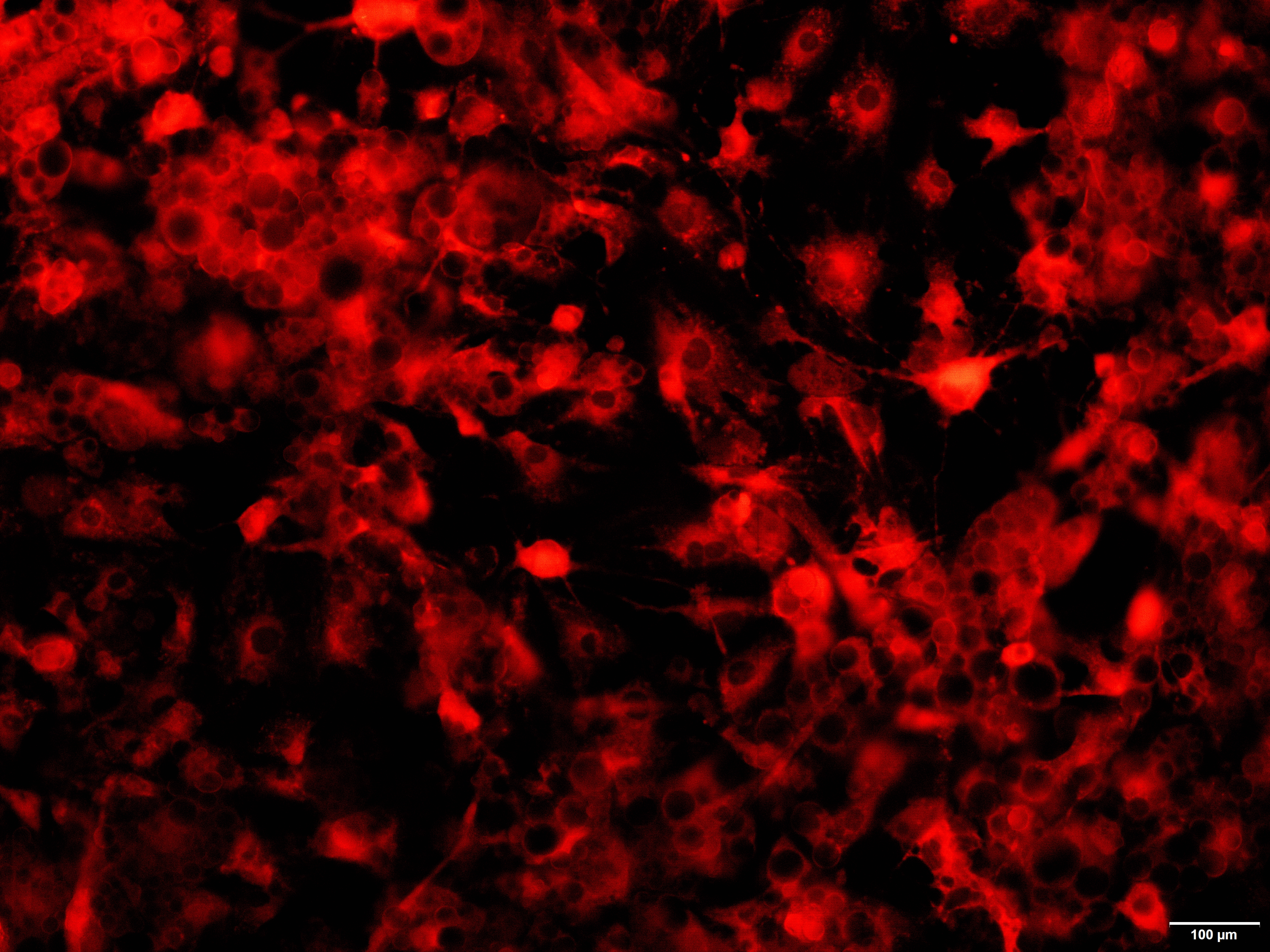

Supplement: Supplementary file 8 — Source data Fig. 2 [file 44321_2025_335_MOESM8_ESM.zip › Figure 2/Figure 2-H/Mitotracker staining Control-20X.jpg]

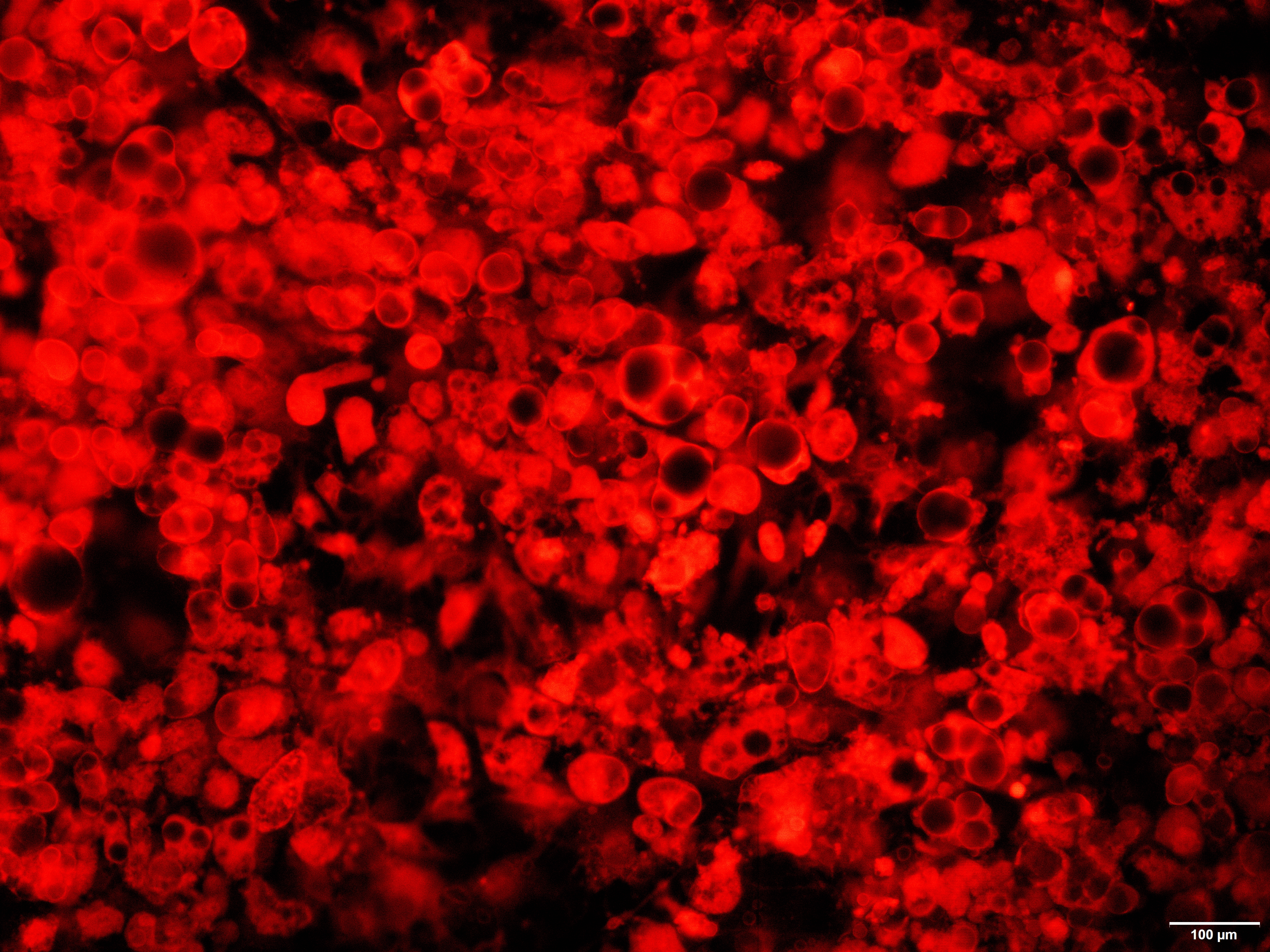

Supplement: Supplementary file 8 — Source data Fig. 2 [file 44321_2025_335_MOESM8_ESM.zip › Figure 2/Figure 2-H/Mitotracker staining-ISM-20X.jpg]

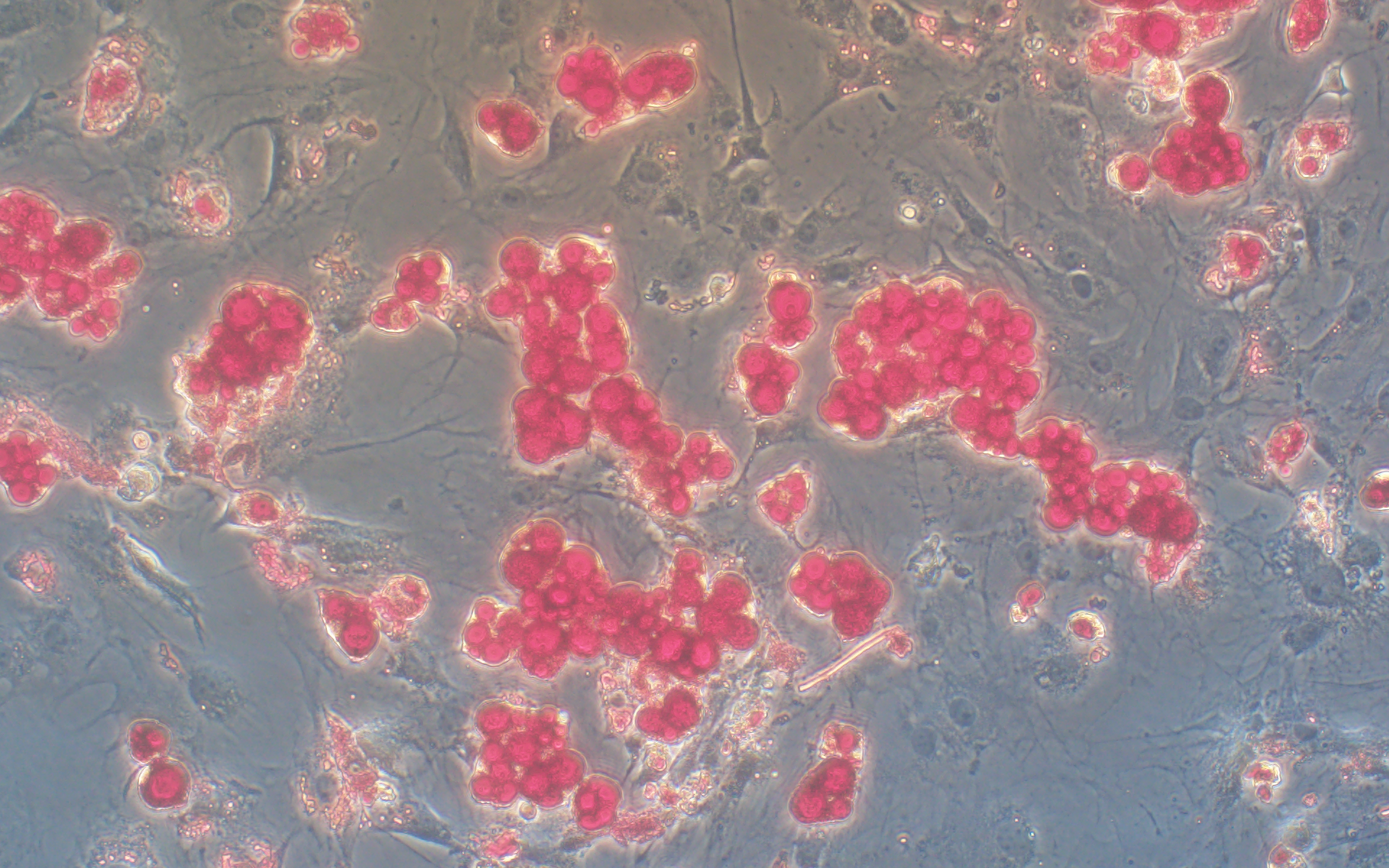

Supplement: Supplementary file 8 — Source data Fig. 2 [file 44321_2025_335_MOESM8_ESM.zip › Figure 2/Figure 2-J/Oil Red O staining Control.tif]

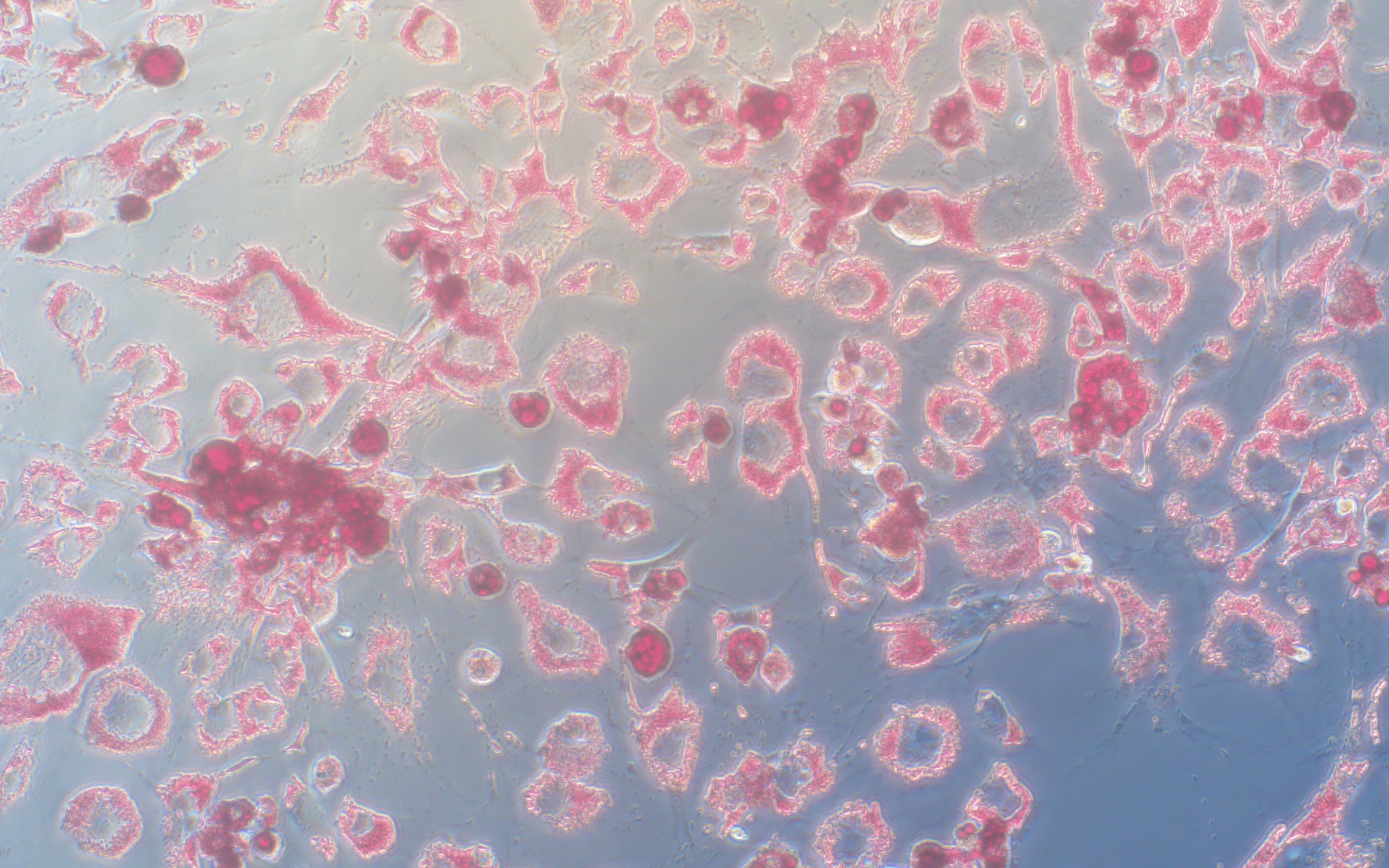

Supplement: Supplementary file 8 — Source data Fig. 2 [file 44321_2025_335_MOESM8_ESM.zip › Figure 2/Figure 2-J/Oil Red O staining ISM.tif]

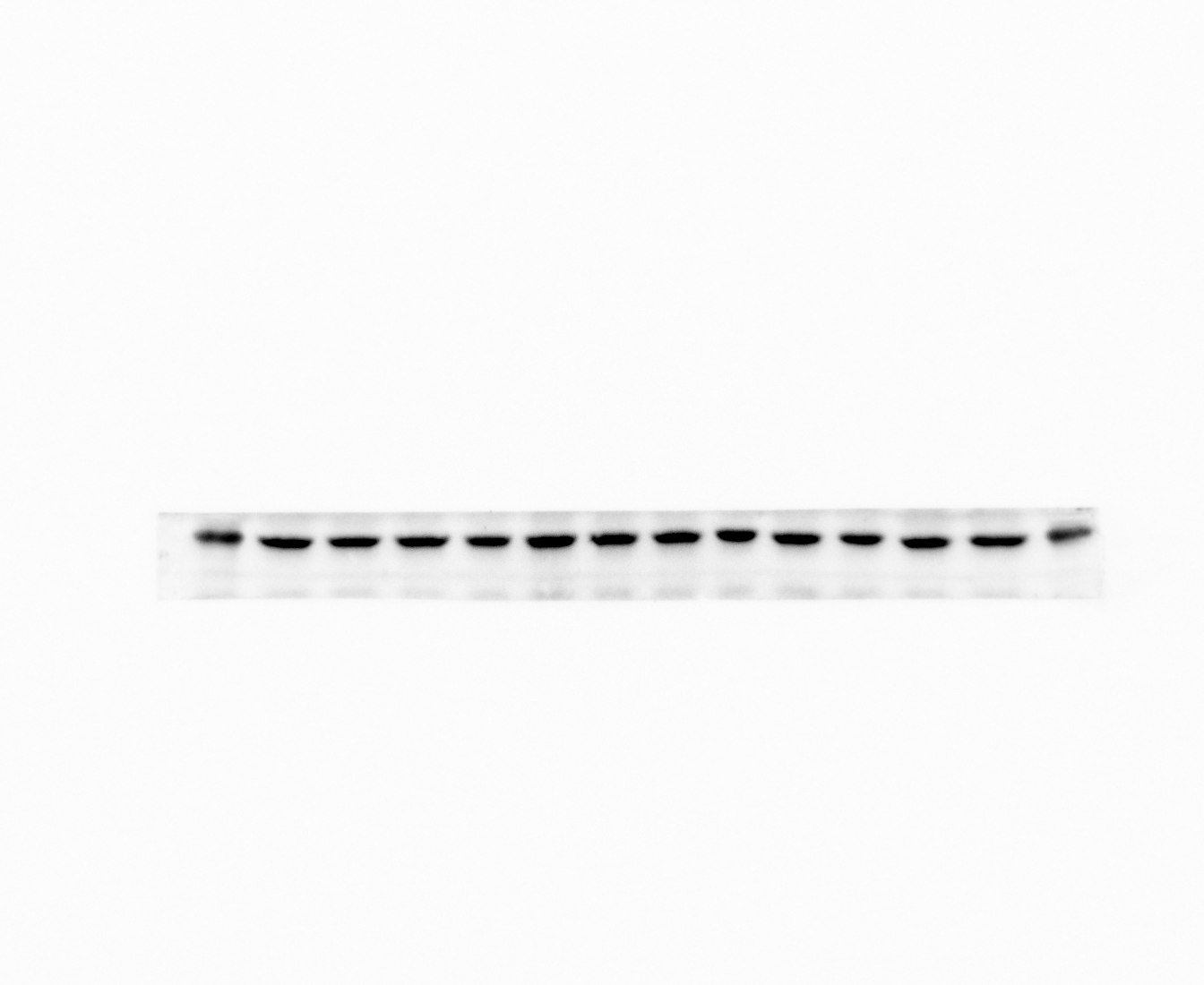

Supplement: Supplementary file 8 — Source data Fig. 2 [file 44321_2025_335_MOESM8_ESM.zip › Figure 2/Figure 2-P/western CEbpα.tif]

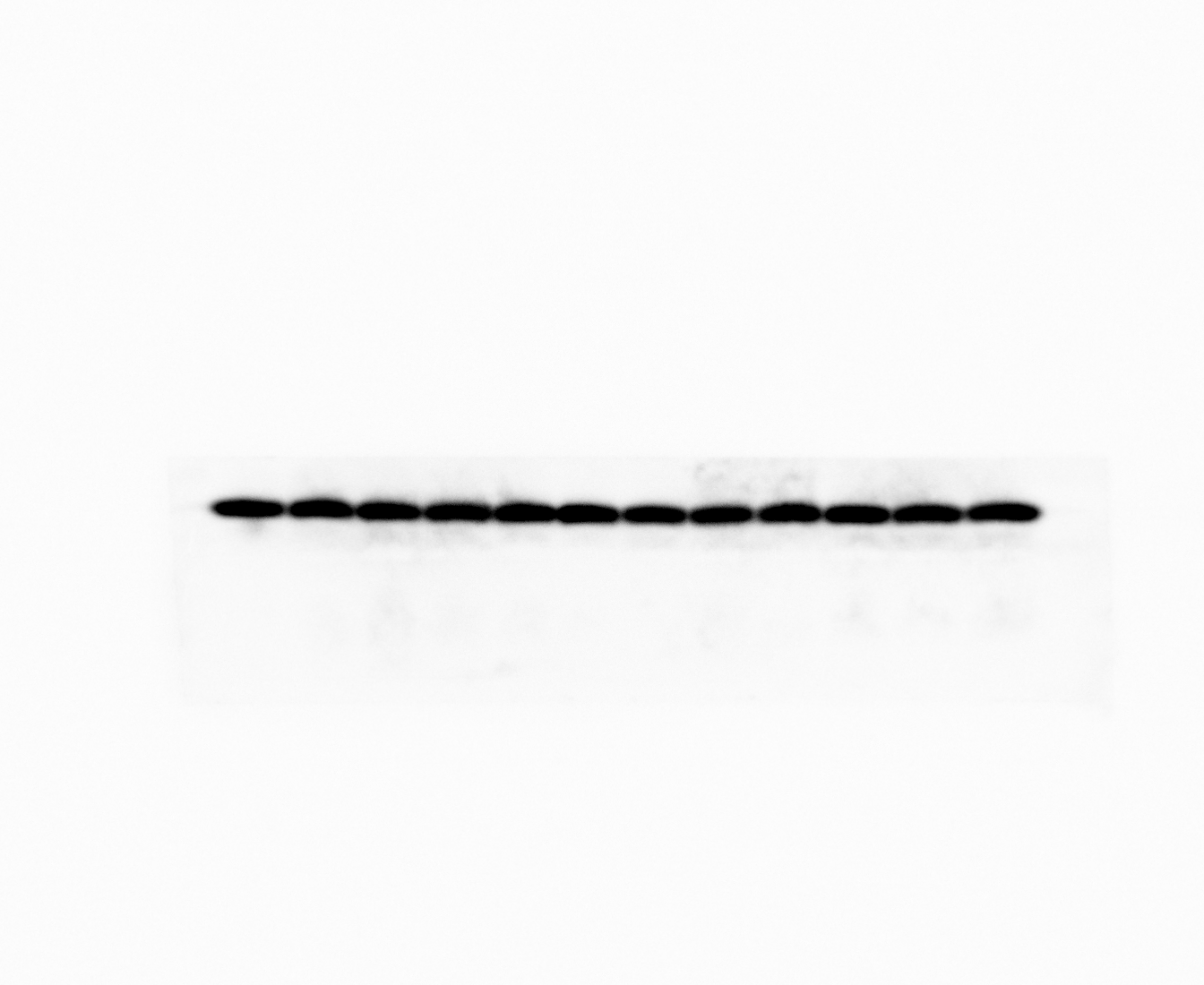

Supplement: Supplementary file 8 — Source data Fig. 2 [file 44321_2025_335_MOESM8_ESM.zip › Figure 2/Figure 2-P/western Fabp4.tif]

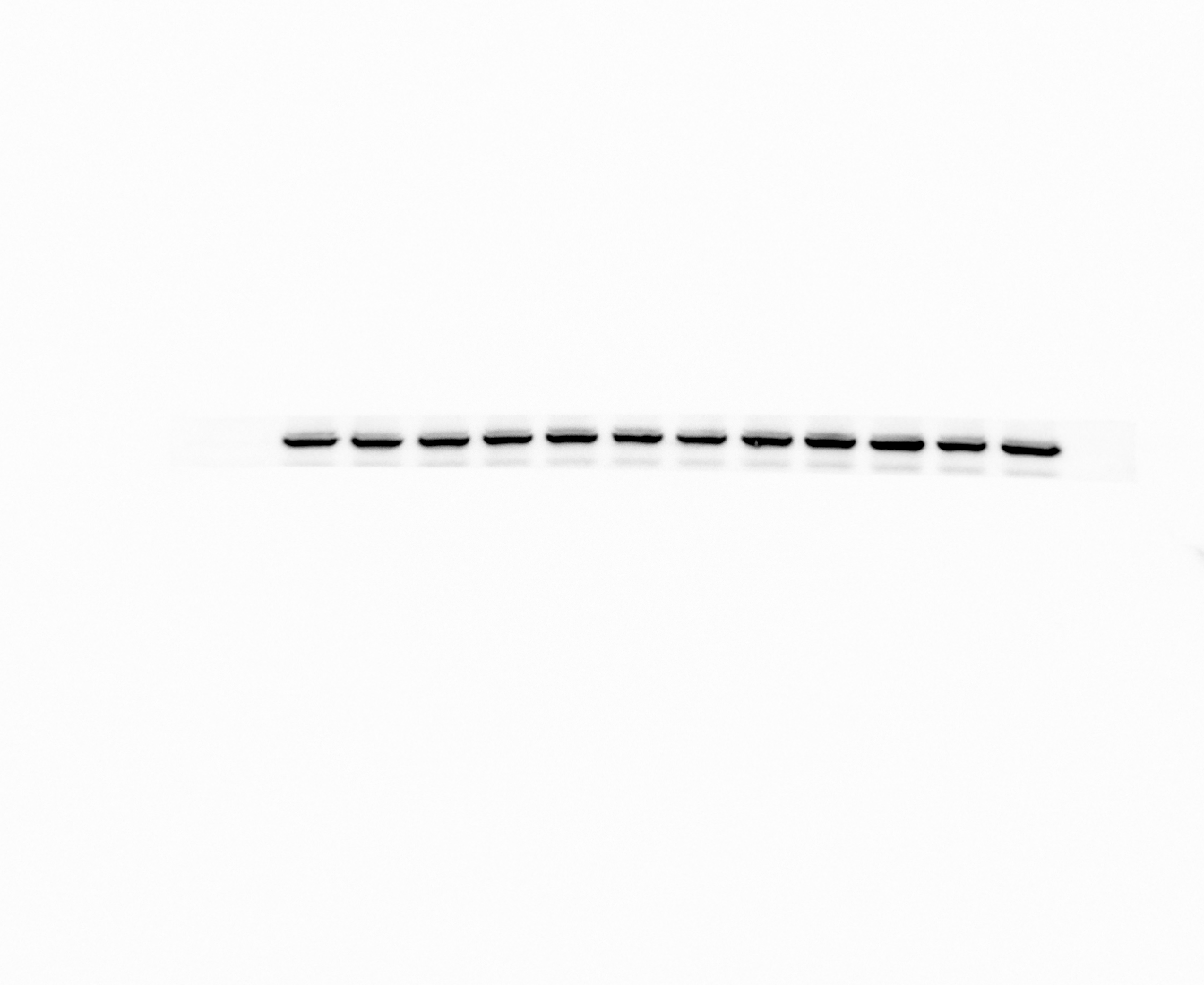

Supplement: Supplementary file 8 — Source data Fig. 2 [file 44321_2025_335_MOESM8_ESM.zip › Figure 2/Figure 2-P/western Hsp90.tif]

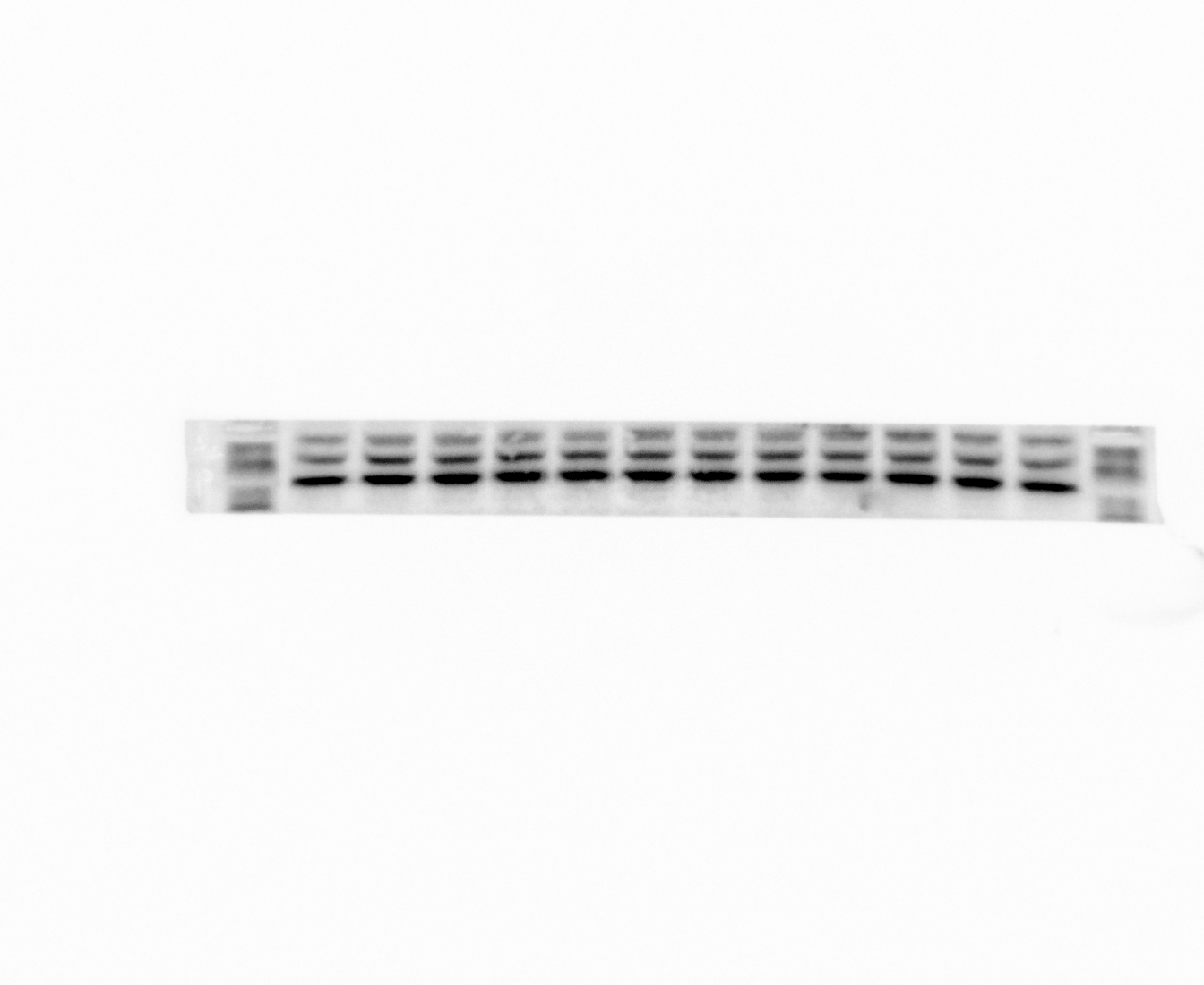

Supplement: Supplementary file 8 — Source data Fig. 2 [file 44321_2025_335_MOESM8_ESM.zip › Figure 2/Figure 2-P/western-Pparγ.tif]

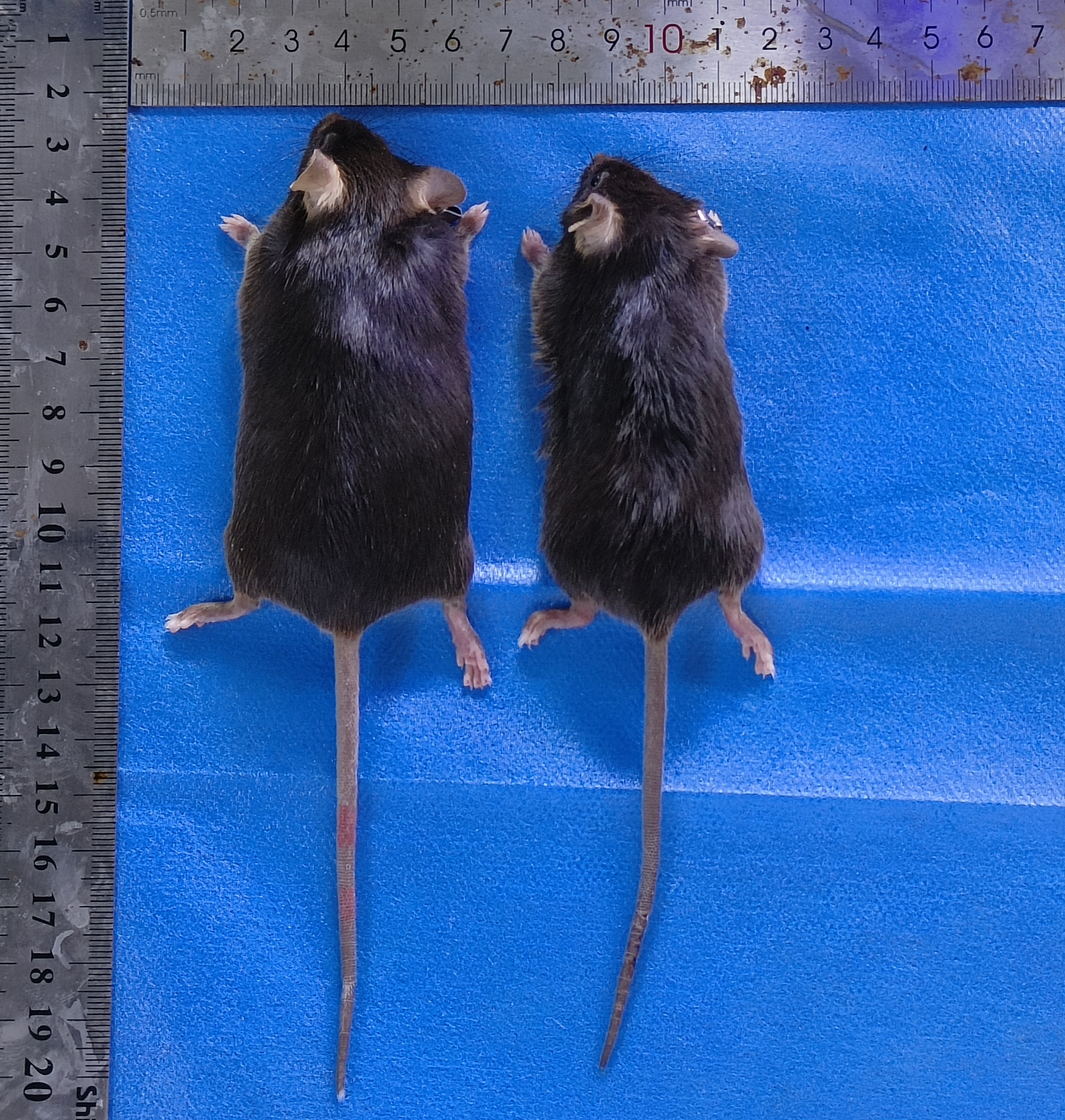

Supplement: Supplementary file 9 — Source data Fig. 3 [file 44321_2025_335_MOESM9_ESM.zip › Figure 3/Figure 3-B/Representative gross images of mice.jpg]

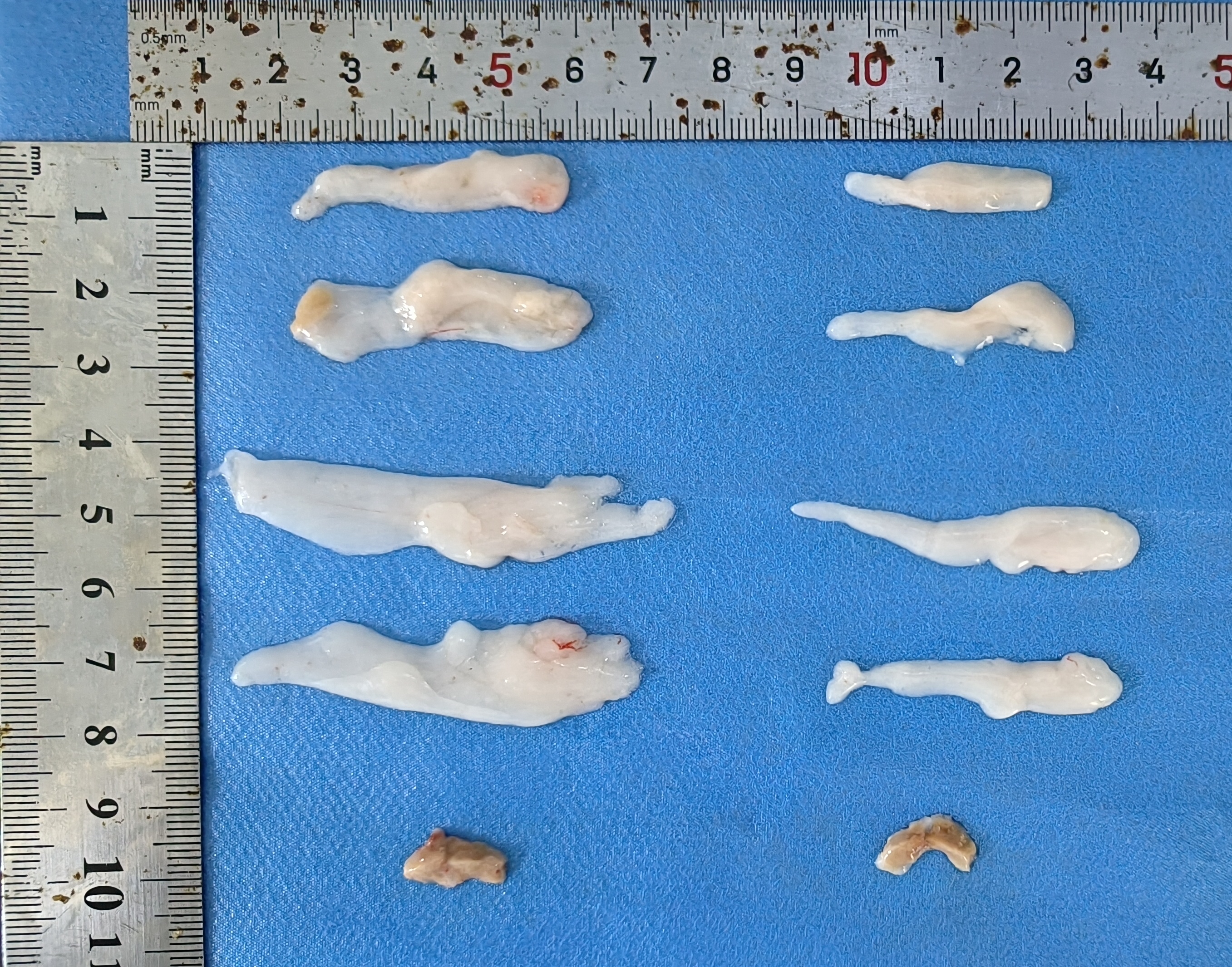

Supplement: Supplementary file 9 — Source data Fig. 3 [file 44321_2025_335_MOESM9_ESM.zip › Figure 3/Figure 3-D/Representative gross images of adipose tissues.jpg]

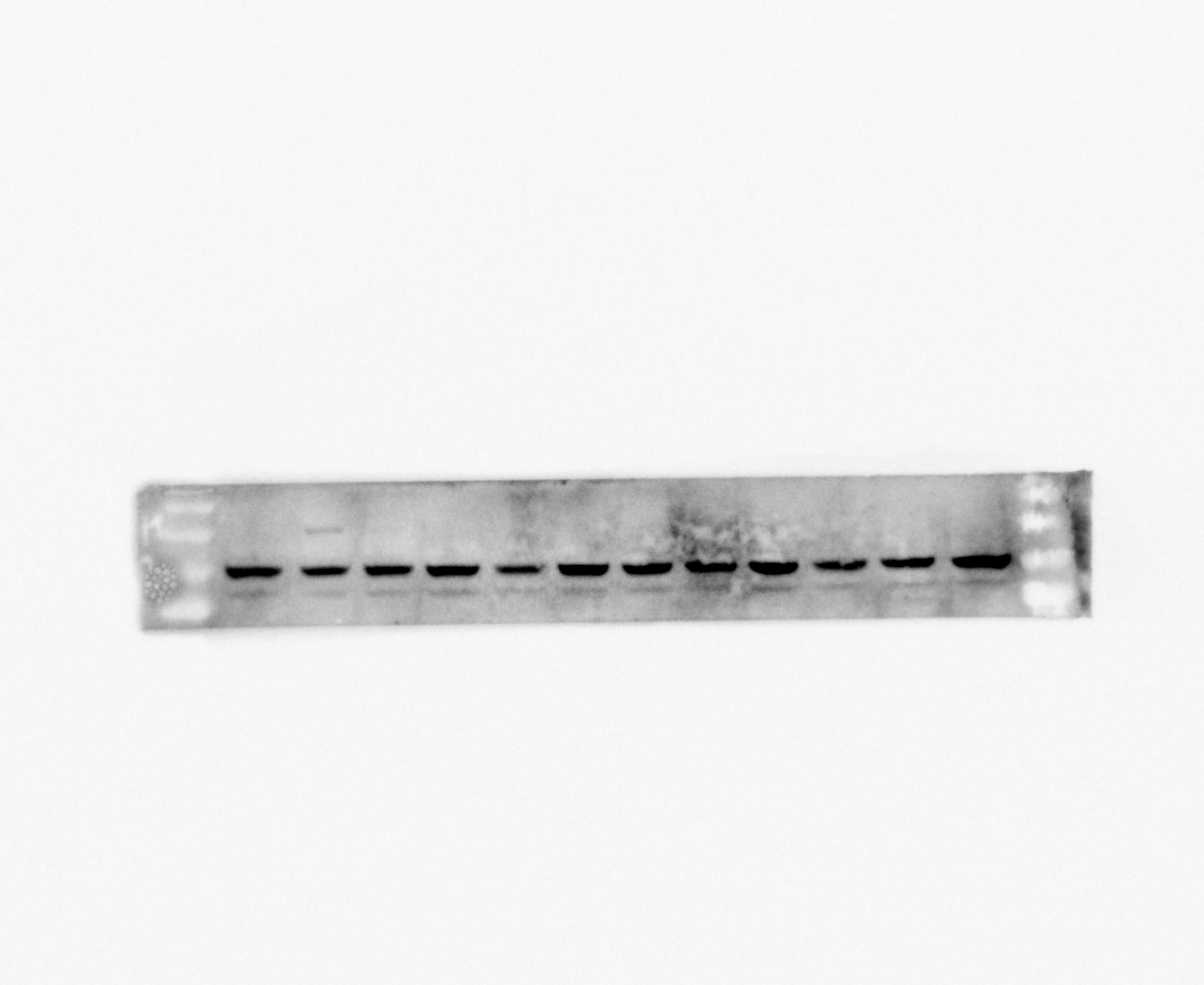

Supplement: Supplementary file 10 — Source data Fig. 4 [file 44321_2025_335_MOESM10_ESM.zip › Figure 4/Figure 4-F/western Pgc1╬▒.tif]

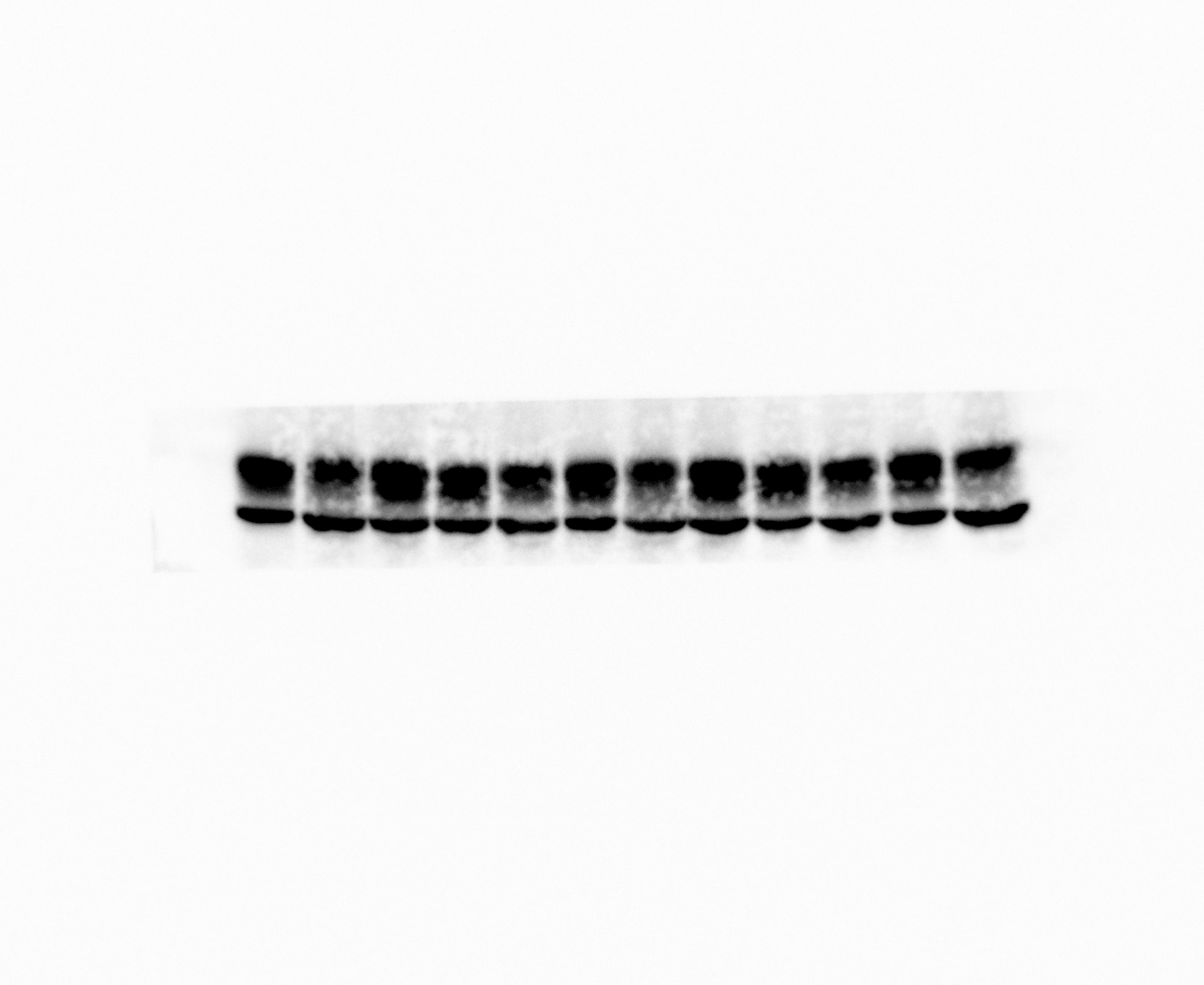

Supplement: Supplementary file 10 — Source data Fig. 4 [file 44321_2025_335_MOESM10_ESM.zip › Figure 4/Figure 4-F/western actin.tif]

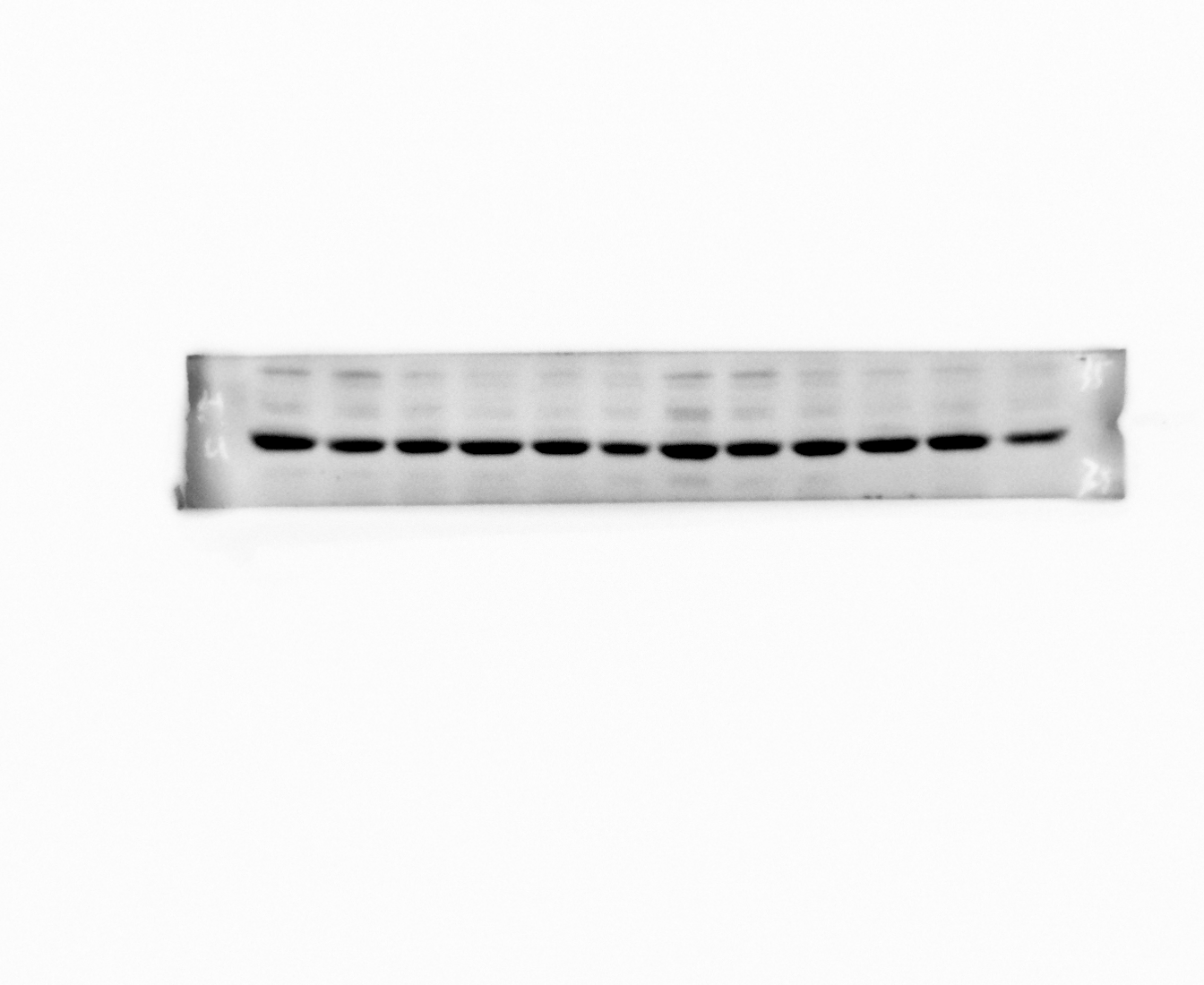

Supplement: Supplementary file 10 — Source data Fig. 4 [file 44321_2025_335_MOESM10_ESM.zip › Figure 4/Figure 4-F/western Ucp1.tif]

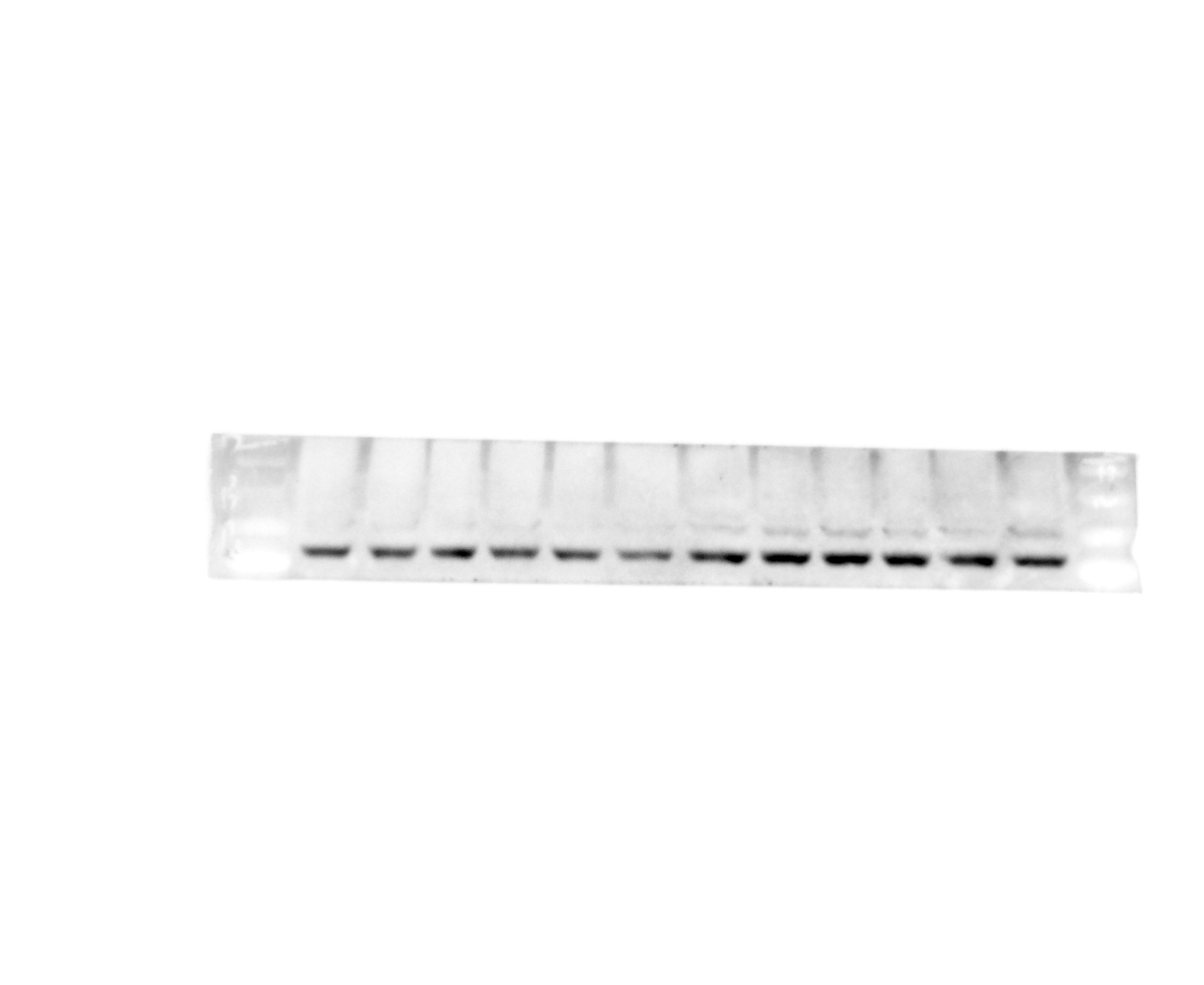

Supplement: Supplementary file 10 — Source data Fig. 4 [file 44321_2025_335_MOESM10_ESM.zip › Figure 4/Figure 4-H/western Pgc1╬▒.tif]

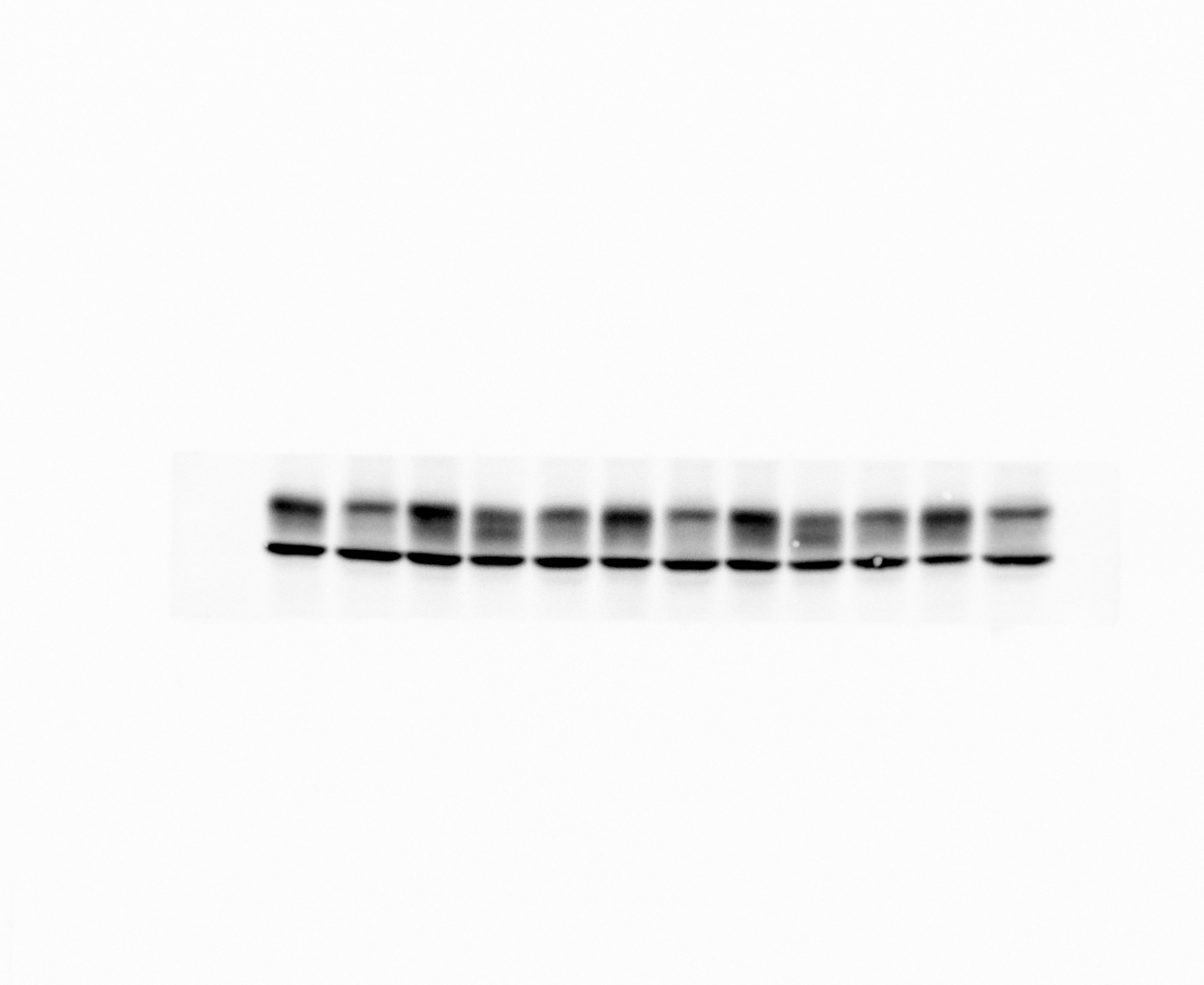

Supplement: Supplementary file 10 — Source data Fig. 4 [file 44321_2025_335_MOESM10_ESM.zip › Figure 4/Figure 4-H/western actin.tif]

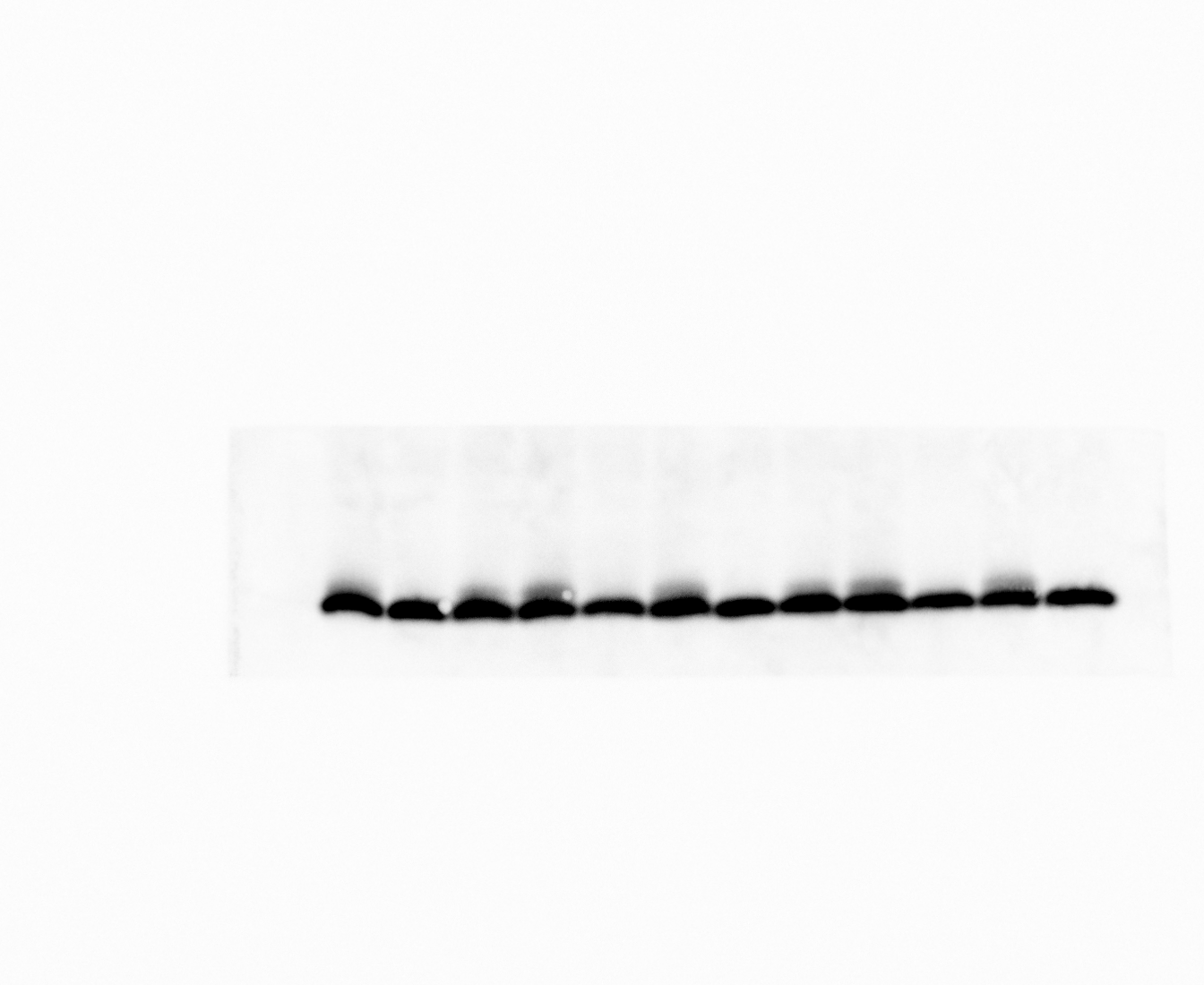

Supplement: Supplementary file 10 — Source data Fig. 4 [file 44321_2025_335_MOESM10_ESM.zip › Figure 4/Figure 4-H/western Fabp4.tif]

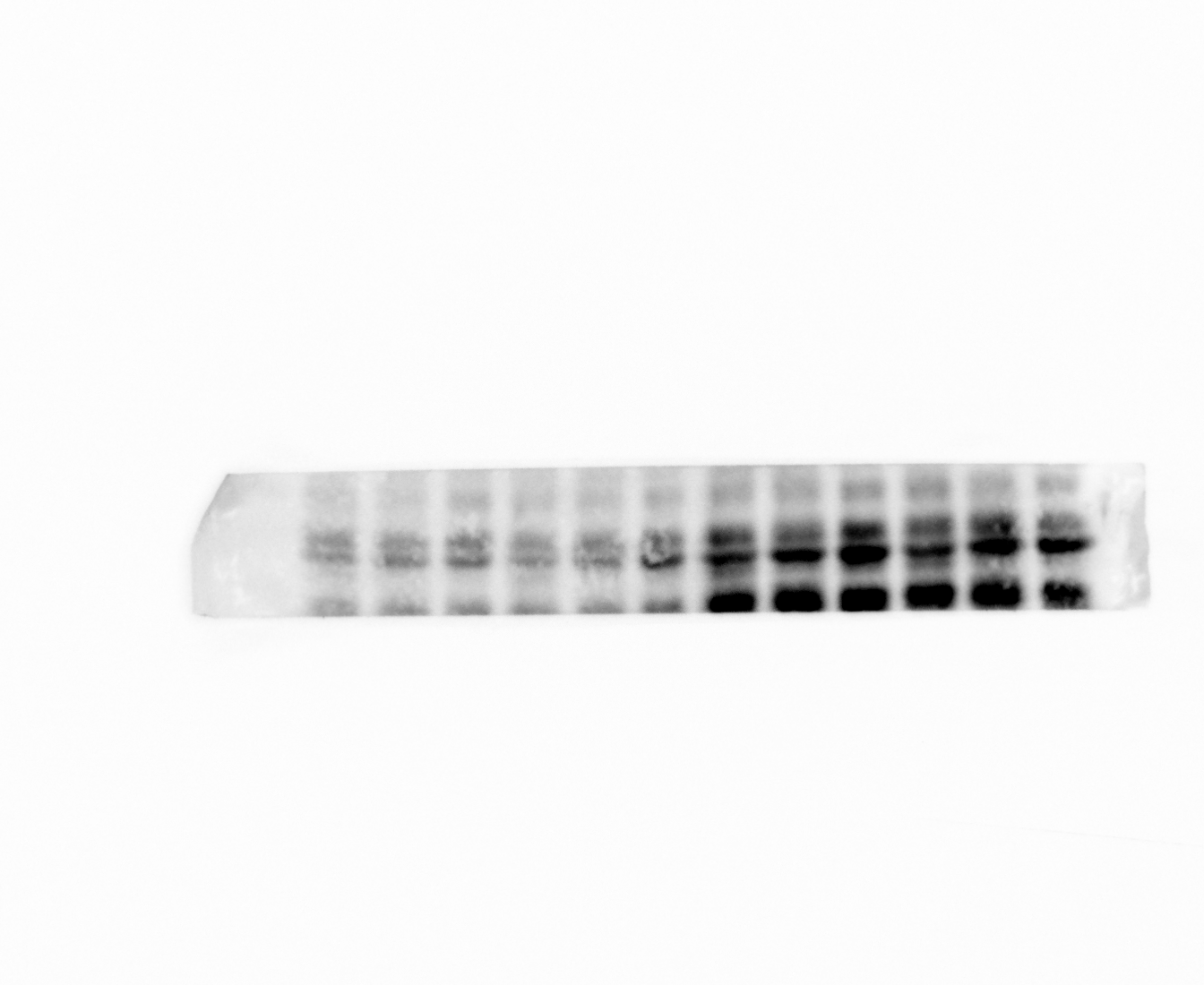

Supplement: Supplementary file 10 — Source data Fig. 4 [file 44321_2025_335_MOESM10_ESM.zip › Figure 4/Figure 4-H/western Ucp1.tif]

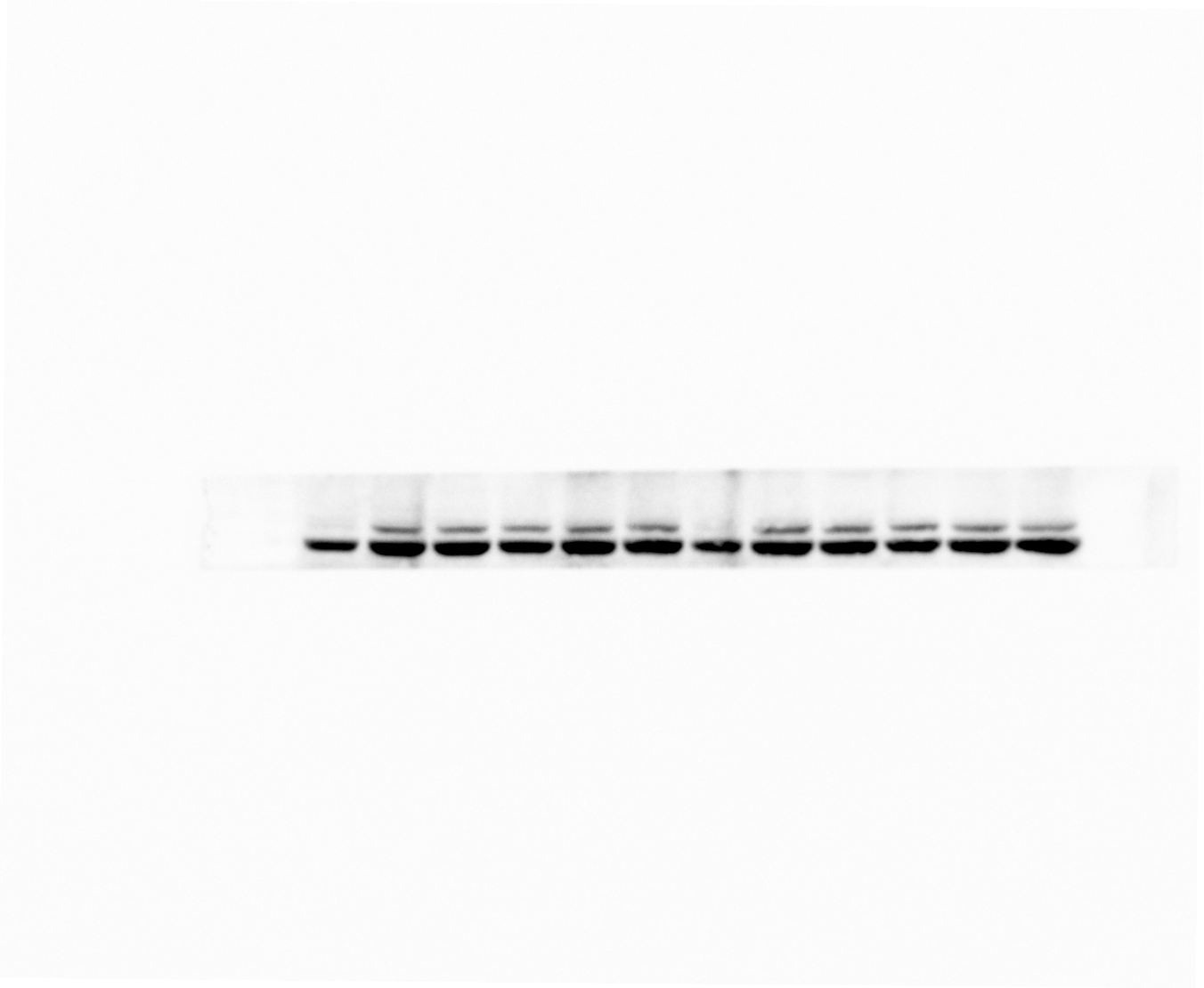

Supplement: Supplementary file 10 — Source data Fig. 4 [file 44321_2025_335_MOESM10_ESM.zip › Figure 4/Figure 4-E/western Pgc1╬▒.tif]

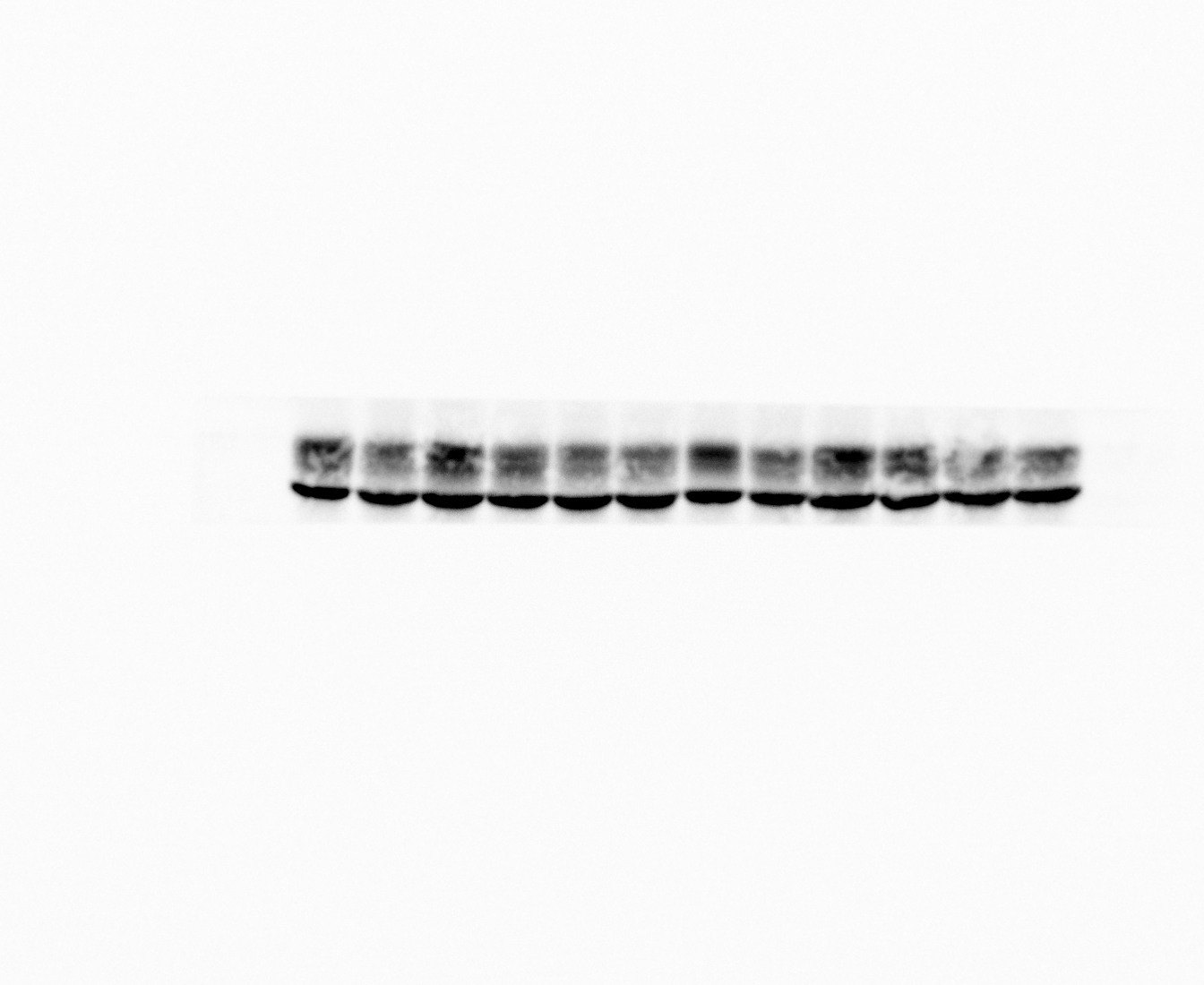

Supplement: Supplementary file 10 — Source data Fig. 4 [file 44321_2025_335_MOESM10_ESM.zip › Figure 4/Figure 4-E/western actin.tif]

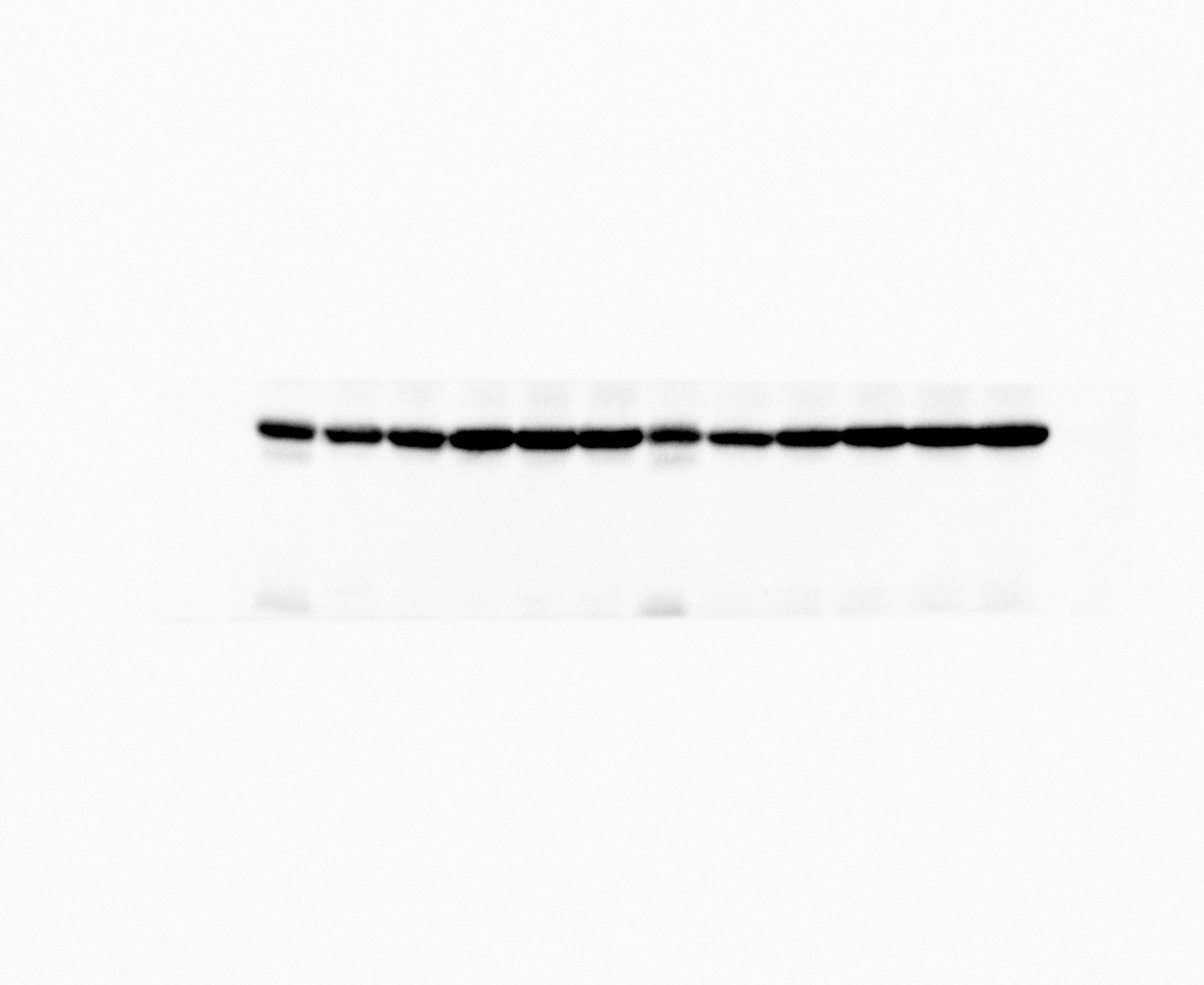

Supplement: Supplementary file 10 — Source data Fig. 4 [file 44321_2025_335_MOESM10_ESM.zip › Figure 4/Figure 4-E/western Ucp1.tif]

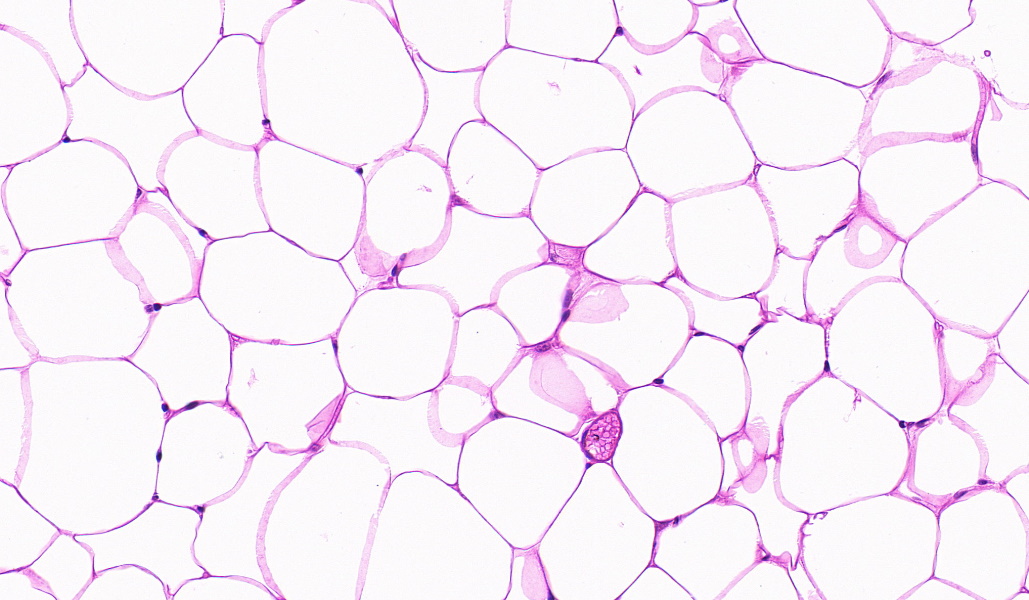

Supplement: Supplementary file 10 — Source data Fig. 4 [file 44321_2025_335_MOESM10_ESM.zip › Figure 4/Figure 4-A/HE staining/ISM- eWAT_20.0x.jpg]

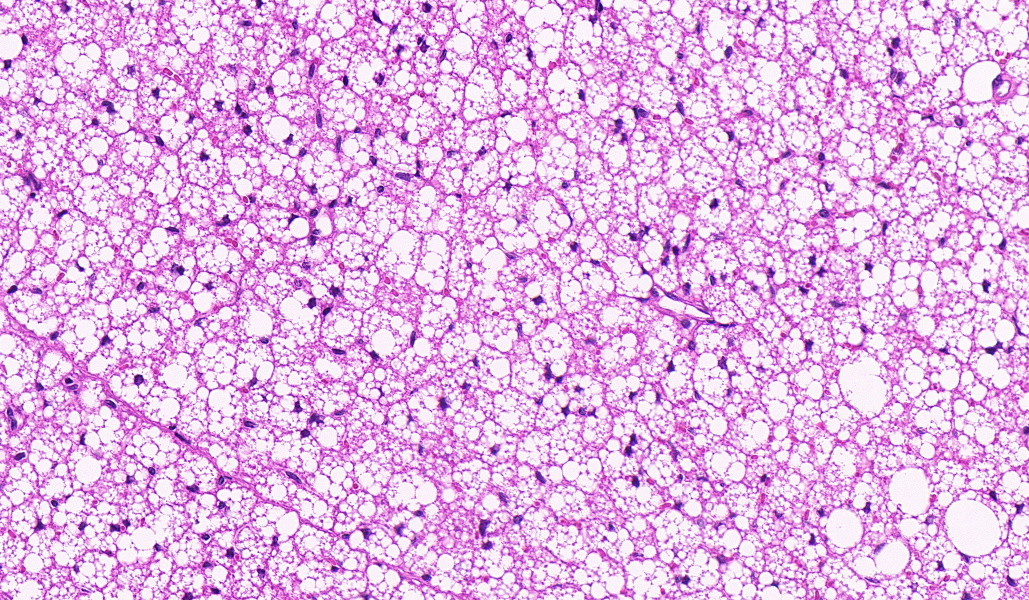

Supplement: Supplementary file 10 — Source data Fig. 4 [file 44321_2025_335_MOESM10_ESM.zip › Figure 4/Figure 4-A/HE staining/ISM- BAT _20.0x.jpg]

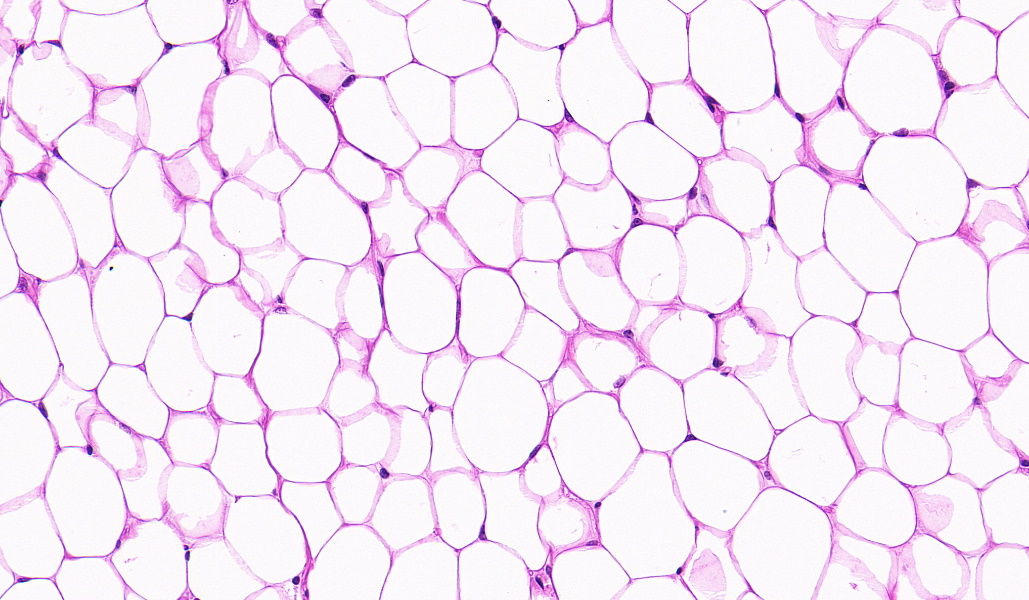

Supplement: Supplementary file 10 — Source data Fig. 4 [file 44321_2025_335_MOESM10_ESM.zip › Figure 4/Figure 4-A/HE staining/ISM iWAT _20.0x.jpg]

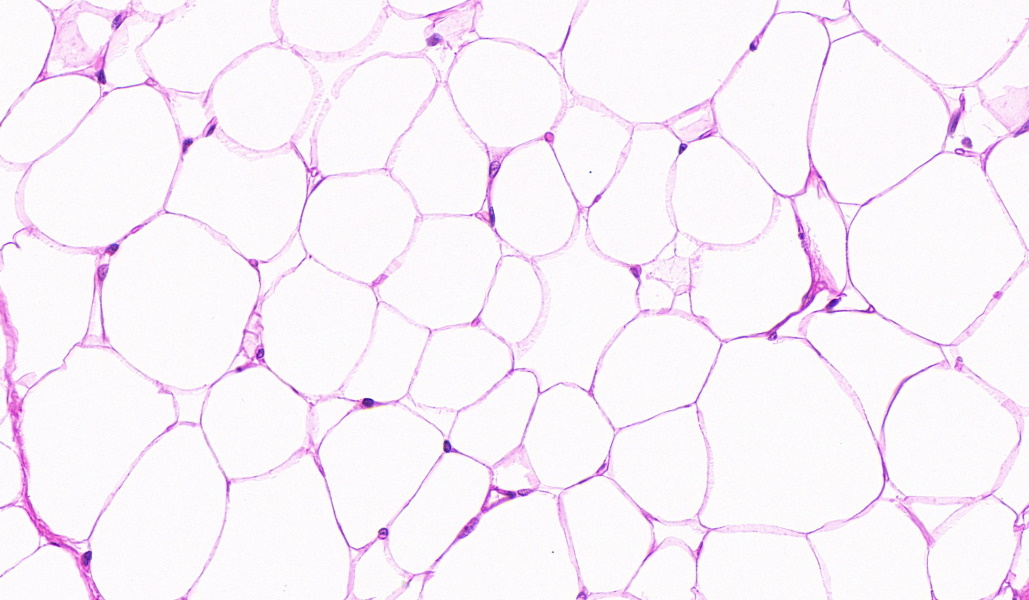

Supplement: Supplementary file 10 — Source data Fig. 4 [file 44321_2025_335_MOESM10_ESM.zip › Figure 4/Figure 4-A/HE staining/CON-iWAT _20.0x.jpg]

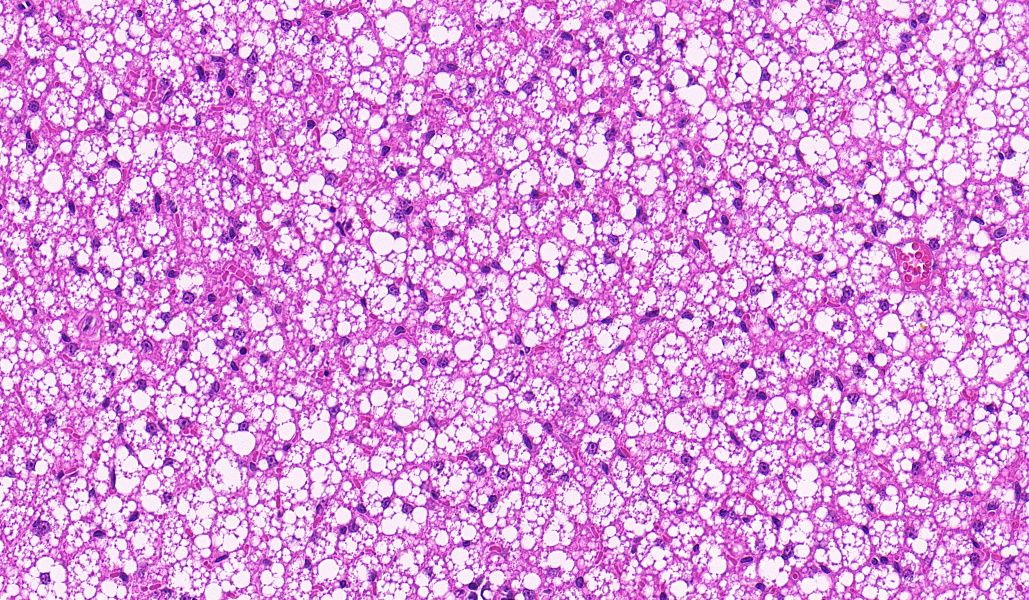

Supplement: Supplementary file 10 — Source data Fig. 4 [file 44321_2025_335_MOESM10_ESM.zip › Figure 4/Figure 4-A/HE staining/CON- BAT _20.0x.jpg]

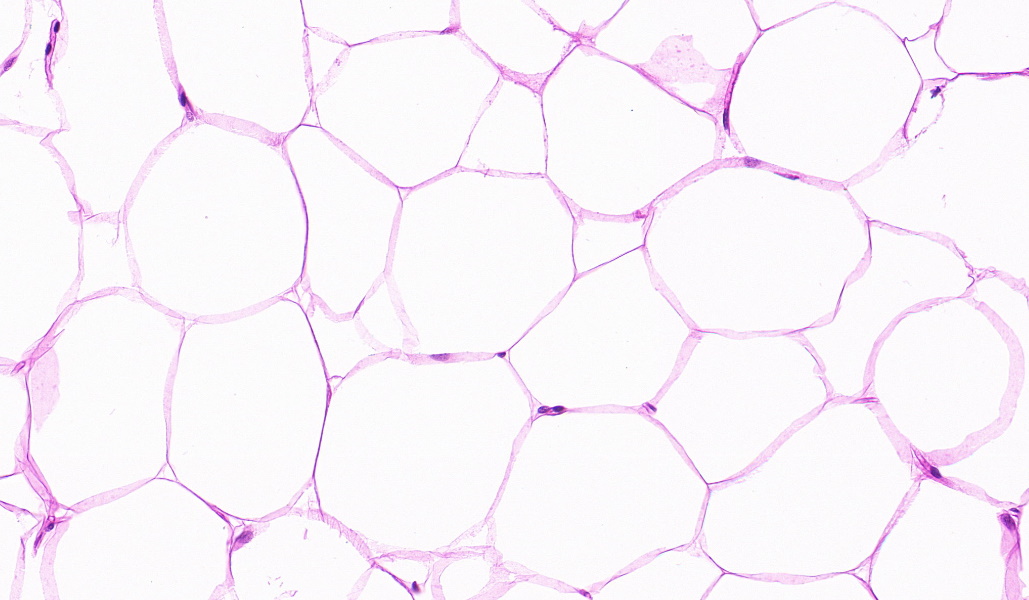

Supplement: Supplementary file 10 — Source data Fig. 4 [file 44321_2025_335_MOESM10_ESM.zip › Figure 4/Figure 4-A/HE staining/CON- eWAT_20.0x.jpg]

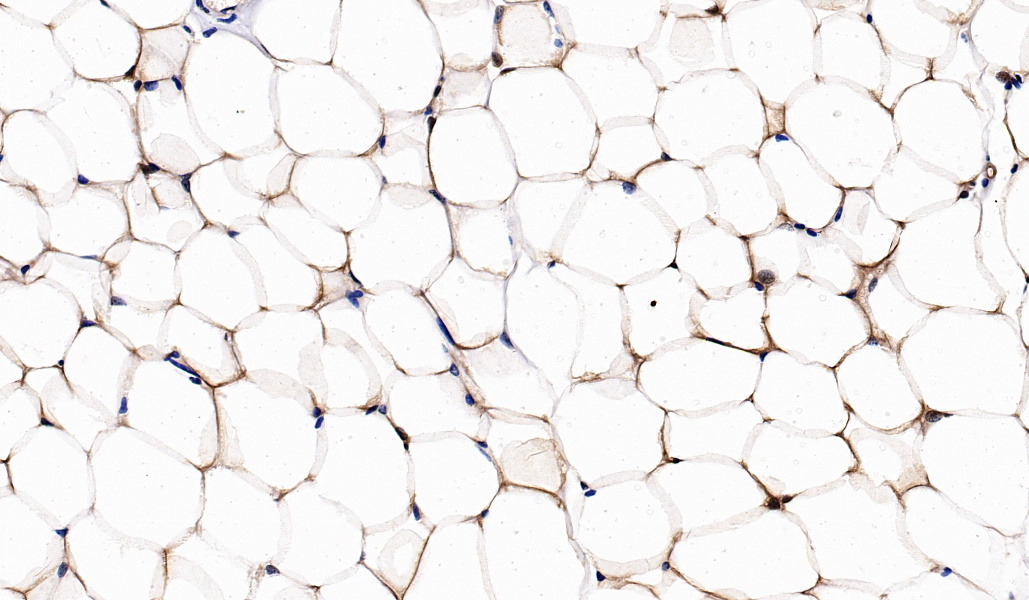

Supplement: Supplementary file 10 — Source data Fig. 4 [file 44321_2025_335_MOESM10_ESM.zip › Figure 4/Figure 4-I/immunohistochemical staining/HFD-ISM-iWAT ucp1_20.0x.jpg]

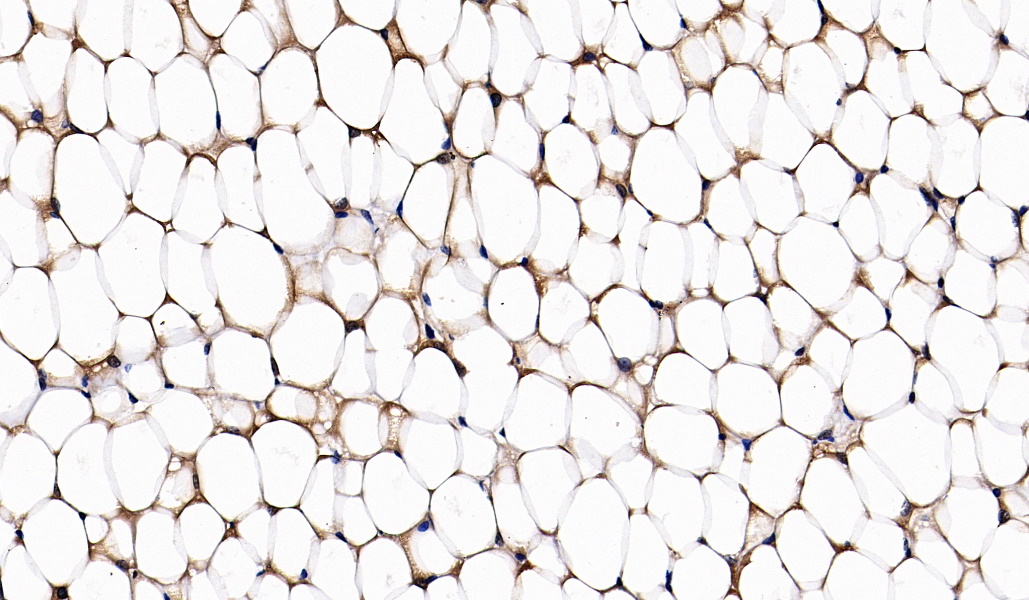

Supplement: Supplementary file 10 — Source data Fig. 4 [file 44321_2025_335_MOESM10_ESM.zip › Figure 4/Figure 4-I/immunohistochemical staining/SCD-ISM-iWAT ucp1_20.0x.jpg]

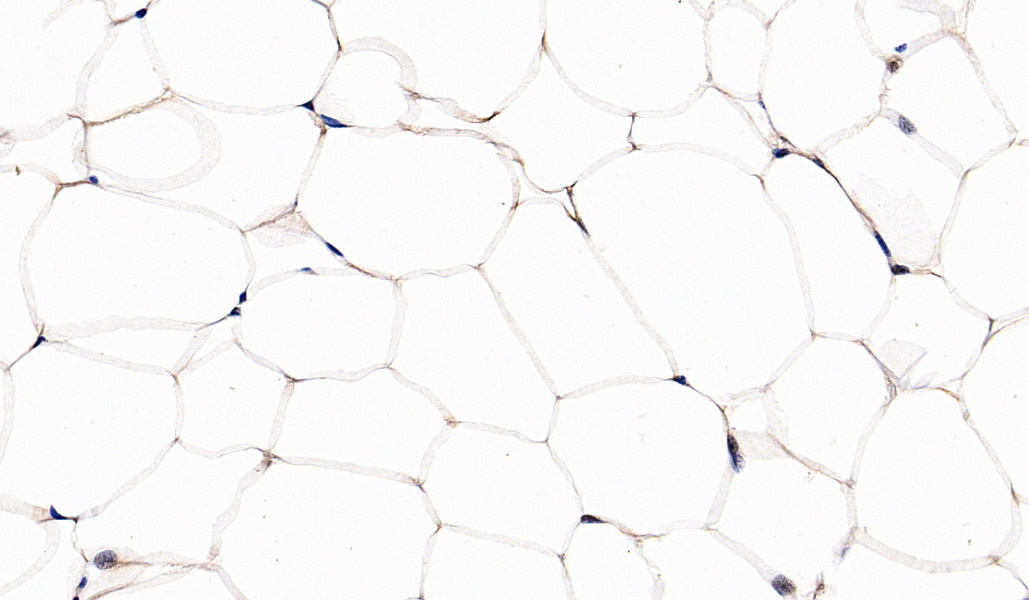

Supplement: Supplementary file 10 — Source data Fig. 4 [file 44321_2025_335_MOESM10_ESM.zip › Figure 4/Figure 4-I/immunohistochemical staining/HFD-CON-iWAT ucp1_20.0x.jpg]

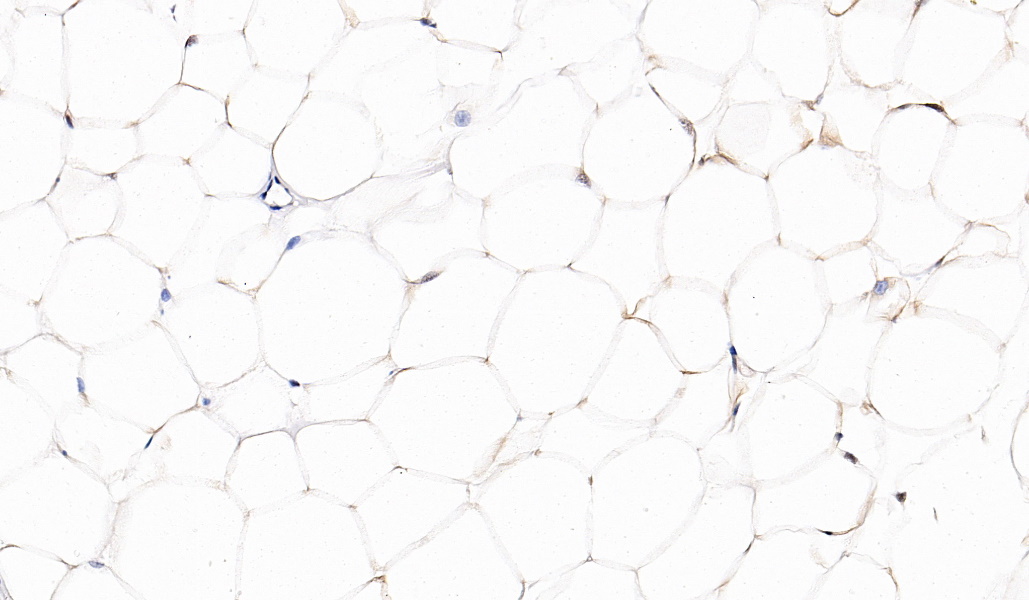

Supplement: Supplementary file 10 — Source data Fig. 4 [file 44321_2025_335_MOESM10_ESM.zip › Figure 4/Figure 4-I/immunohistochemical staining/SCD-CON- iWAT ucp1_20.0x.jpg]

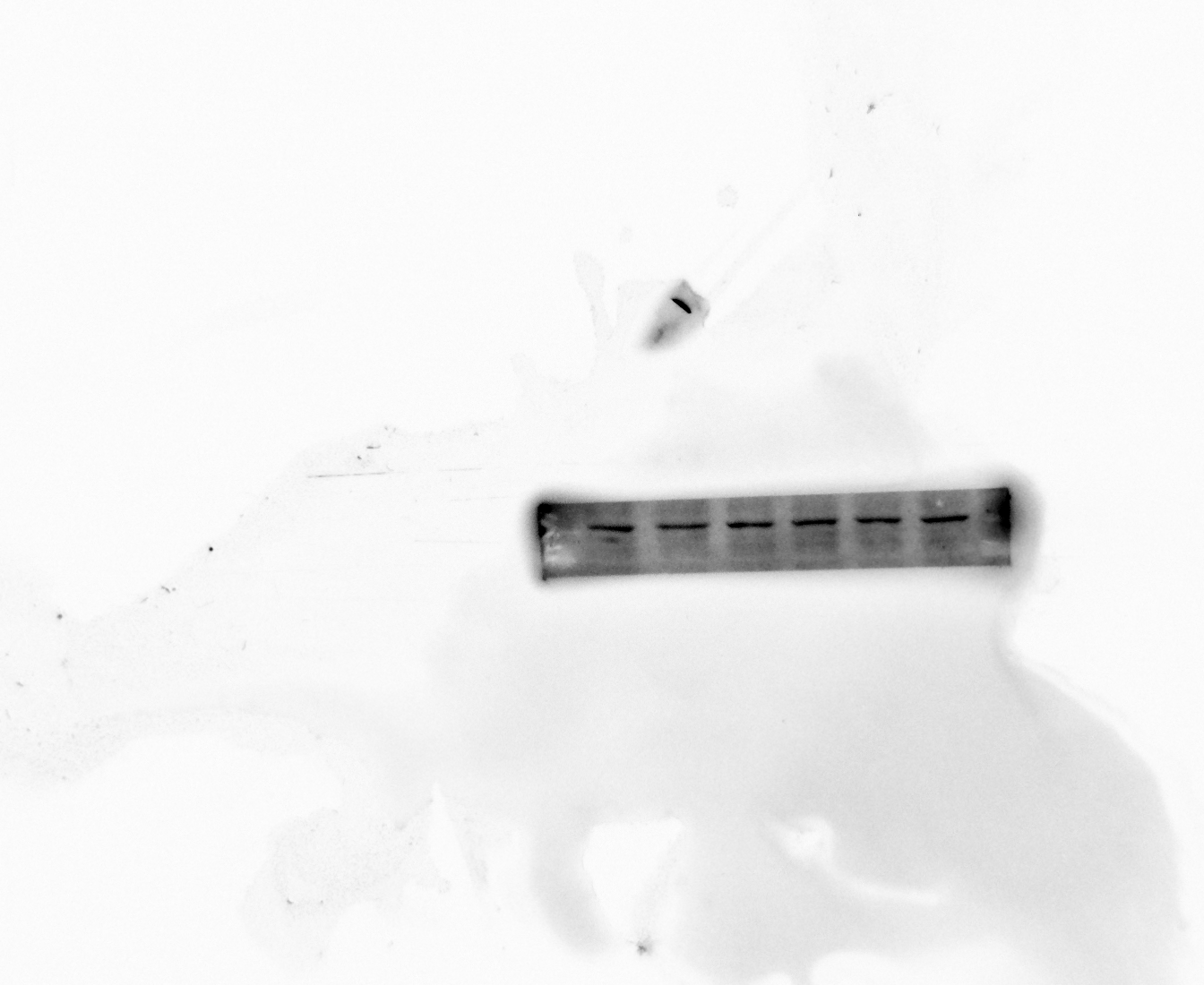

Supplement: Supplementary file 11 — Source data Fig. 5 [file 44321_2025_335_MOESM11_ESM.zip › Figure 5/Figure 5-C/western Pi3k.tif]

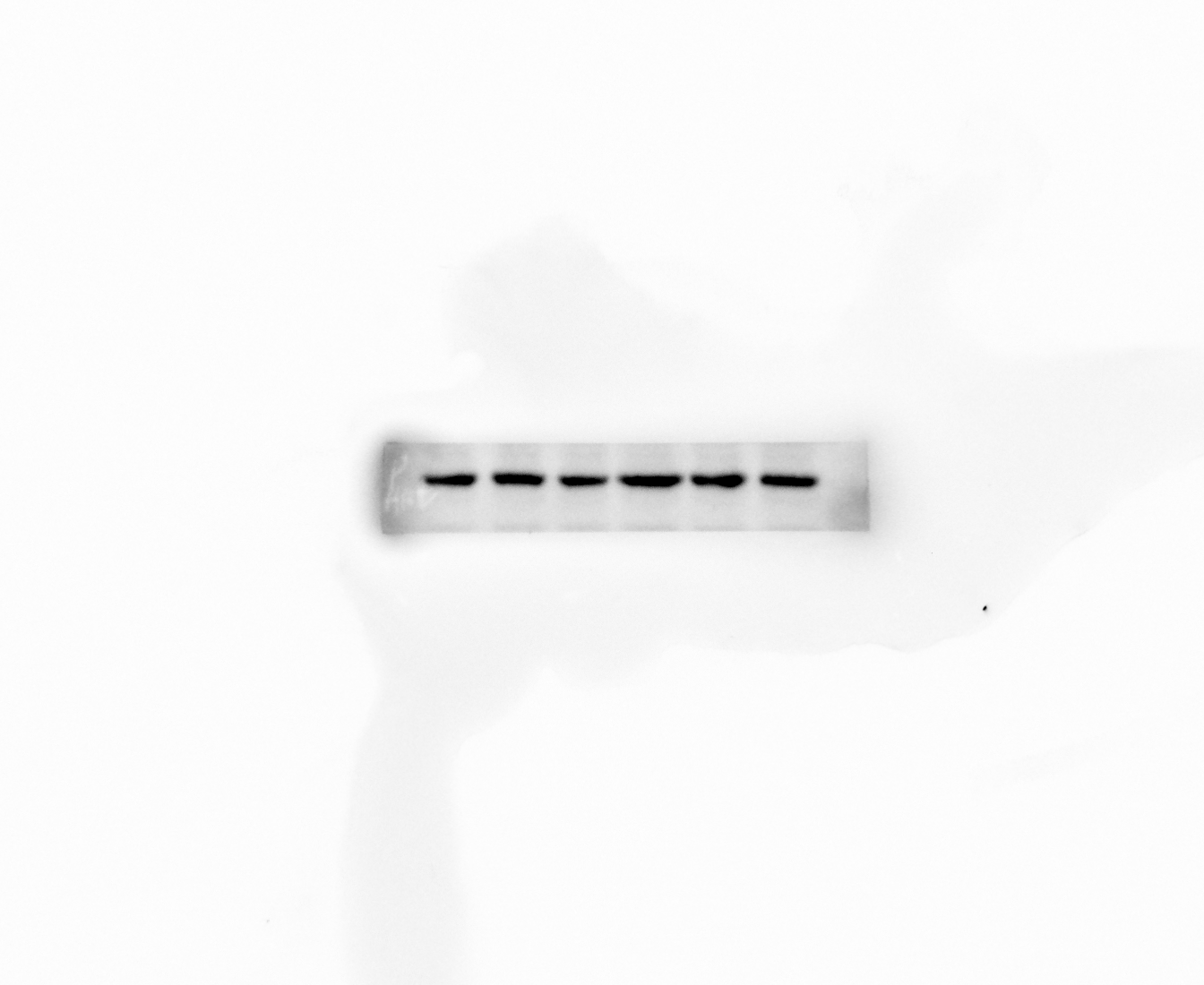

Supplement: Supplementary file 11 — Source data Fig. 5 [file 44321_2025_335_MOESM11_ESM.zip › Figure 5/Figure 5-C/western p-Akt.tif]

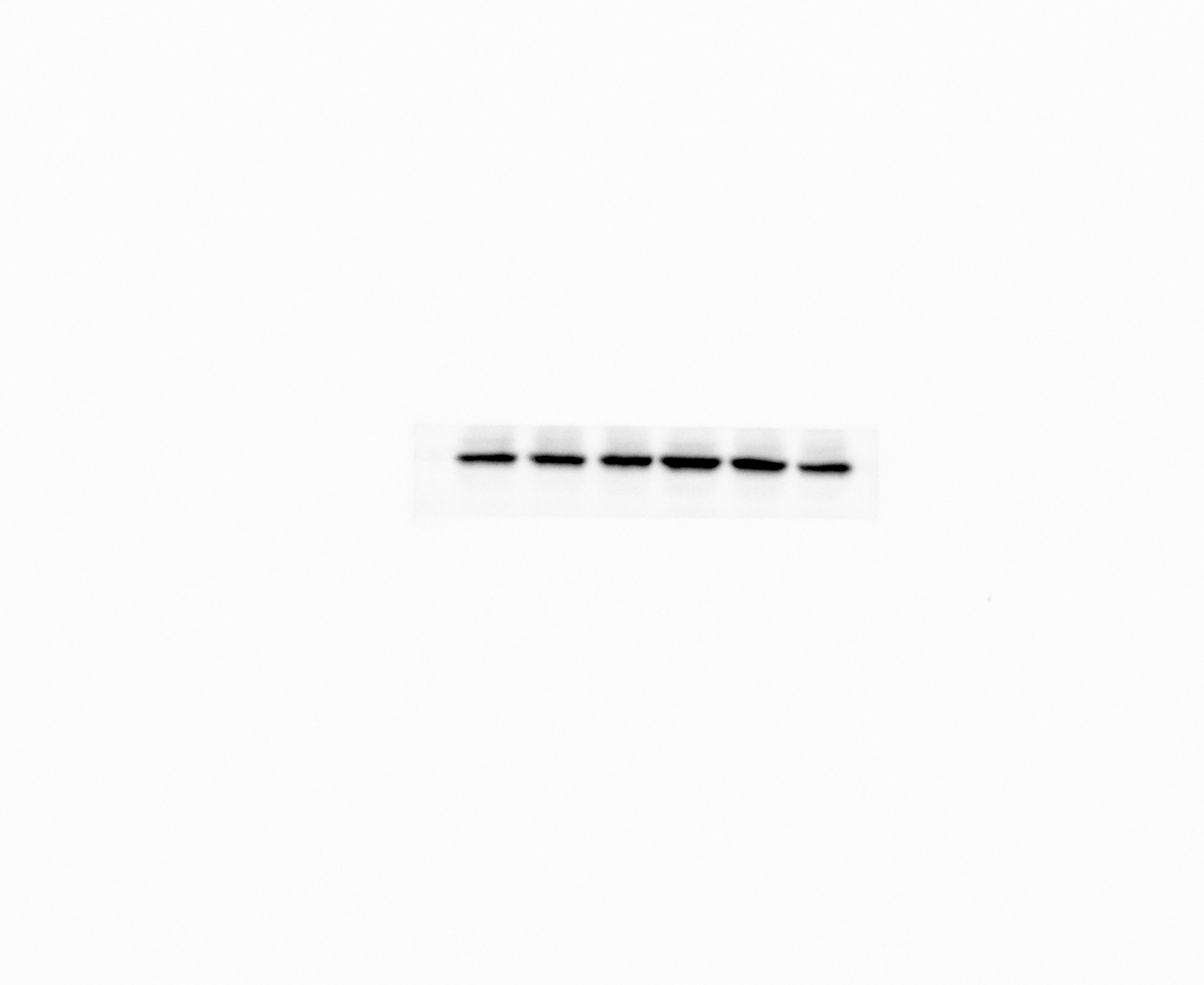

Supplement: Supplementary file 11 — Source data Fig. 5 [file 44321_2025_335_MOESM11_ESM.zip › Figure 5/Figure 5-C/western Akt.tif]

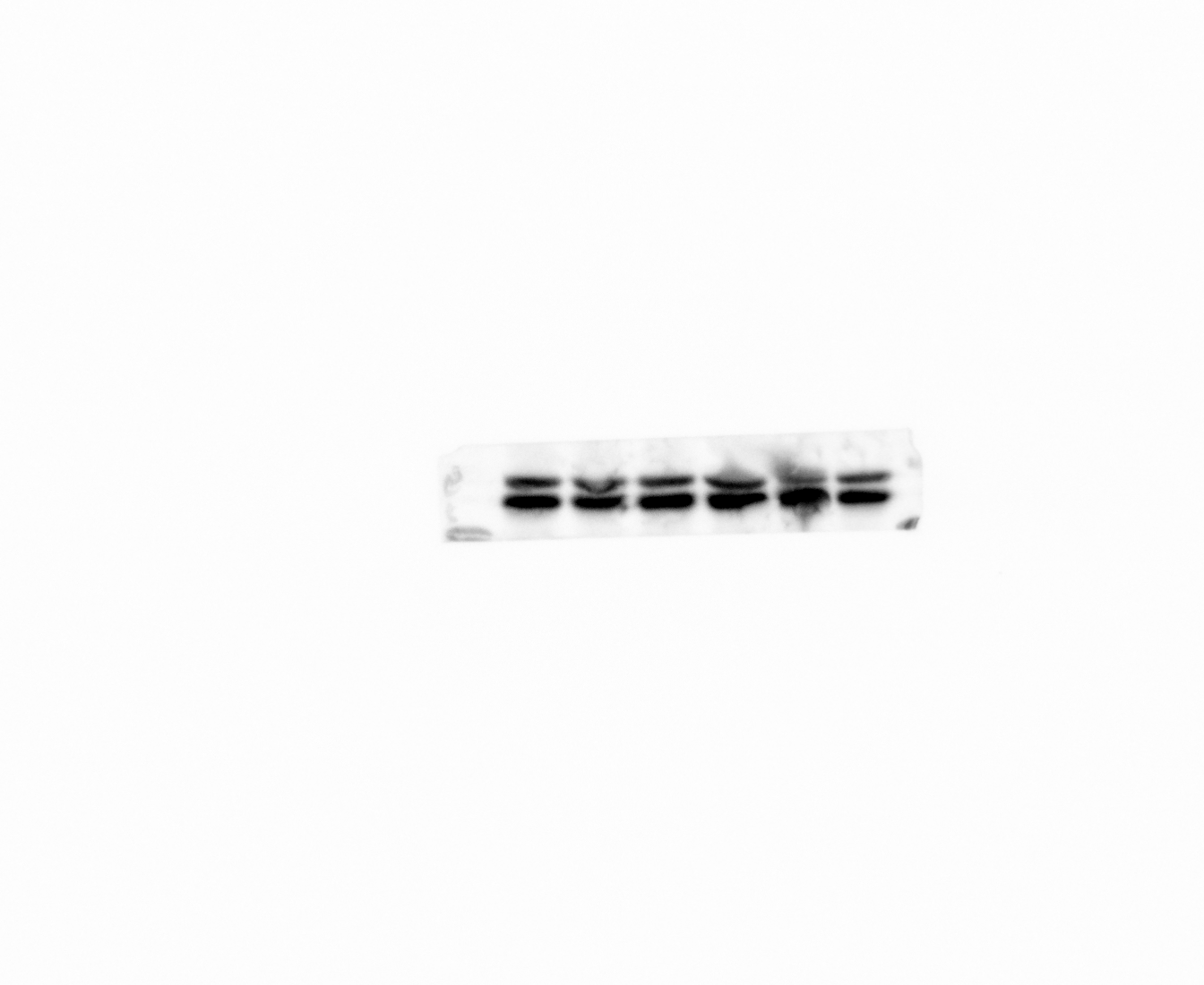

Supplement: Supplementary file 11 — Source data Fig. 5 [file 44321_2025_335_MOESM11_ESM.zip › Figure 5/Figure 5-C/western Erk.tif]

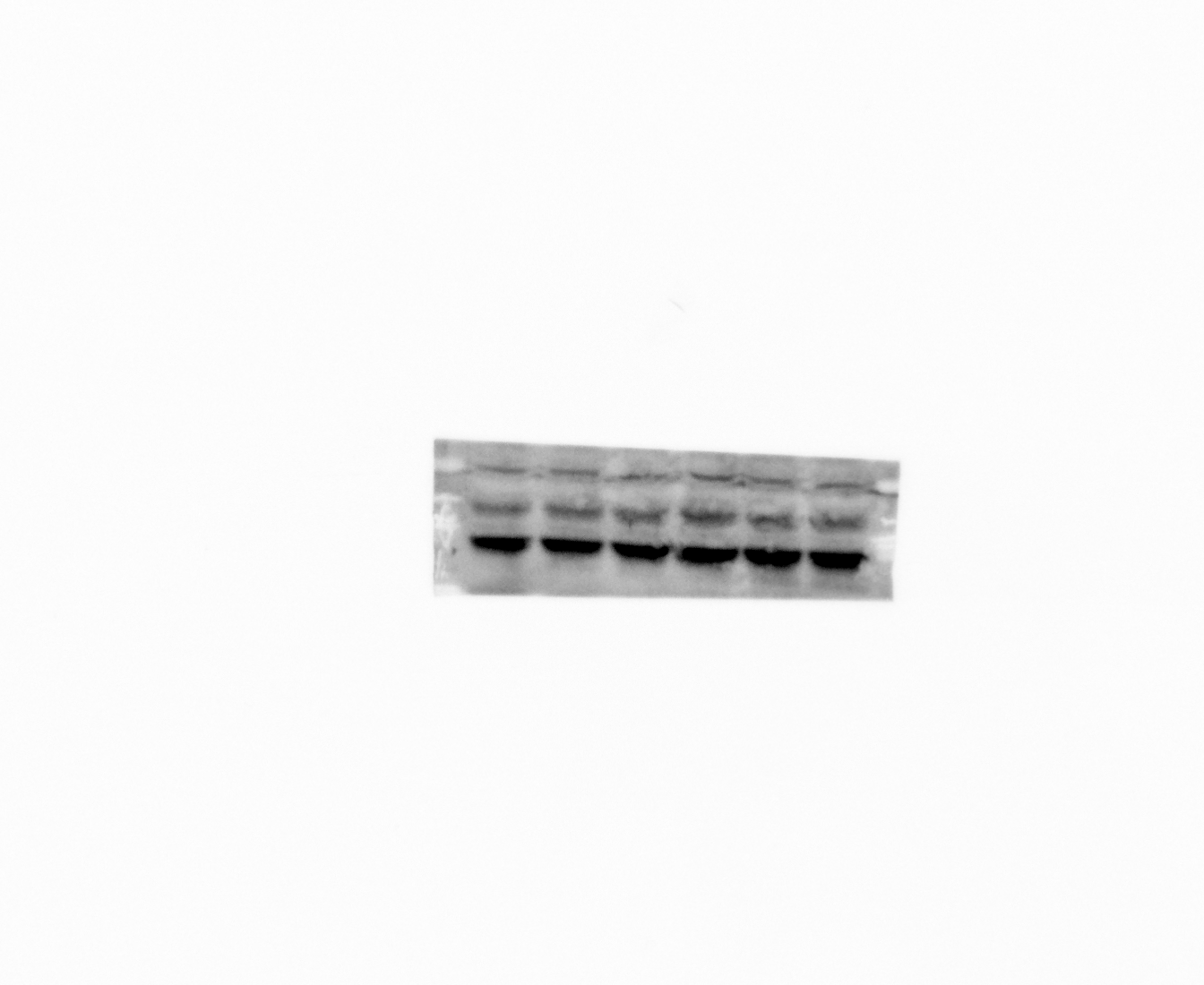

Supplement: Supplementary file 11 — Source data Fig. 5 [file 44321_2025_335_MOESM11_ESM.zip › Figure 5/Figure 5-C/western JNK.tif]

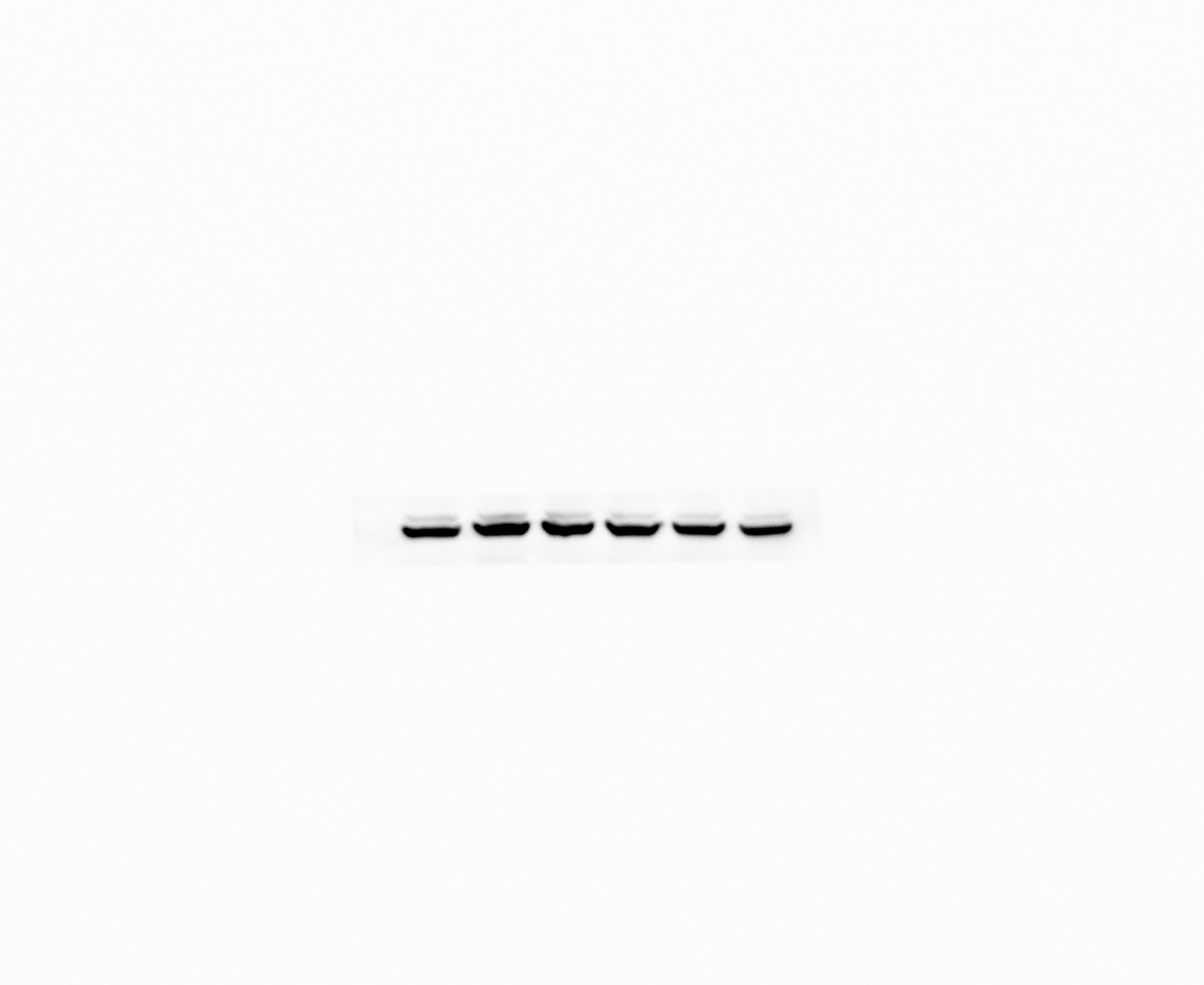

Supplement: Supplementary file 11 — Source data Fig. 5 [file 44321_2025_335_MOESM11_ESM.zip › Figure 5/Figure 5-C/western Hsp90.tif]

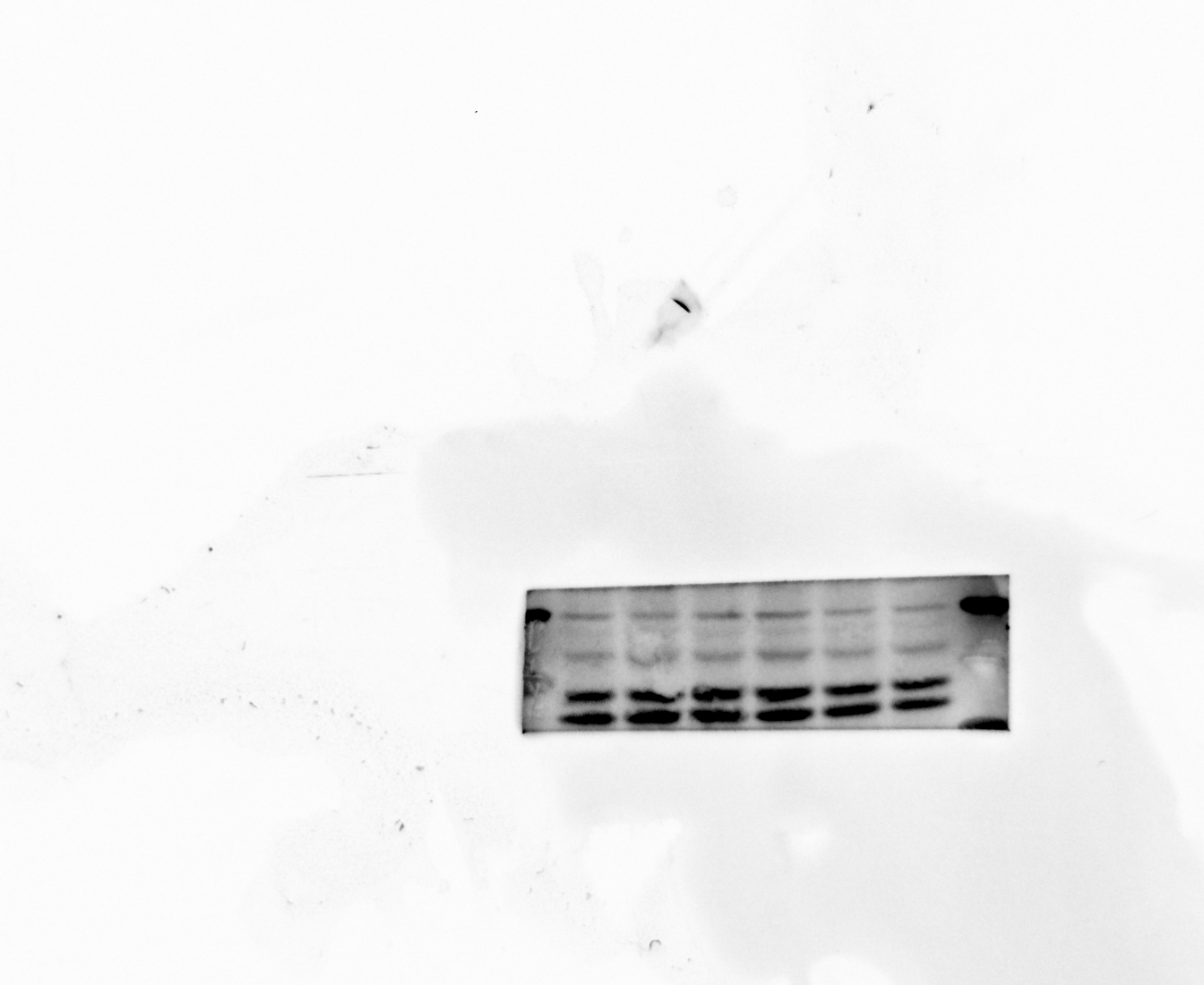

Supplement: Supplementary file 11 — Source data Fig. 5 [file 44321_2025_335_MOESM11_ESM.zip › Figure 5/Figure 5-C/western p-JNK.tif]

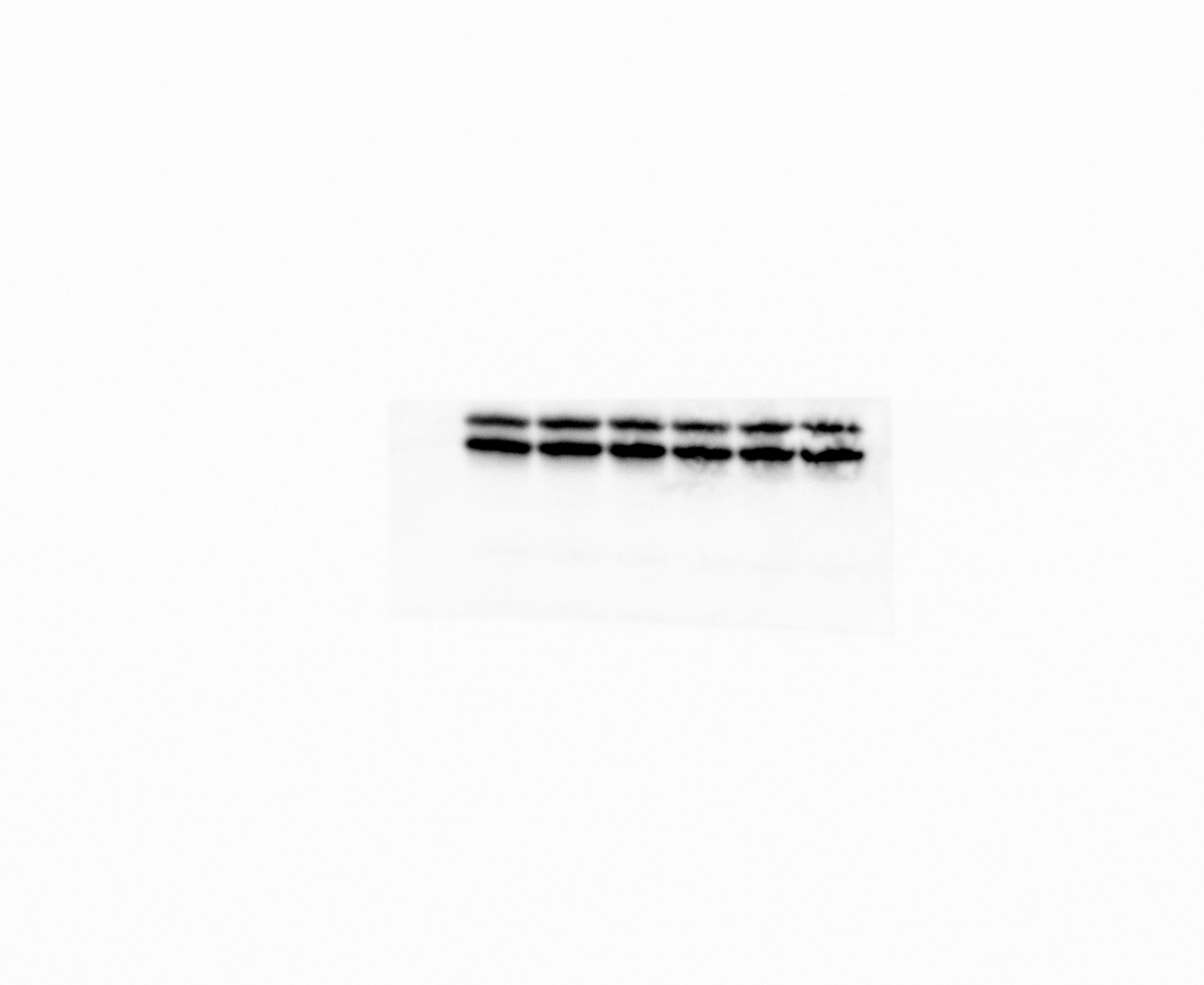

Supplement: Supplementary file 11 — Source data Fig. 5 [file 44321_2025_335_MOESM11_ESM.zip › Figure 5/Figure 5-C/western p-Erk.tif]

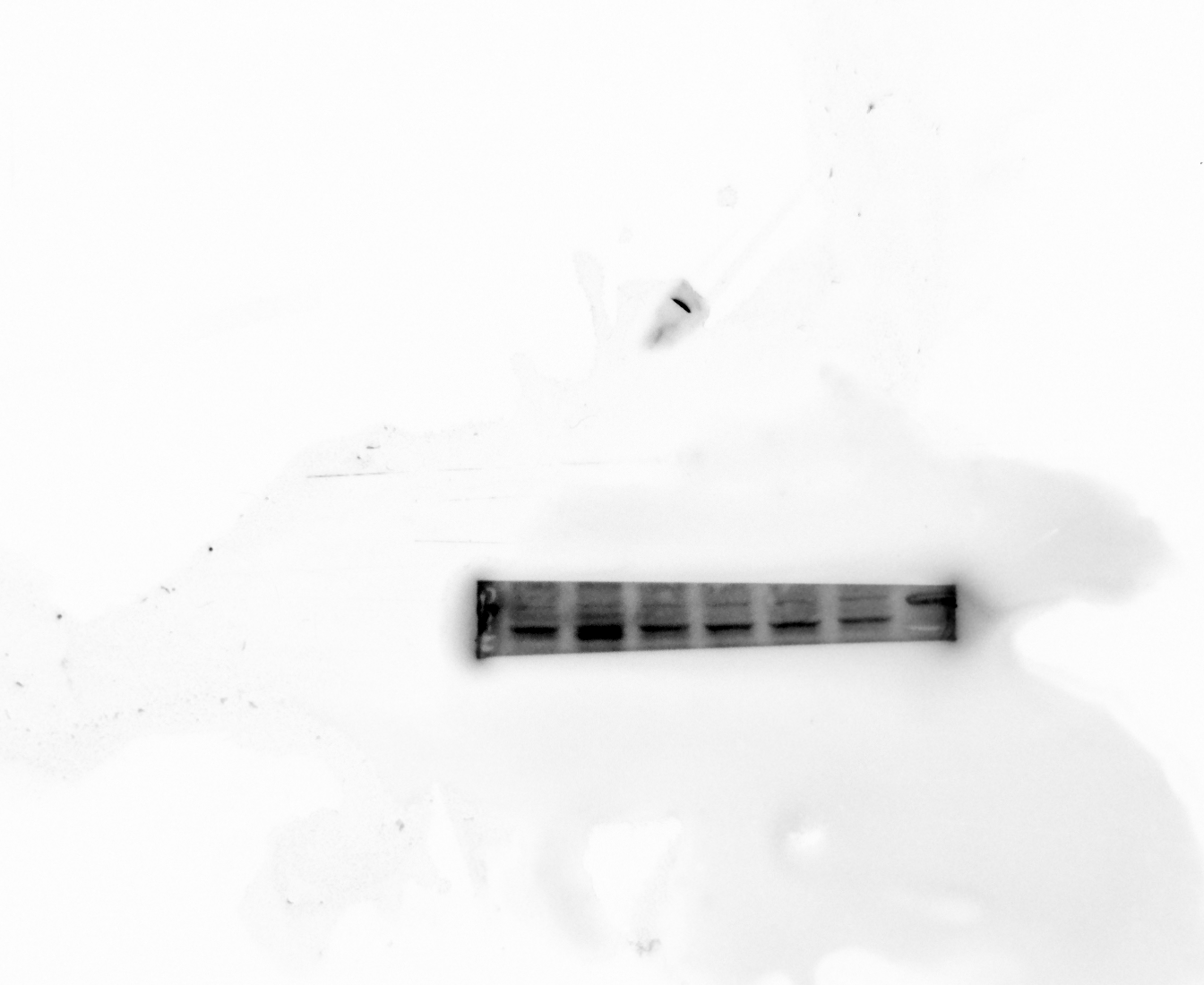

Supplement: Supplementary file 11 — Source data Fig. 5 [file 44321_2025_335_MOESM11_ESM.zip › Figure 5/Figure 5-C/western p-Pi3k.tif]

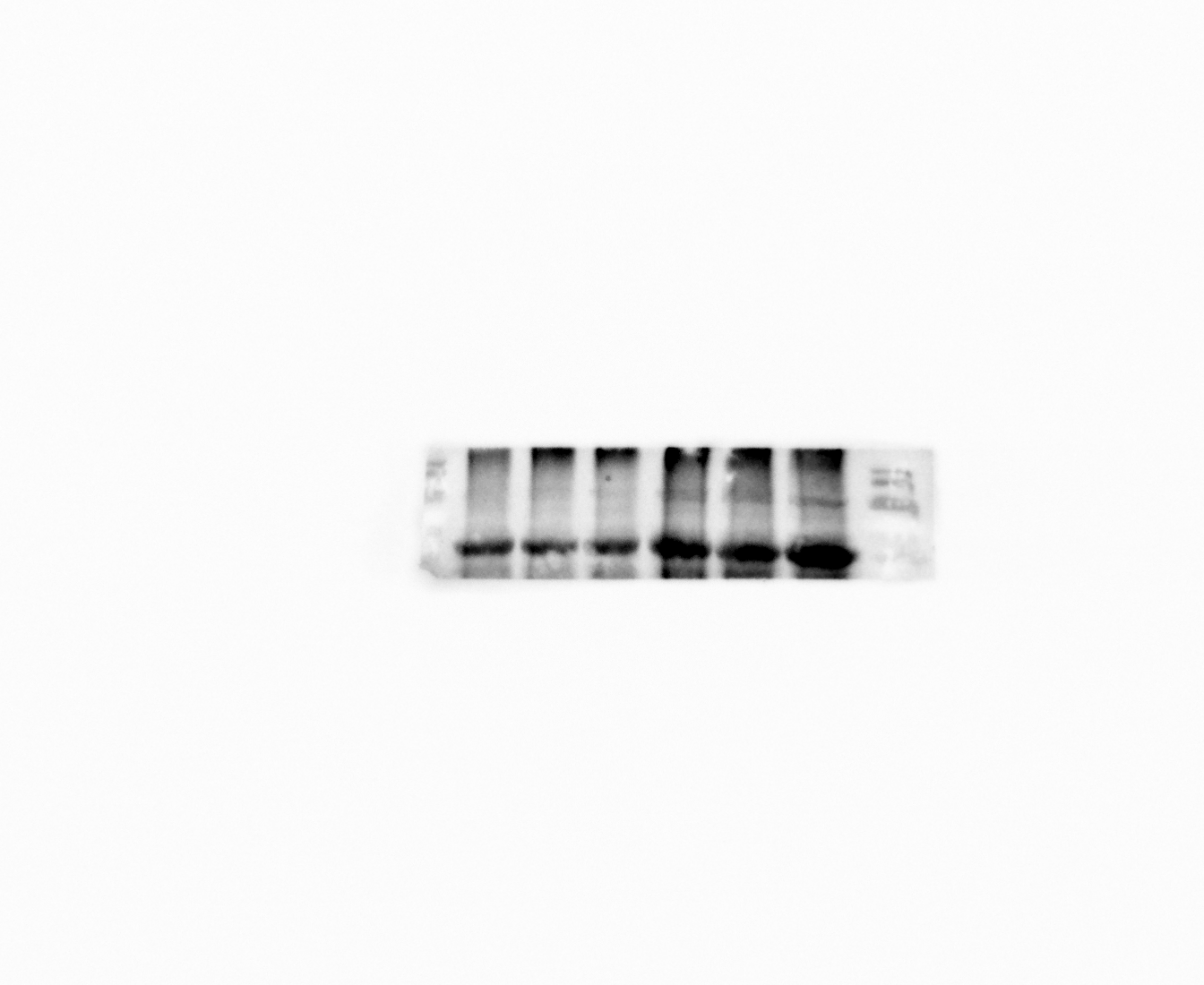

Supplement: Supplementary file 11 — Source data Fig. 5 [file 44321_2025_335_MOESM11_ESM.zip › Figure 5/Figure 5-F/western p-AMPK.tif]

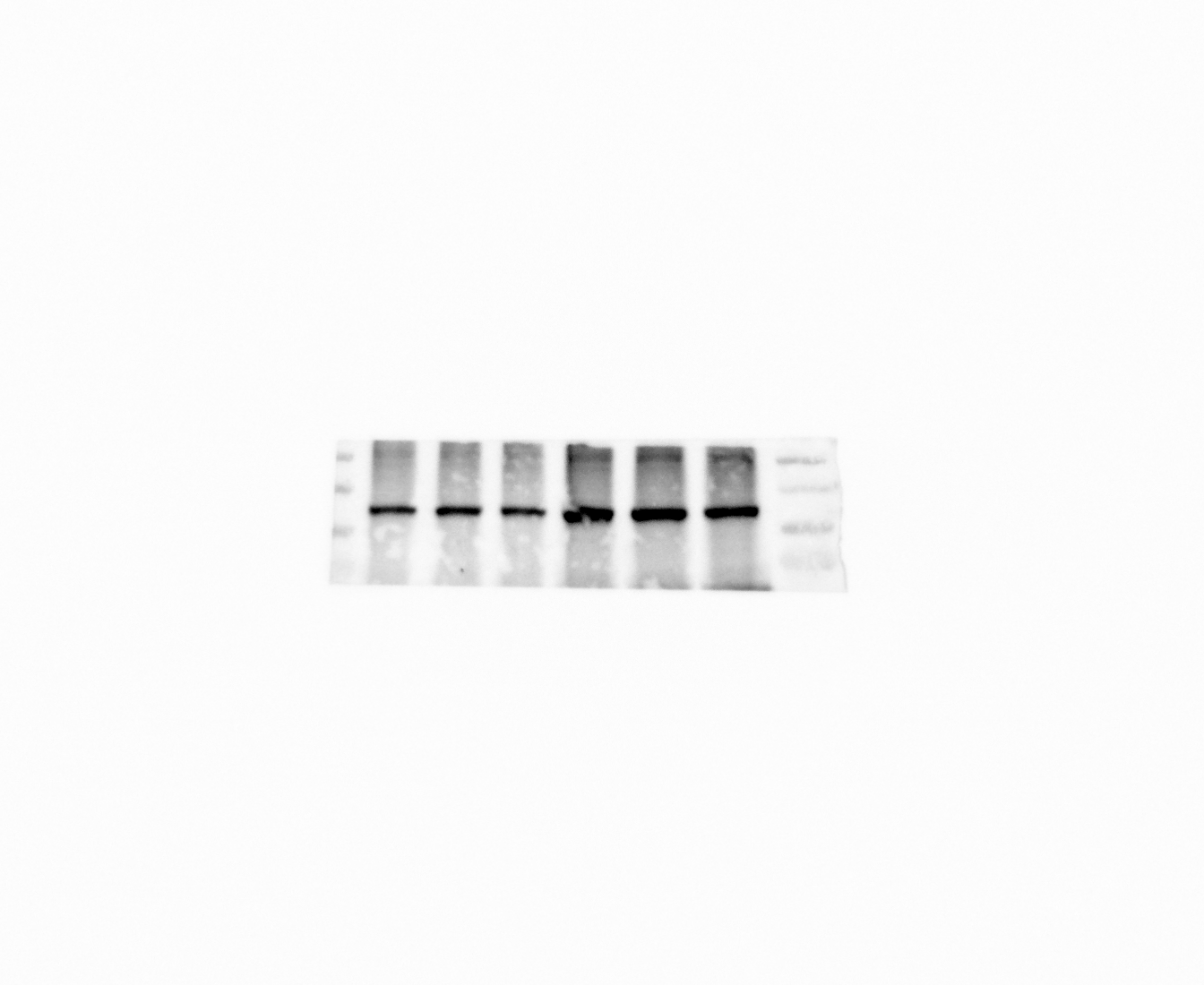

Supplement: Supplementary file 11 — Source data Fig. 5 [file 44321_2025_335_MOESM11_ESM.zip › Figure 5/Figure 5-F/western Pgc1╬▒.tif]

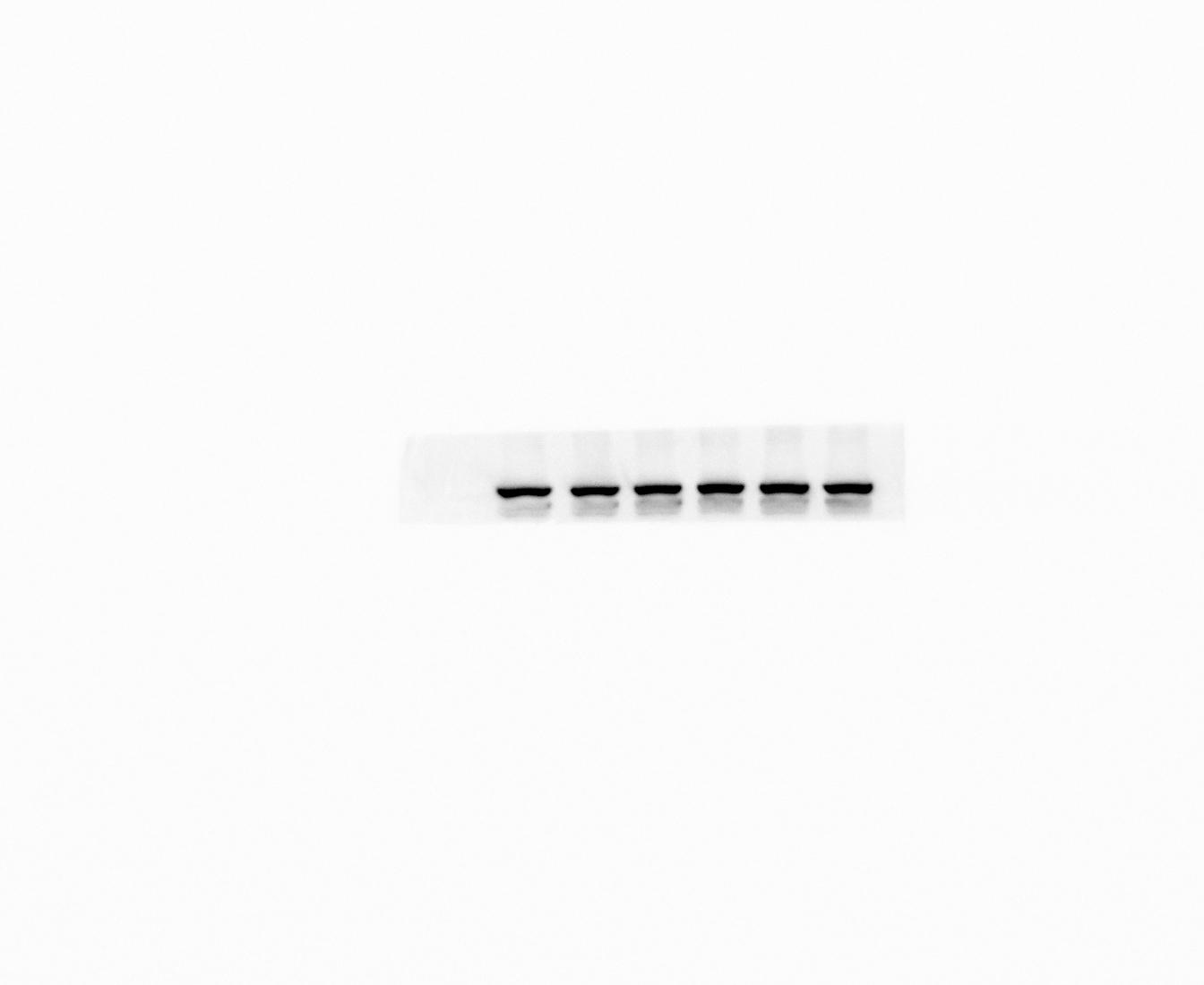

Supplement: Supplementary file 11 — Source data Fig. 5 [file 44321_2025_335_MOESM11_ESM.zip › Figure 5/Figure 5-F/western Hsp90.tif]

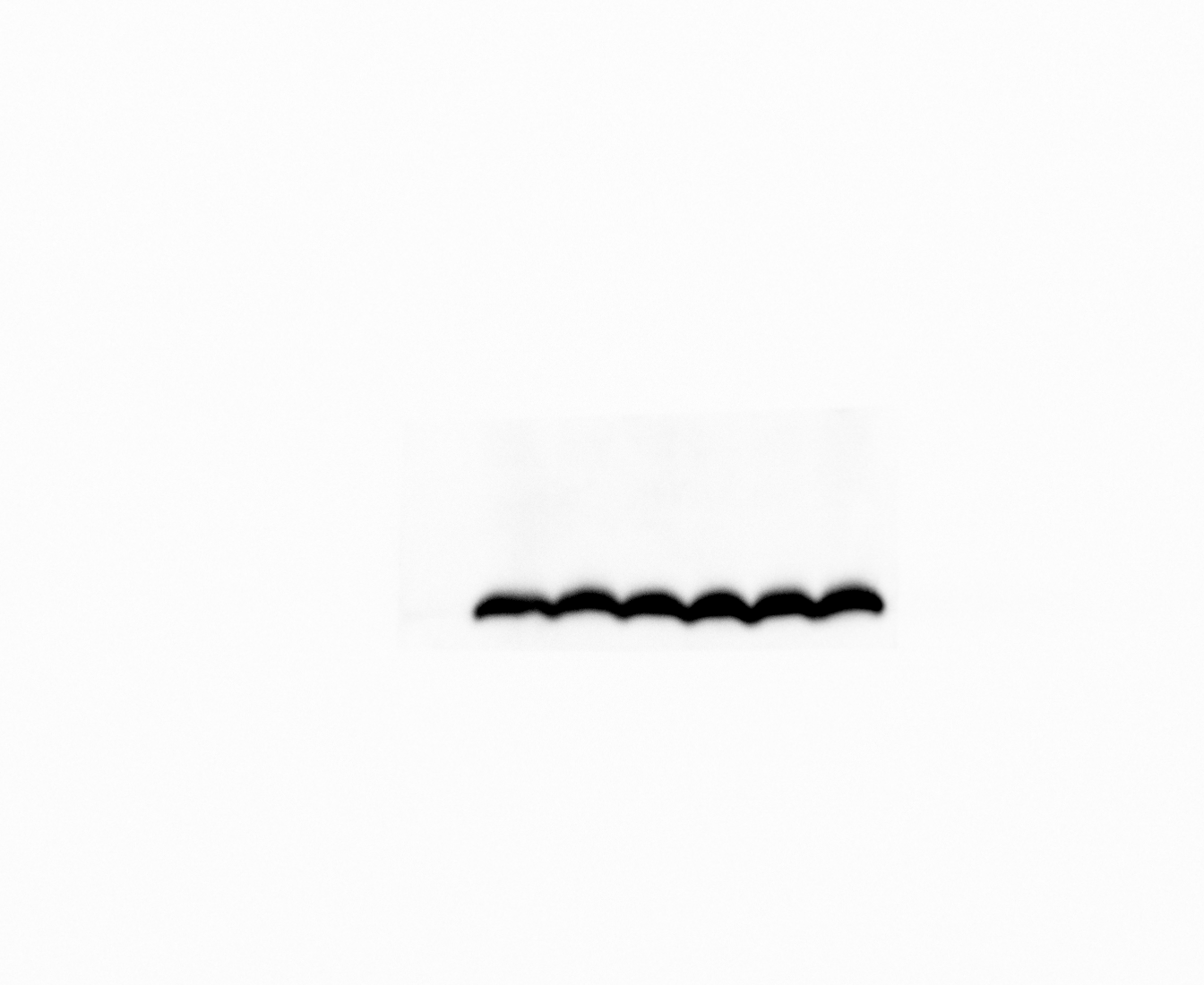

Supplement: Supplementary file 11 — Source data Fig. 5 [file 44321_2025_335_MOESM11_ESM.zip › Figure 5/Figure 5-F/western Fabp4.tif]

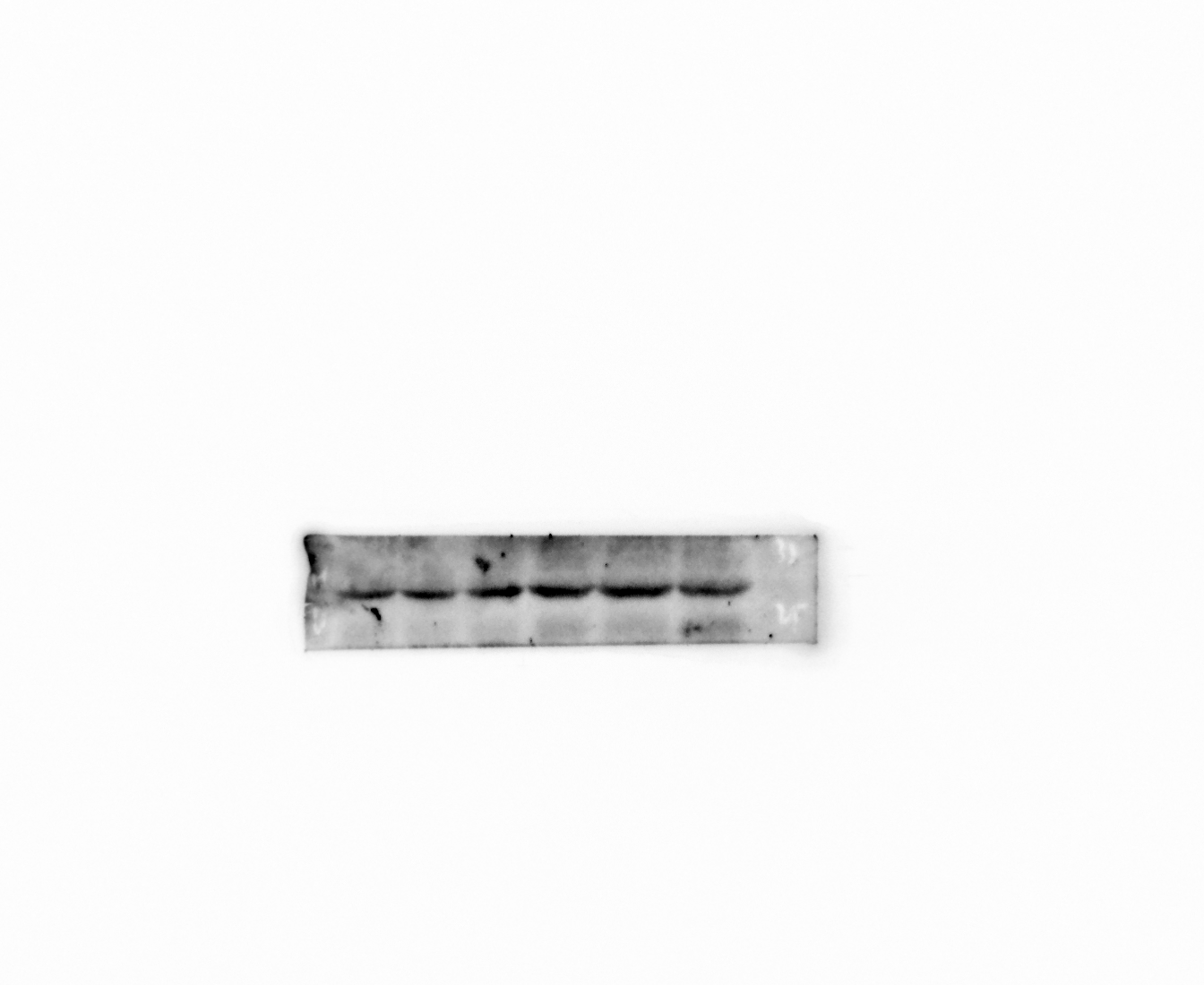

Supplement: Supplementary file 11 — Source data Fig. 5 [file 44321_2025_335_MOESM11_ESM.zip › Figure 5/Figure 5-F/western Ucp1.tif]

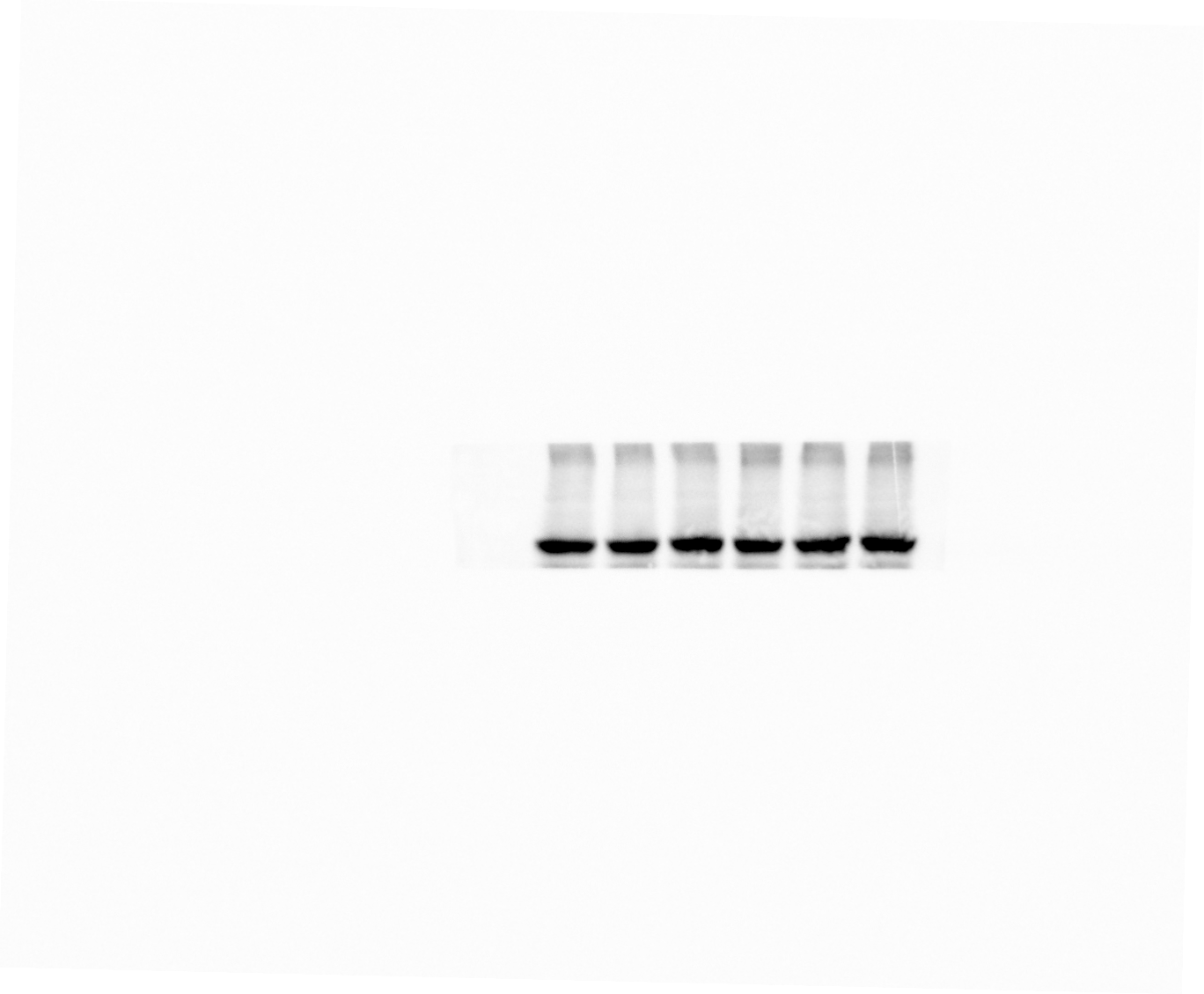

Supplement: Supplementary file 11 — Source data Fig. 5 [file 44321_2025_335_MOESM11_ESM.zip › Figure 5/Figure 5-F/western AMPK.tif]

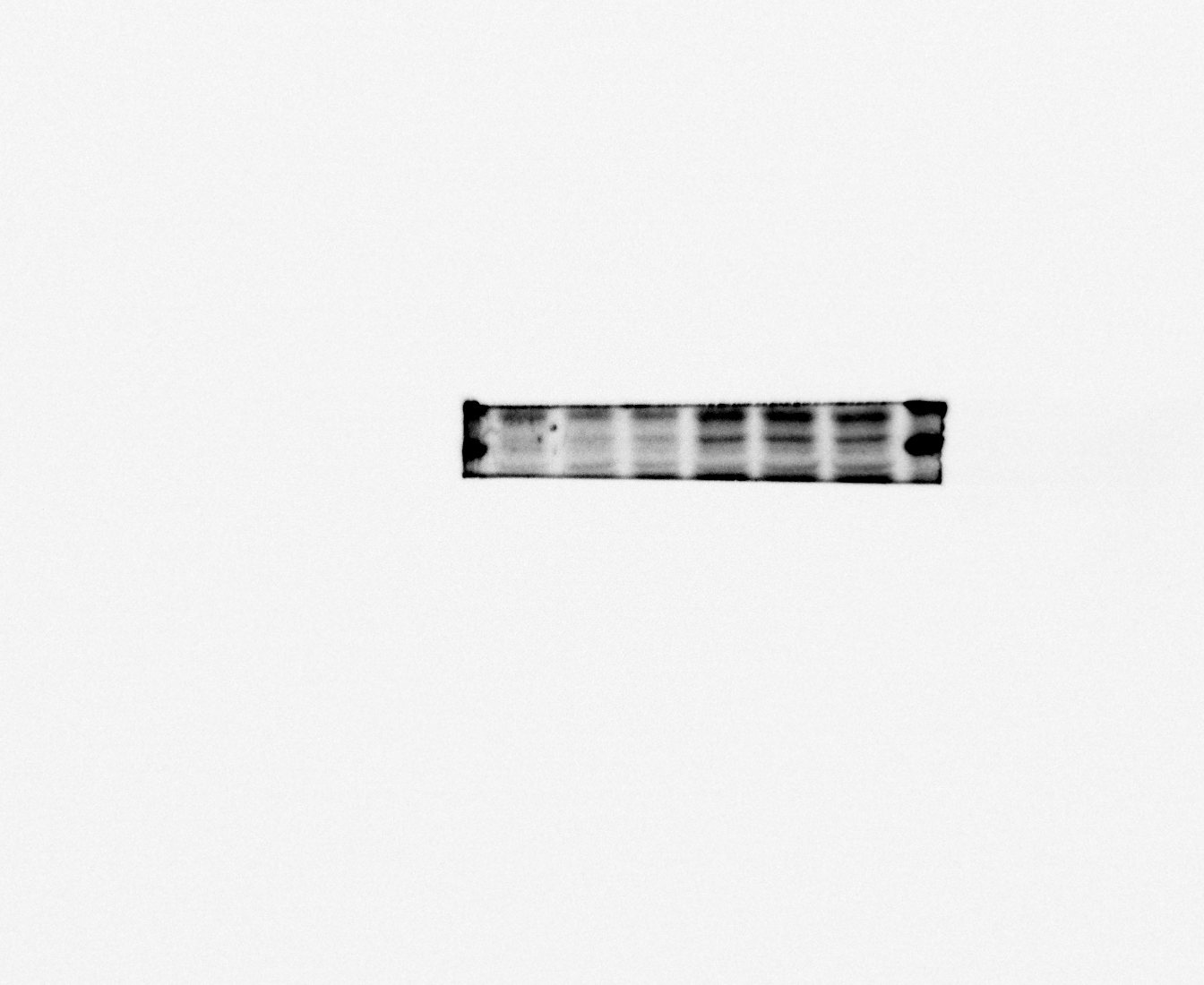

Supplement: Supplementary file 11 — Source data Fig. 5 [file 44321_2025_335_MOESM11_ESM.zip › Figure 5/Figure 5-A/western p-AMPK.tif]

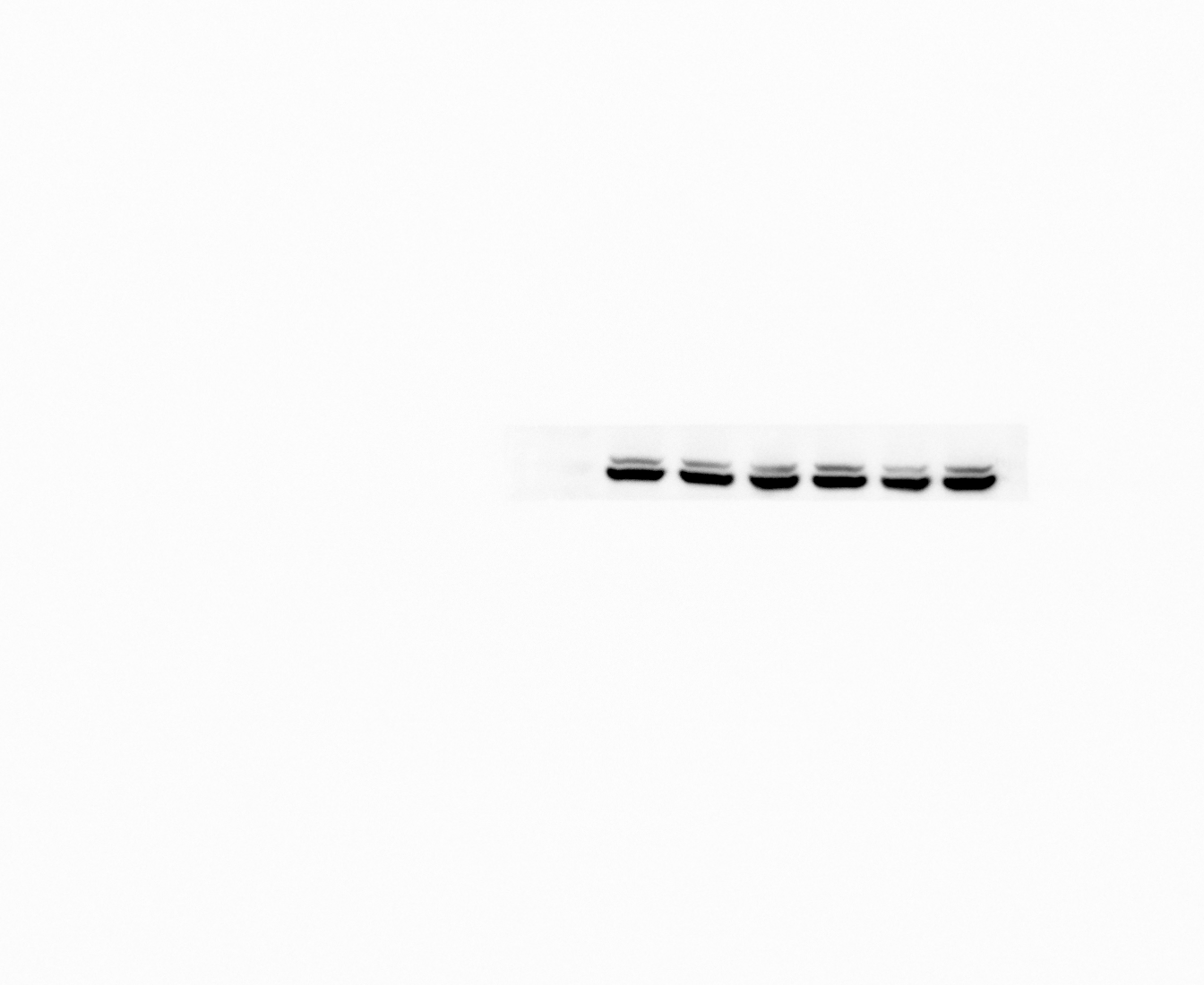

Supplement: Supplementary file 11 — Source data Fig. 5 [file 44321_2025_335_MOESM11_ESM.zip › Figure 5/Figure 5-A/western Hsp90.tif]

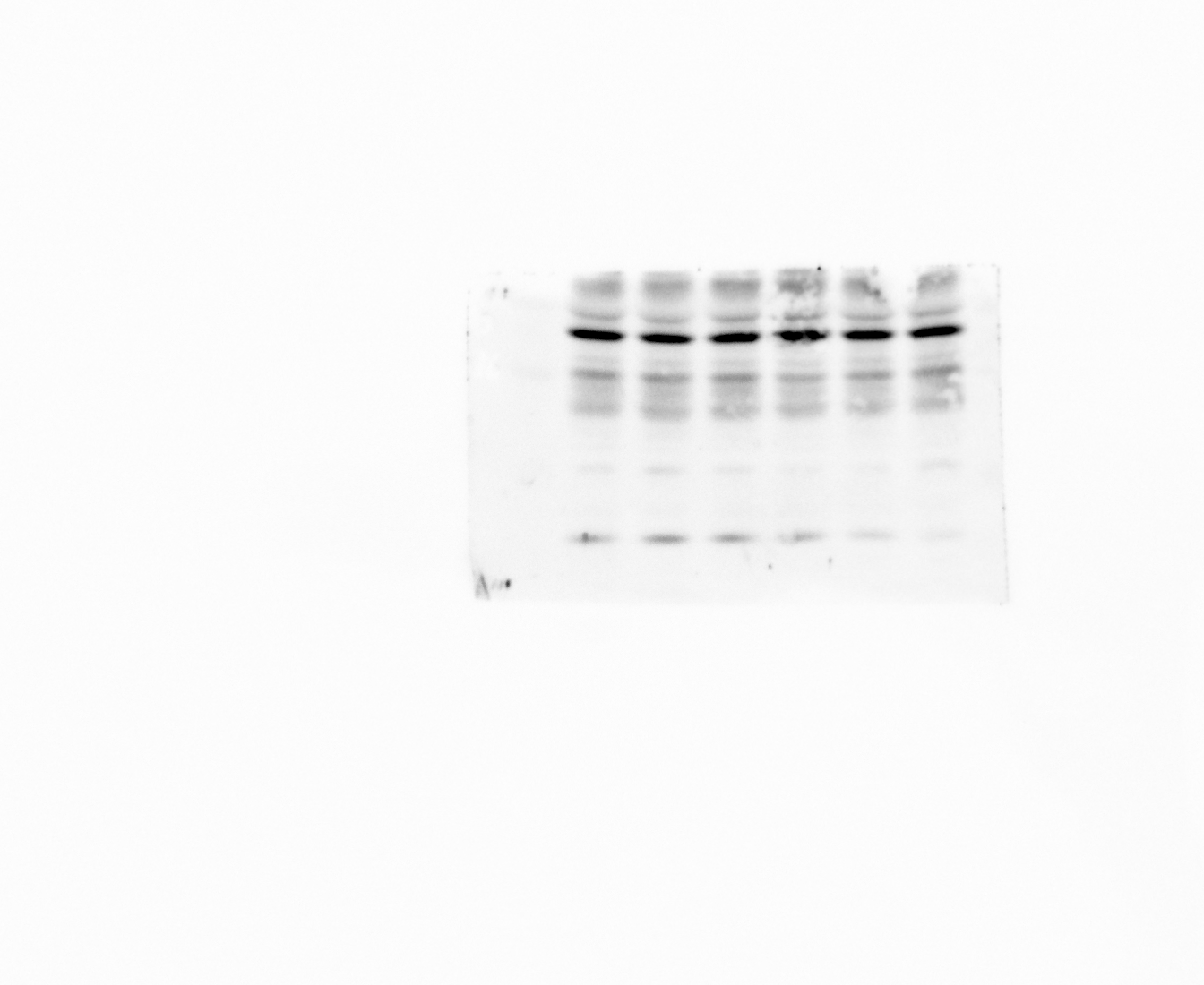

Supplement: Supplementary file 11 — Source data Fig. 5 [file 44321_2025_335_MOESM11_ESM.zip › Figure 5/Figure 5-A/western P38.tif]

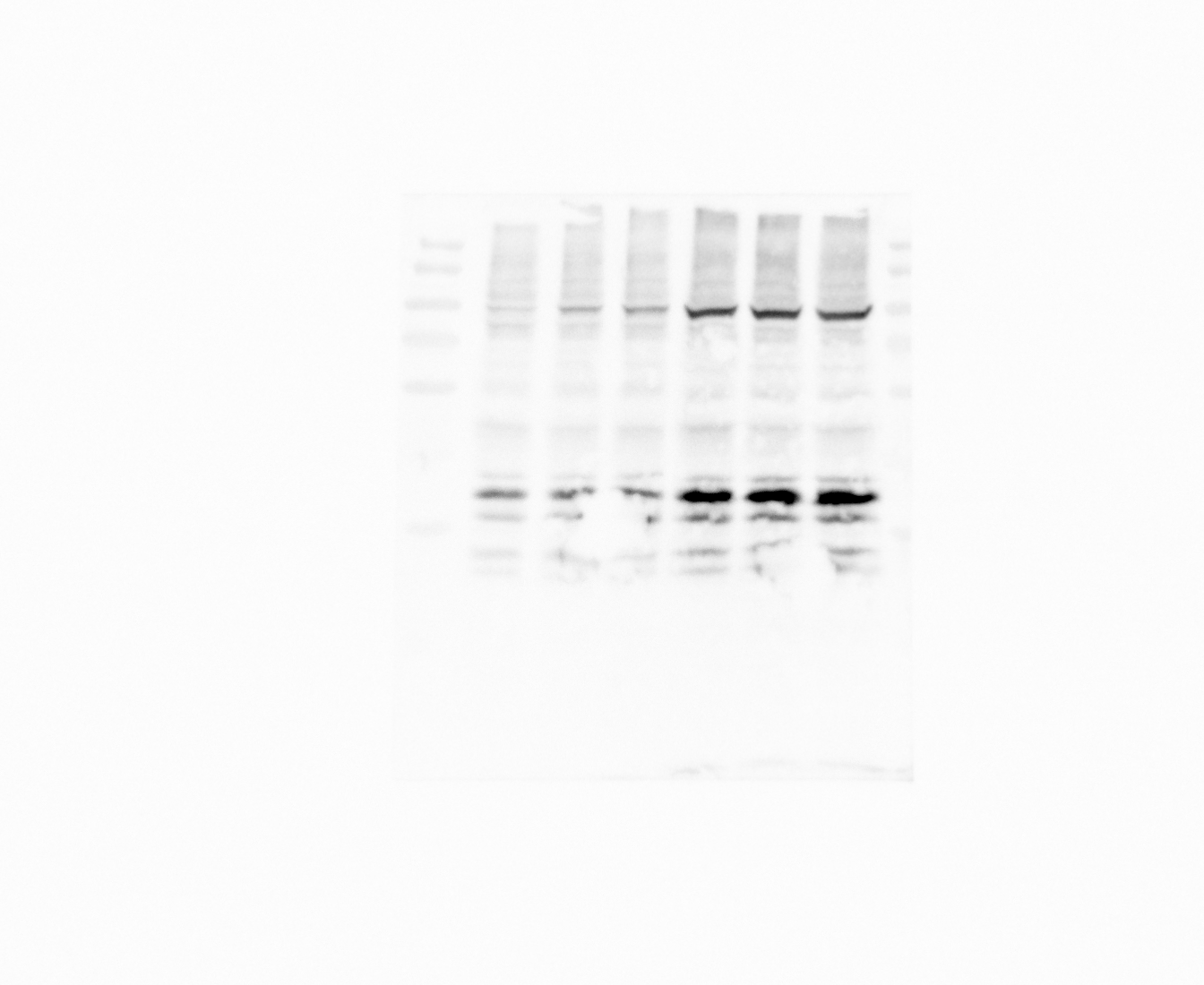

Supplement: Supplementary file 11 — Source data Fig. 5 [file 44321_2025_335_MOESM11_ESM.zip › Figure 5/Figure 5-A/western p-PKA substrate.tif]

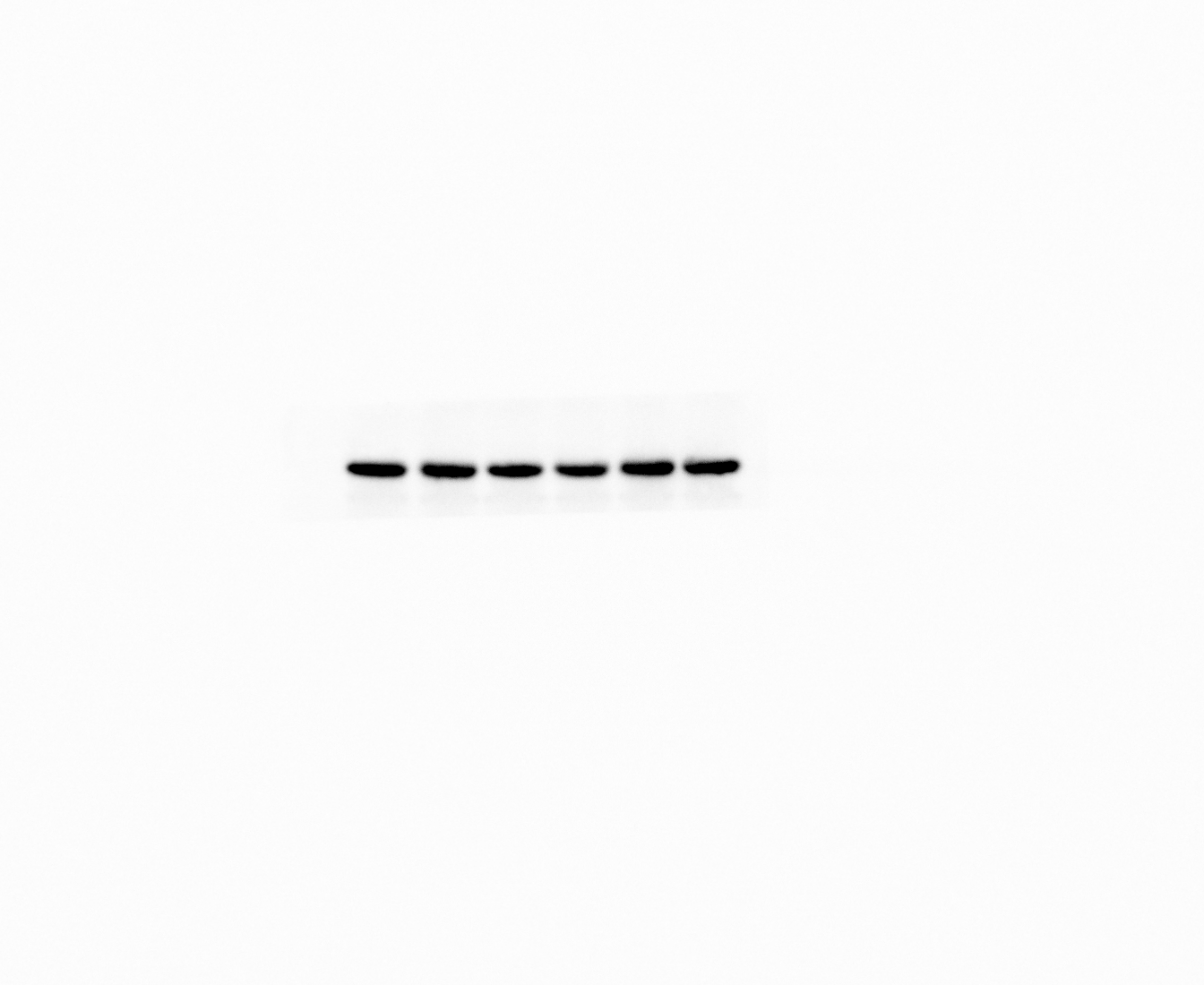

Supplement: Supplementary file 11 — Source data Fig. 5 [file 44321_2025_335_MOESM11_ESM.zip › Figure 5/Figure 5-A/western PKA.tif]

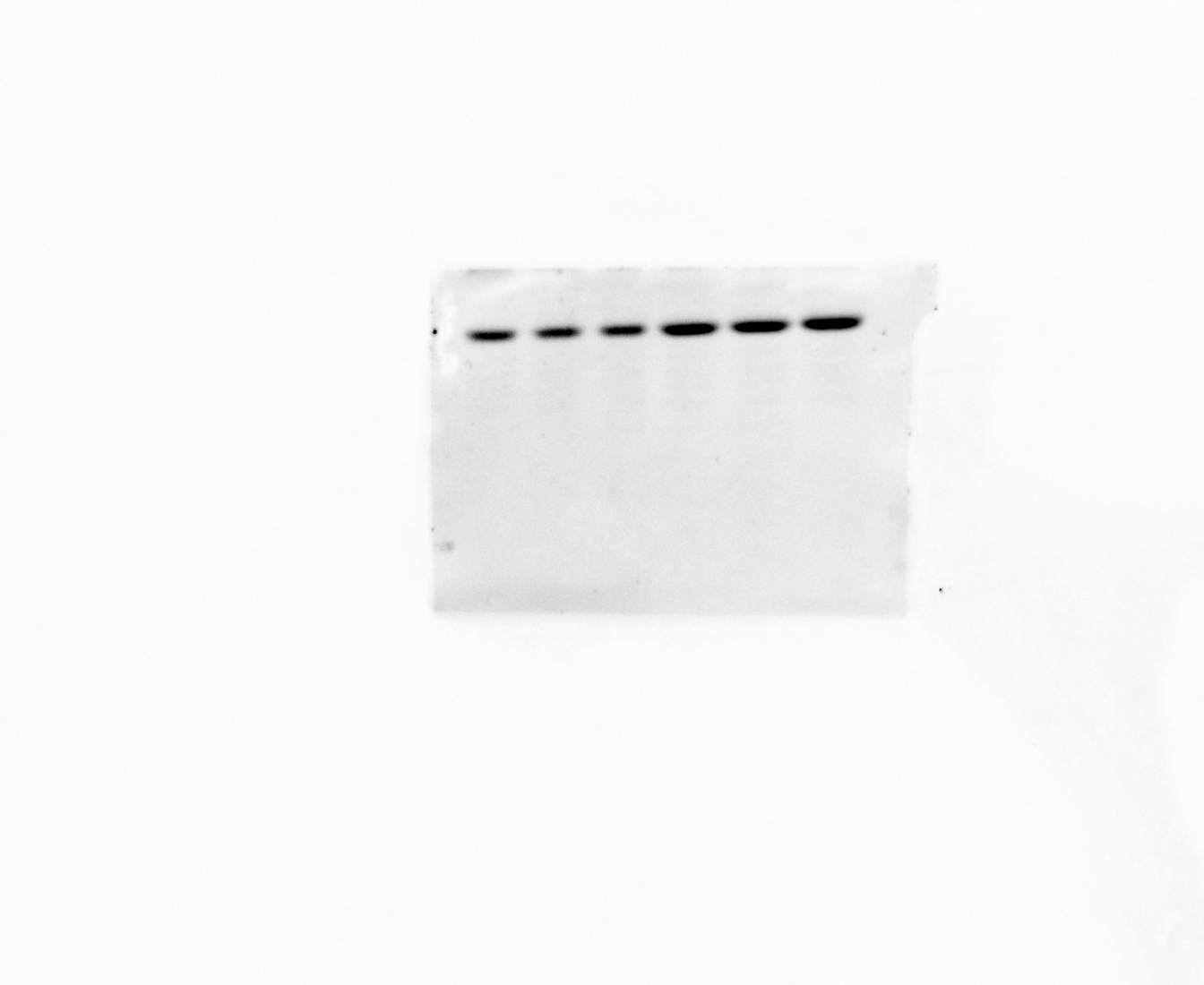

Supplement: Supplementary file 11 — Source data Fig. 5 [file 44321_2025_335_MOESM11_ESM.zip › Figure 5/Figure 5-A/western p-P38.tif]

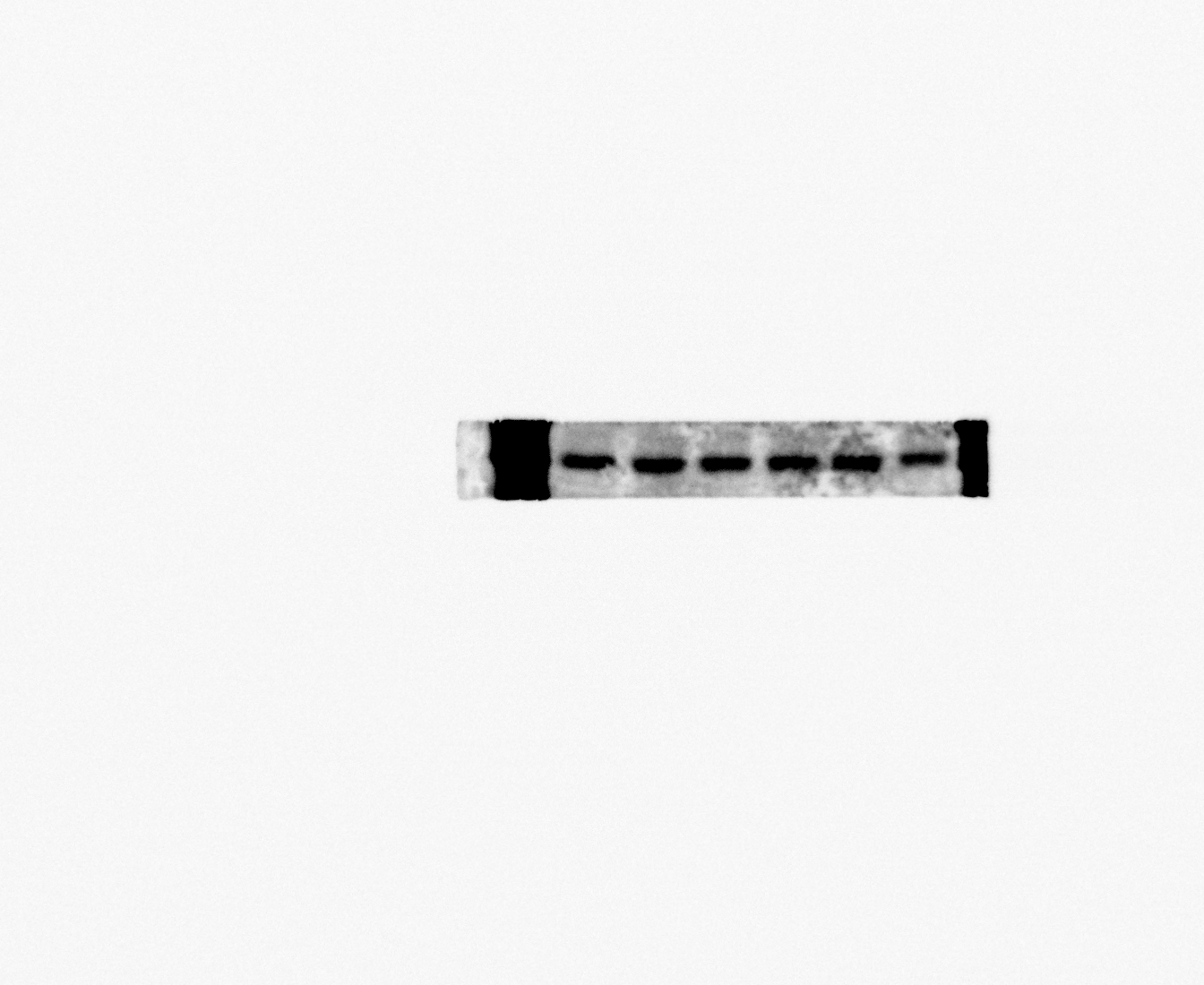

Supplement: Supplementary file 11 — Source data Fig. 5 [file 44321_2025_335_MOESM11_ESM.zip › Figure 5/Figure 5-A/western AMPK.tif]

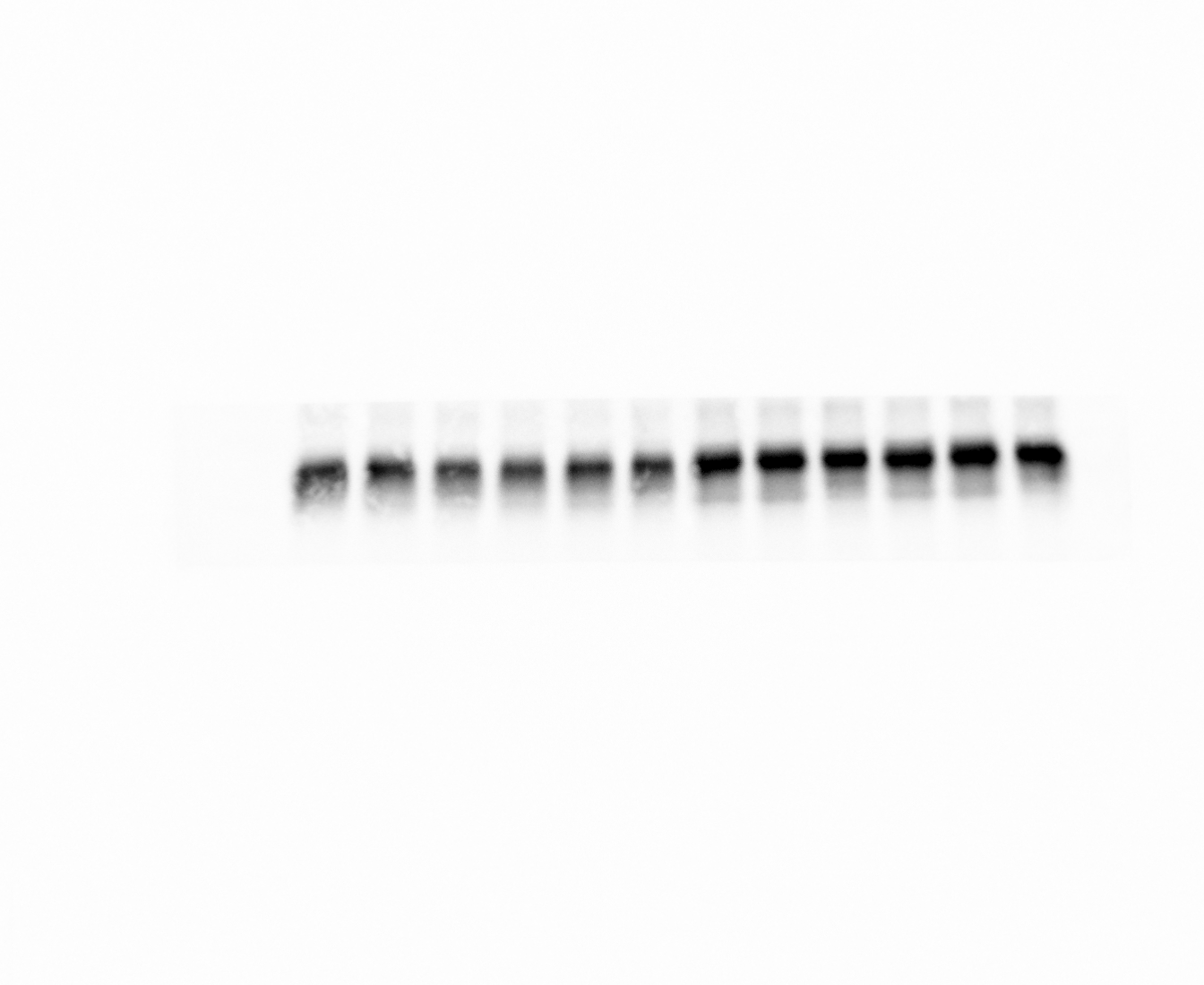

Supplement: Supplementary file 12 — Source data Fig. 6 [file 44321_2025_335_MOESM12_ESM.zip › Figure 6/Figure 6-E/western Gnas.tif]

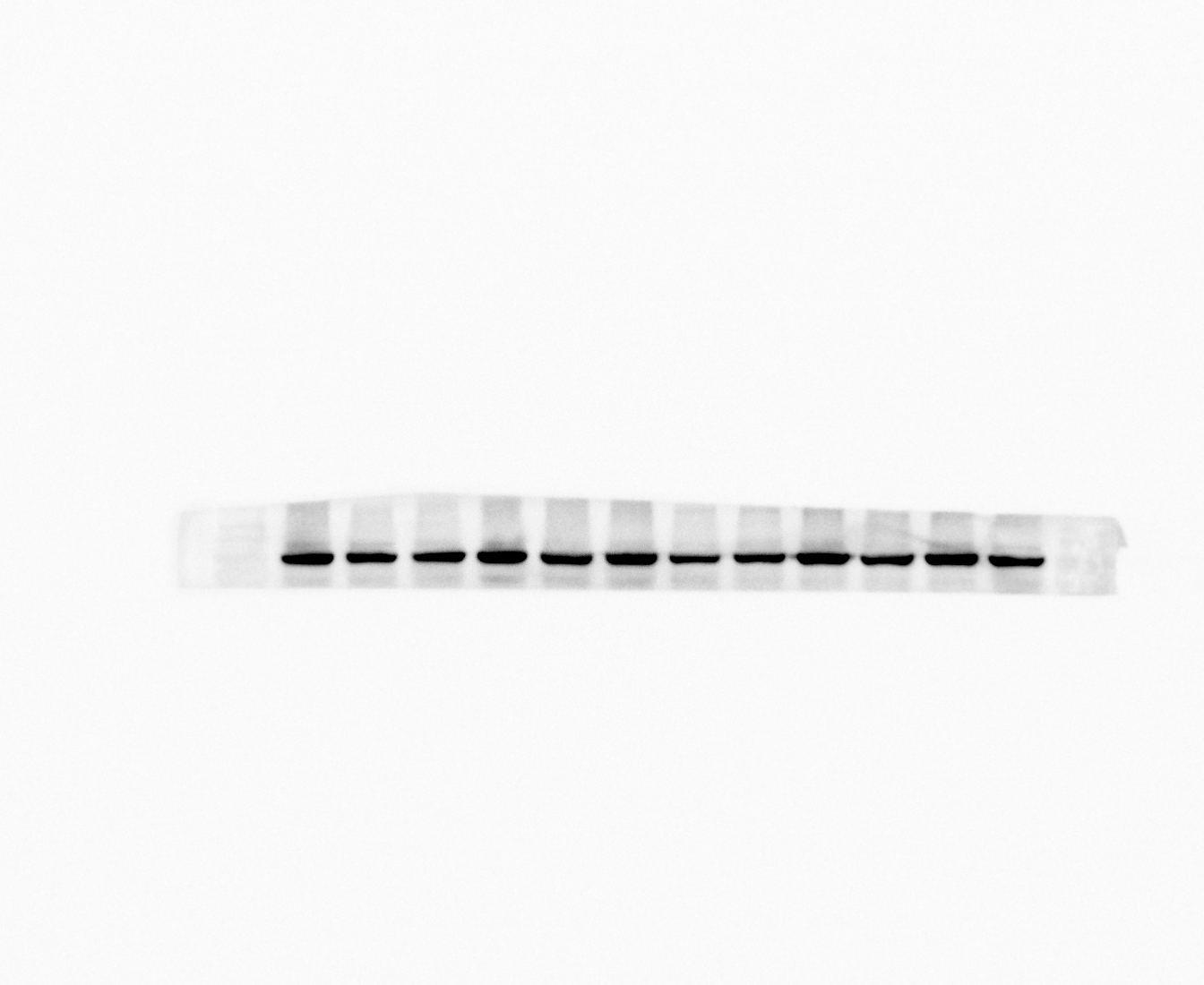

Supplement: Supplementary file 12 — Source data Fig. 6 [file 44321_2025_335_MOESM12_ESM.zip › Figure 6/Figure 6-E/western Hsp90.tif]

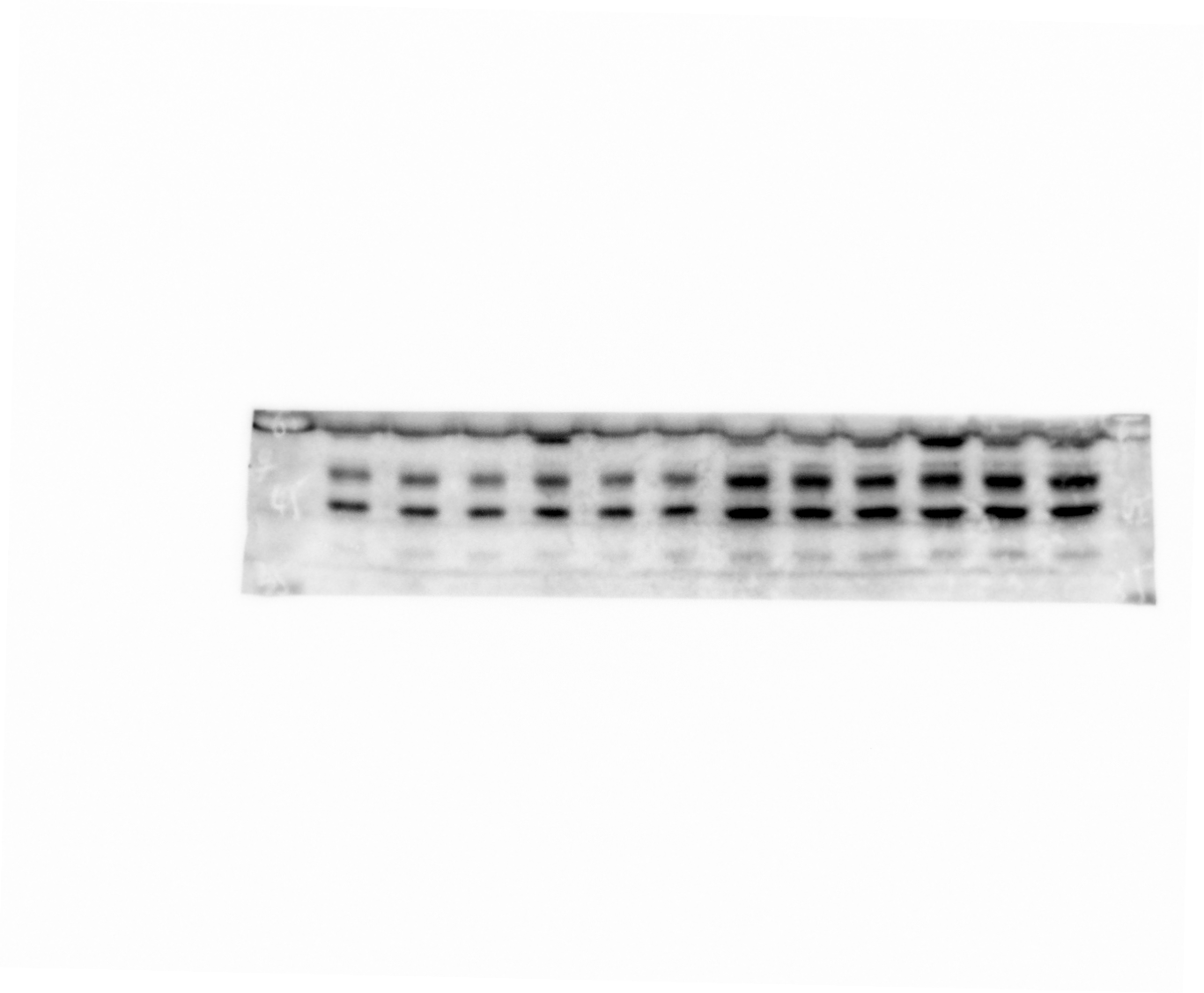

Supplement: Supplementary file 12 — Source data Fig. 6 [file 44321_2025_335_MOESM12_ESM.zip › Figure 6/Figure 6-D/western Gnas.tif]

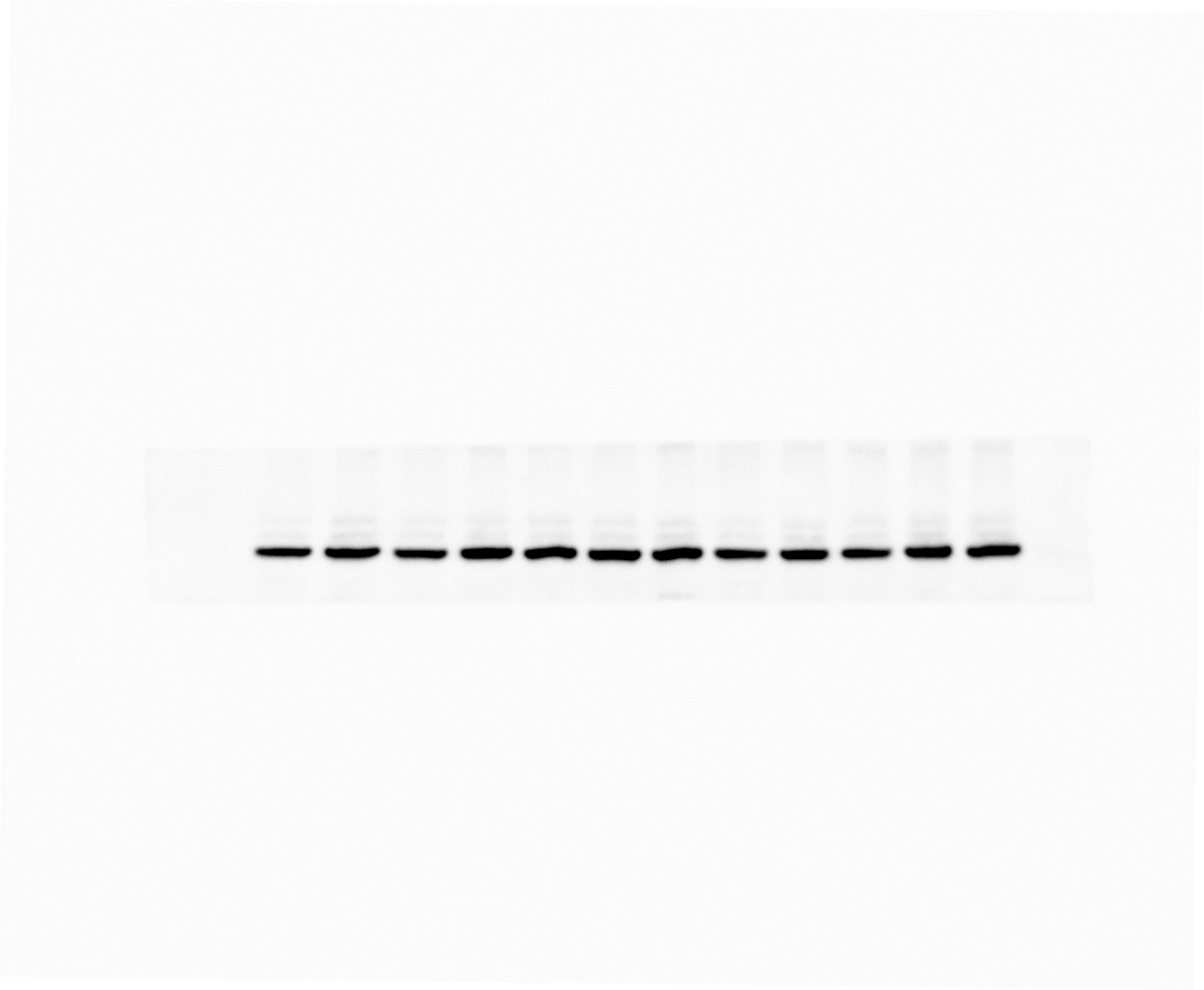

Supplement: Supplementary file 12 — Source data Fig. 6 [file 44321_2025_335_MOESM12_ESM.zip › Figure 6/Figure 6-D/western Hsp90.tif]

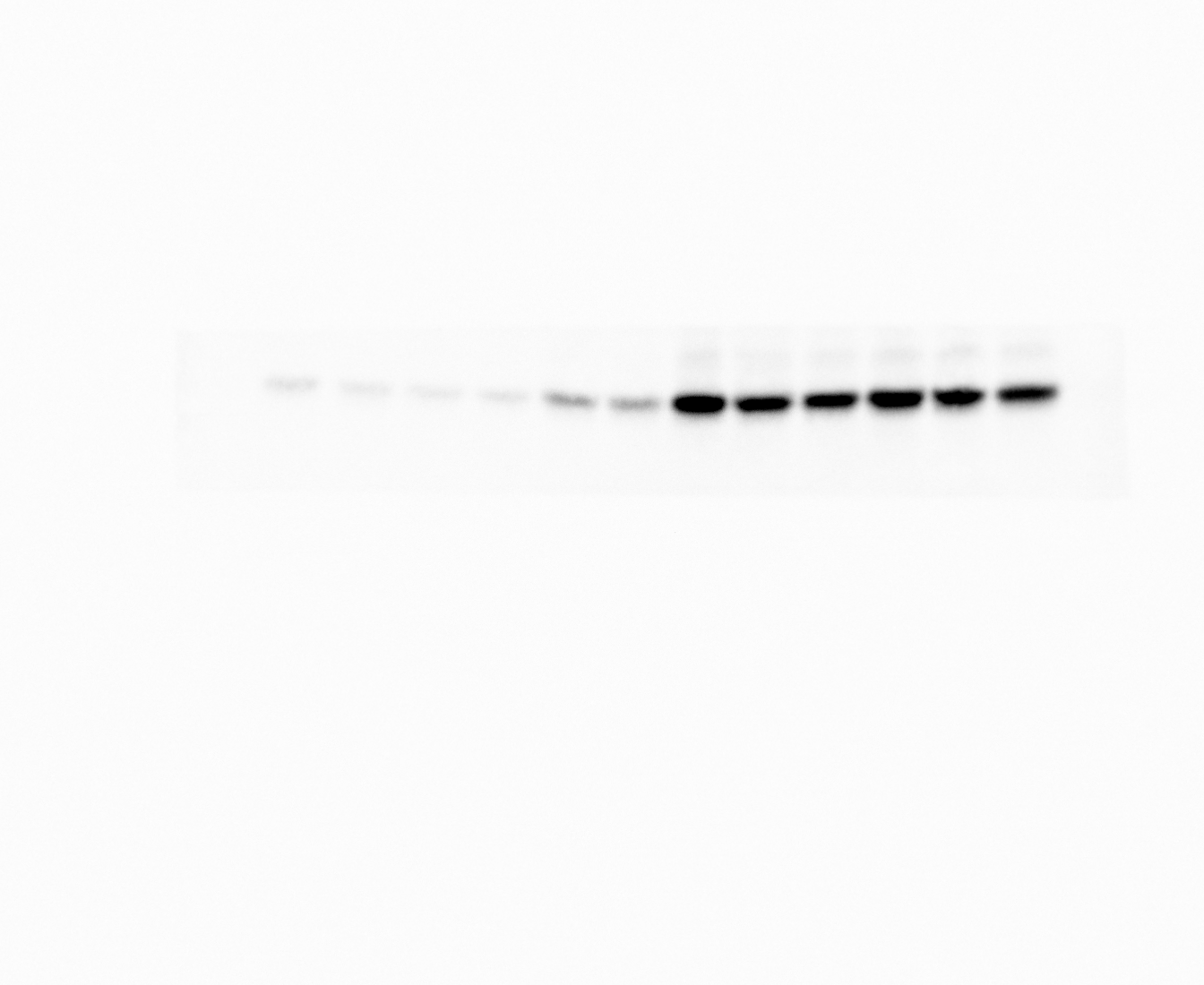

Supplement: Supplementary file 12 — Source data Fig. 6 [file 44321_2025_335_MOESM12_ESM.zip › Figure 6/Figure 6-D/western Ucp1.tif]

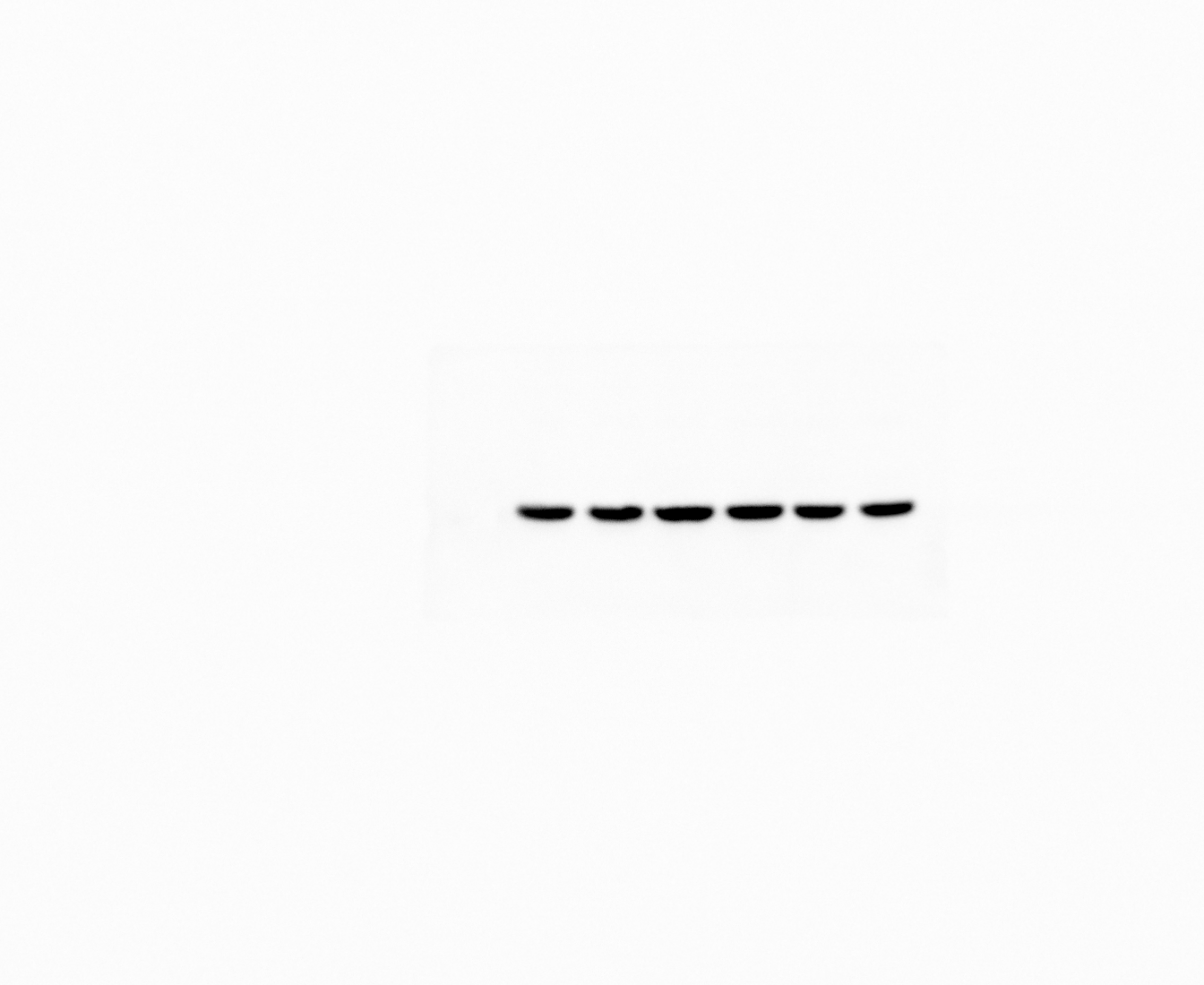

Supplement: Supplementary file 12 — Source data Fig. 6 [file 44321_2025_335_MOESM12_ESM.zip › Figure 6/Figure 6-J/western actin.tif]

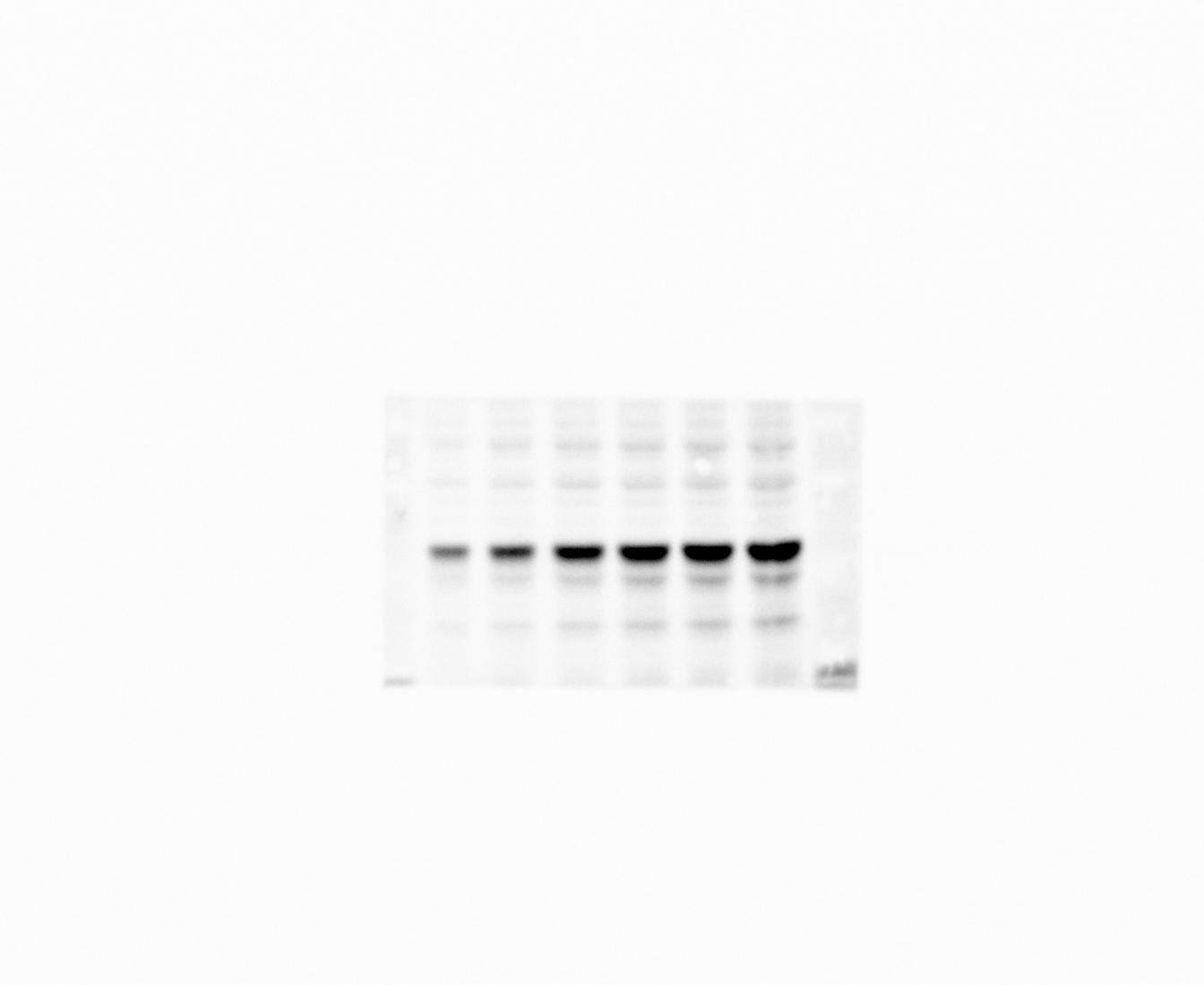

Supplement: Supplementary file 12 — Source data Fig. 6 [file 44321_2025_335_MOESM12_ESM.zip › Figure 6/Figure 6-J/western Gnas.tif]

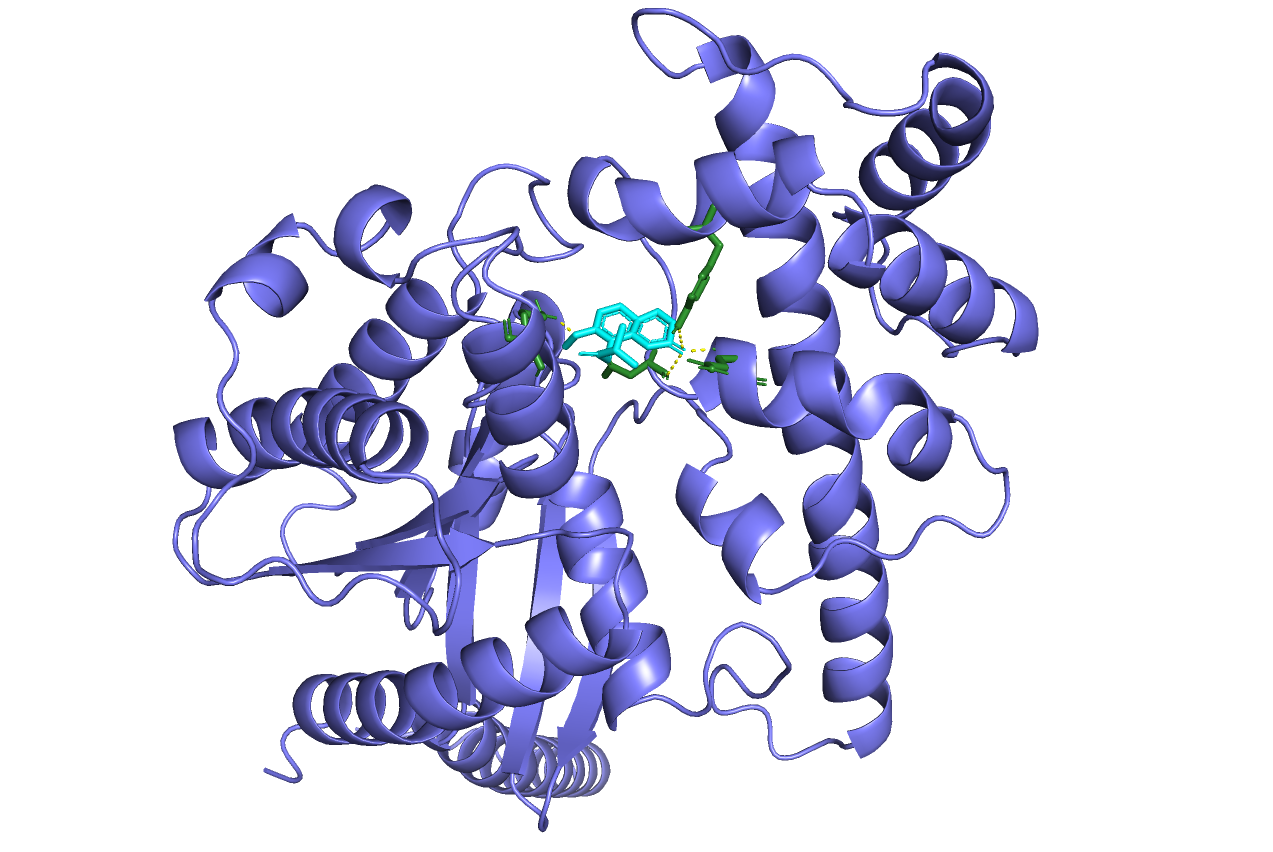

Supplement: Supplementary file 12 — Source data Fig. 6 [file 44321_2025_335_MOESM12_ESM.zip › Figure 6/Figure 6-F/autodock-1.tif]

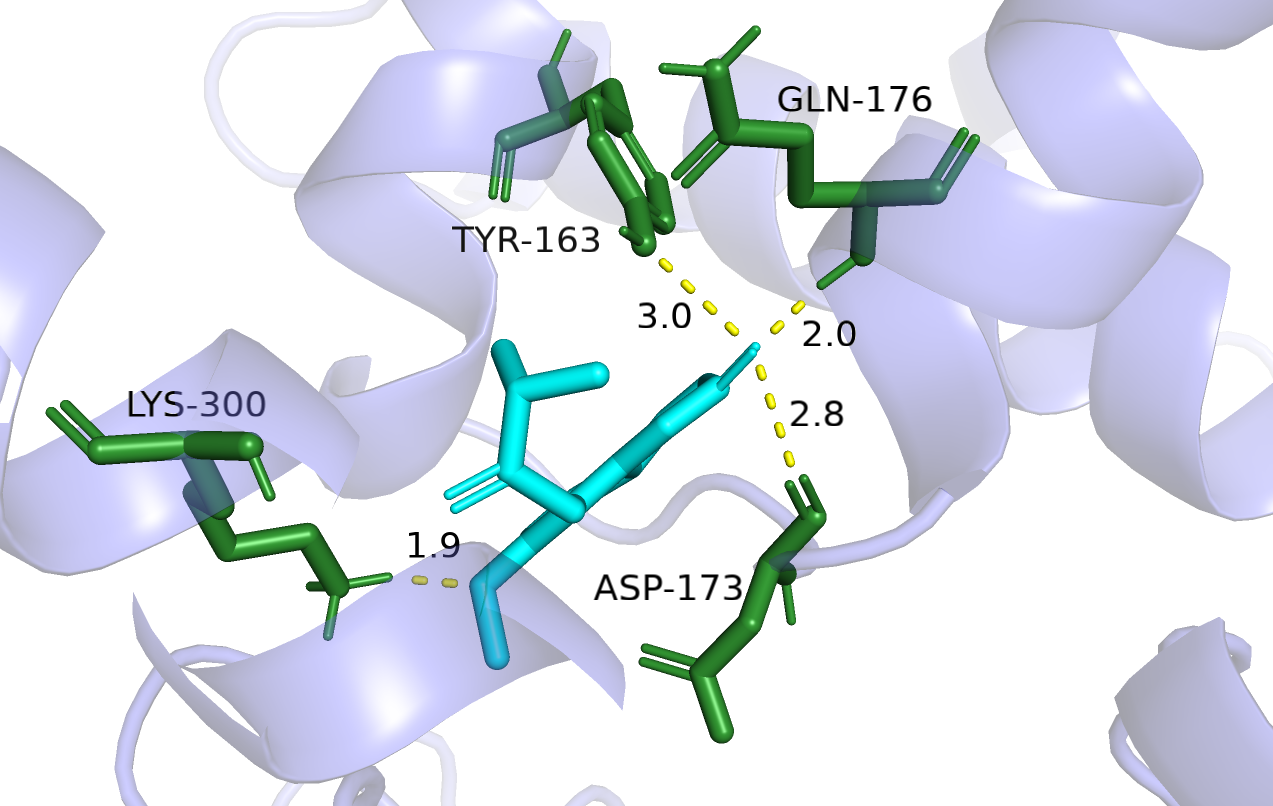

Supplement: Supplementary file 12 — Source data Fig. 6 [file 44321_2025_335_MOESM12_ESM.zip › Figure 6/Figure 6-F/autodock-2.tif]

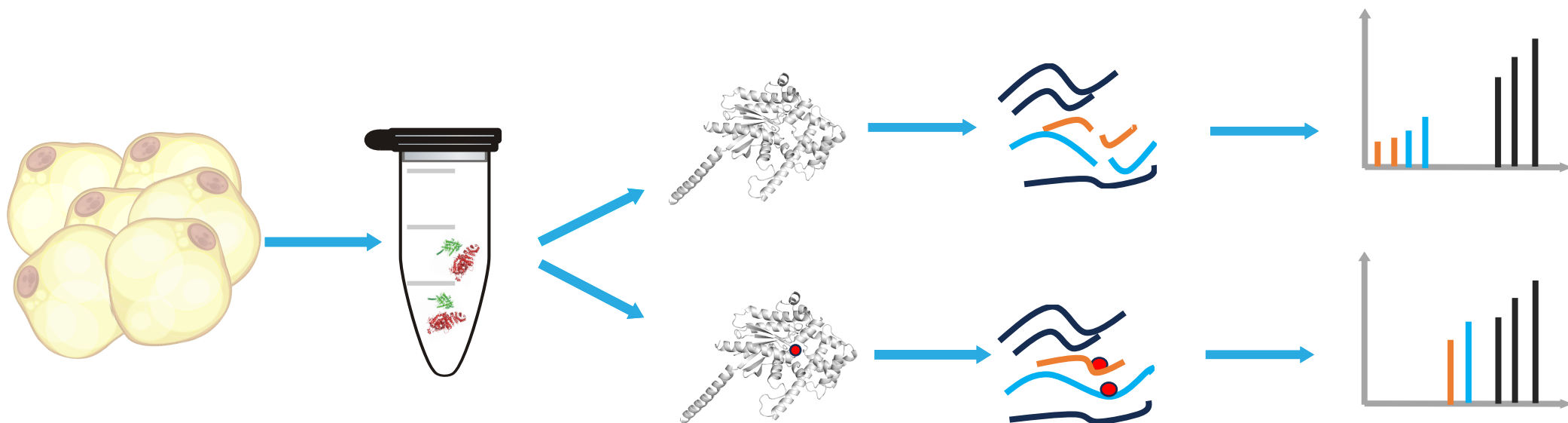

Supplement: Supplementary file 12 — Source data Fig. 6 [file 44321_2025_335_MOESM12_ESM.zip › Figure 6/Figure 6-A/A-Lip-smap.pdf]

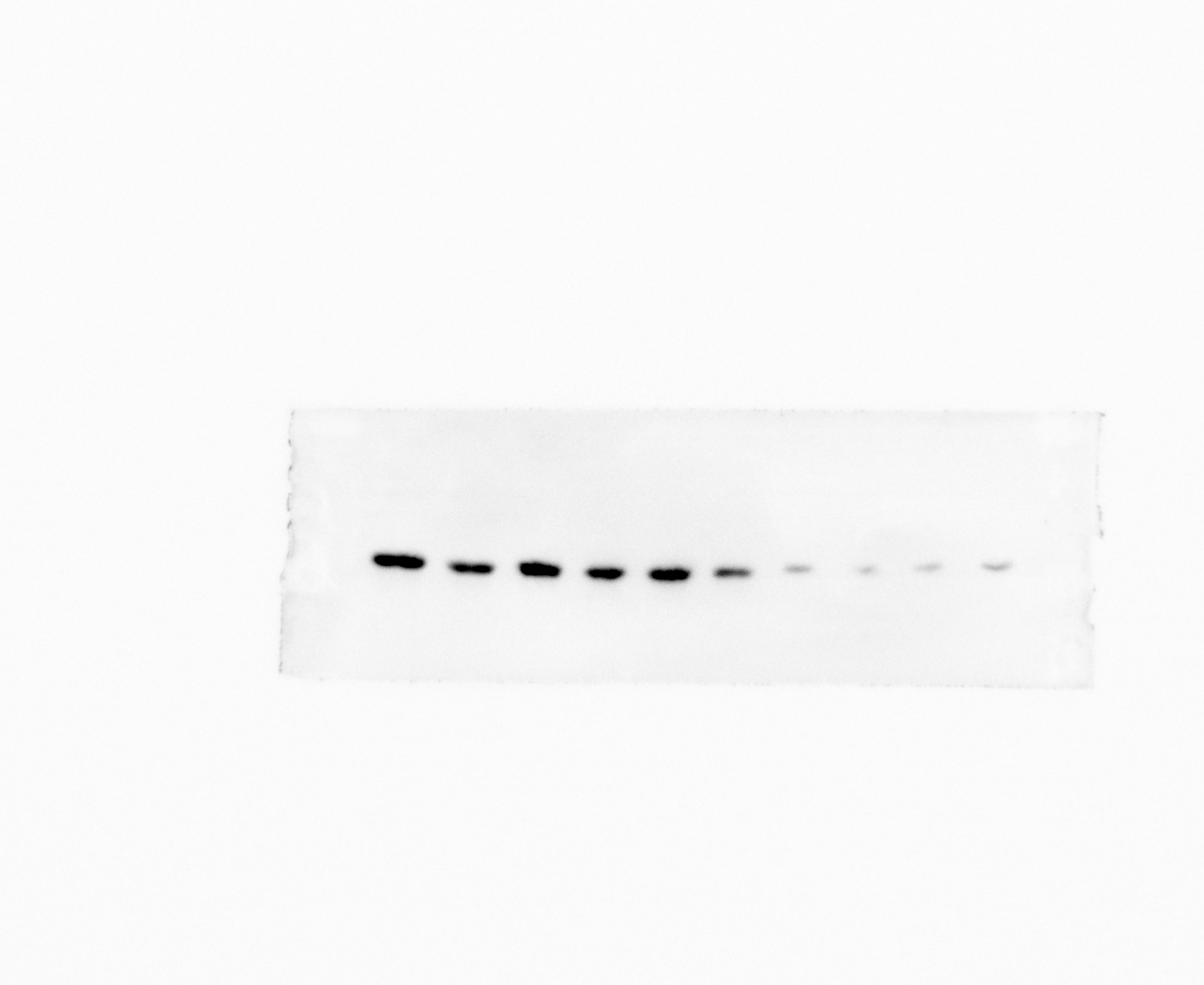

Supplement: Supplementary file 12 — Source data Fig. 6 [file 44321_2025_335_MOESM12_ESM.zip › Figure 6/Figure 6-G/western dmso actin.tif]

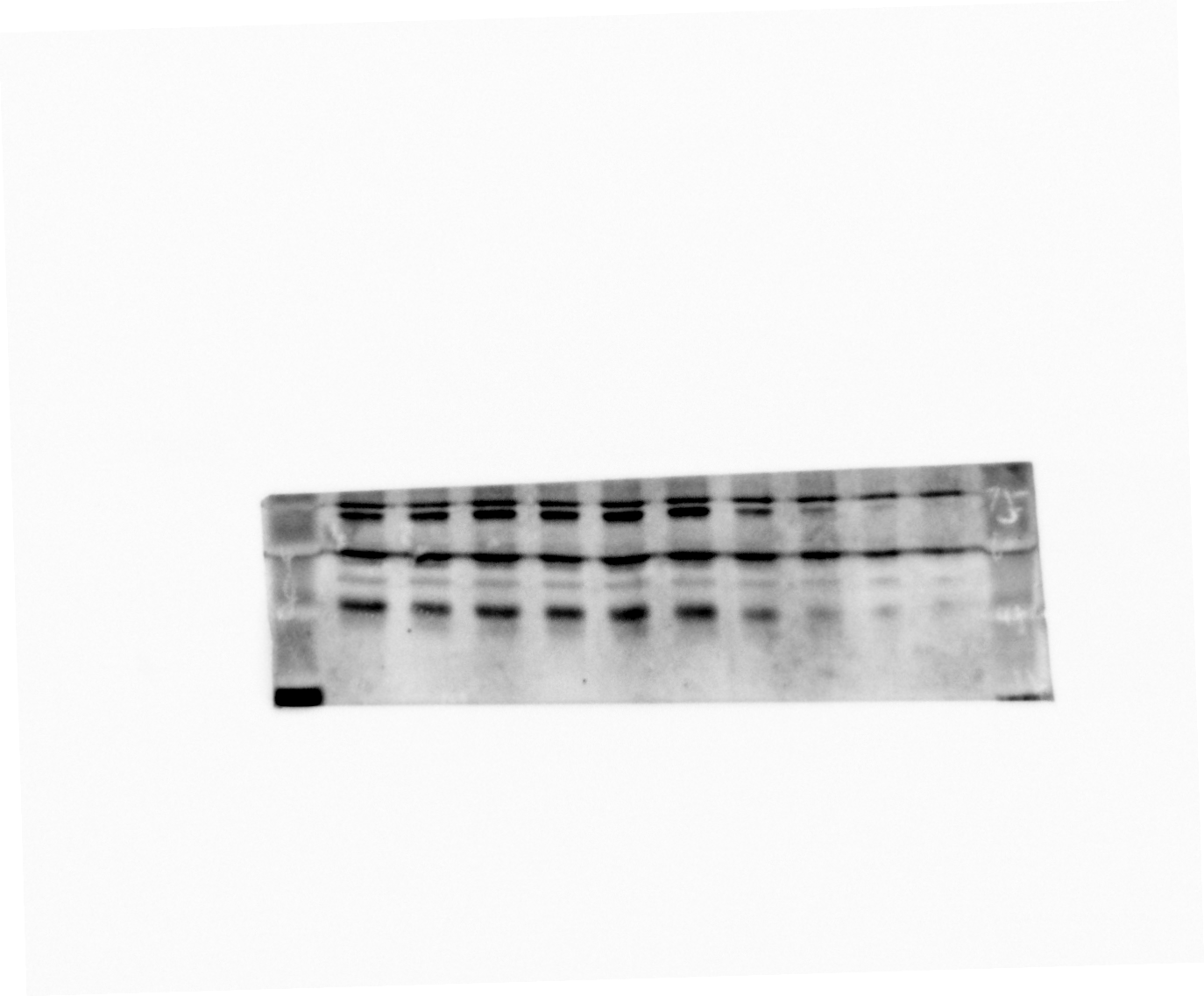

Supplement: Supplementary file 12 — Source data Fig. 6 [file 44321_2025_335_MOESM12_ESM.zip › Figure 6/Figure 6-G/western ism Gnas.tif]

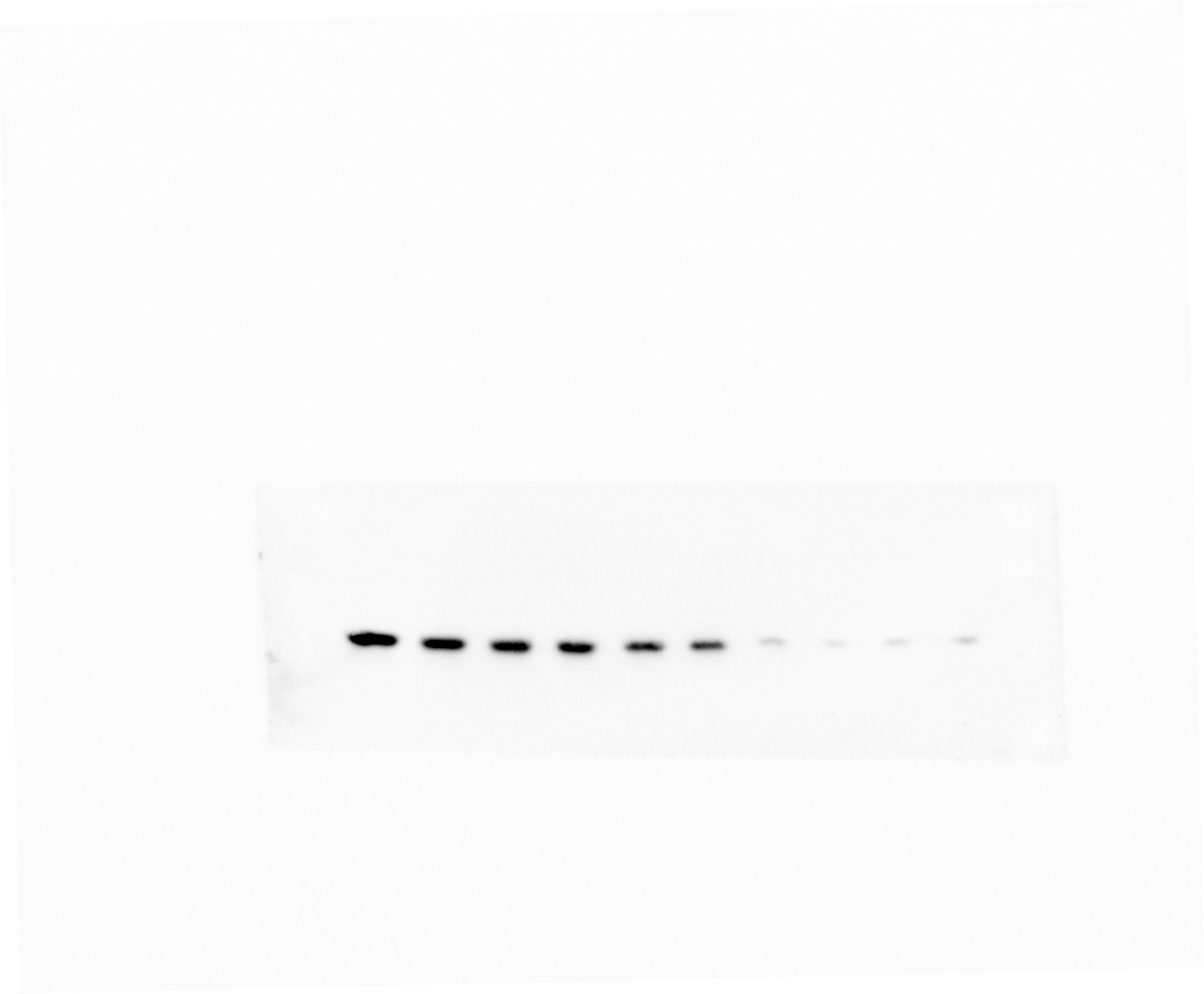

Supplement: Supplementary file 12 — Source data Fig. 6 [file 44321_2025_335_MOESM12_ESM.zip › Figure 6/Figure 6-G/western ism actin.tif]

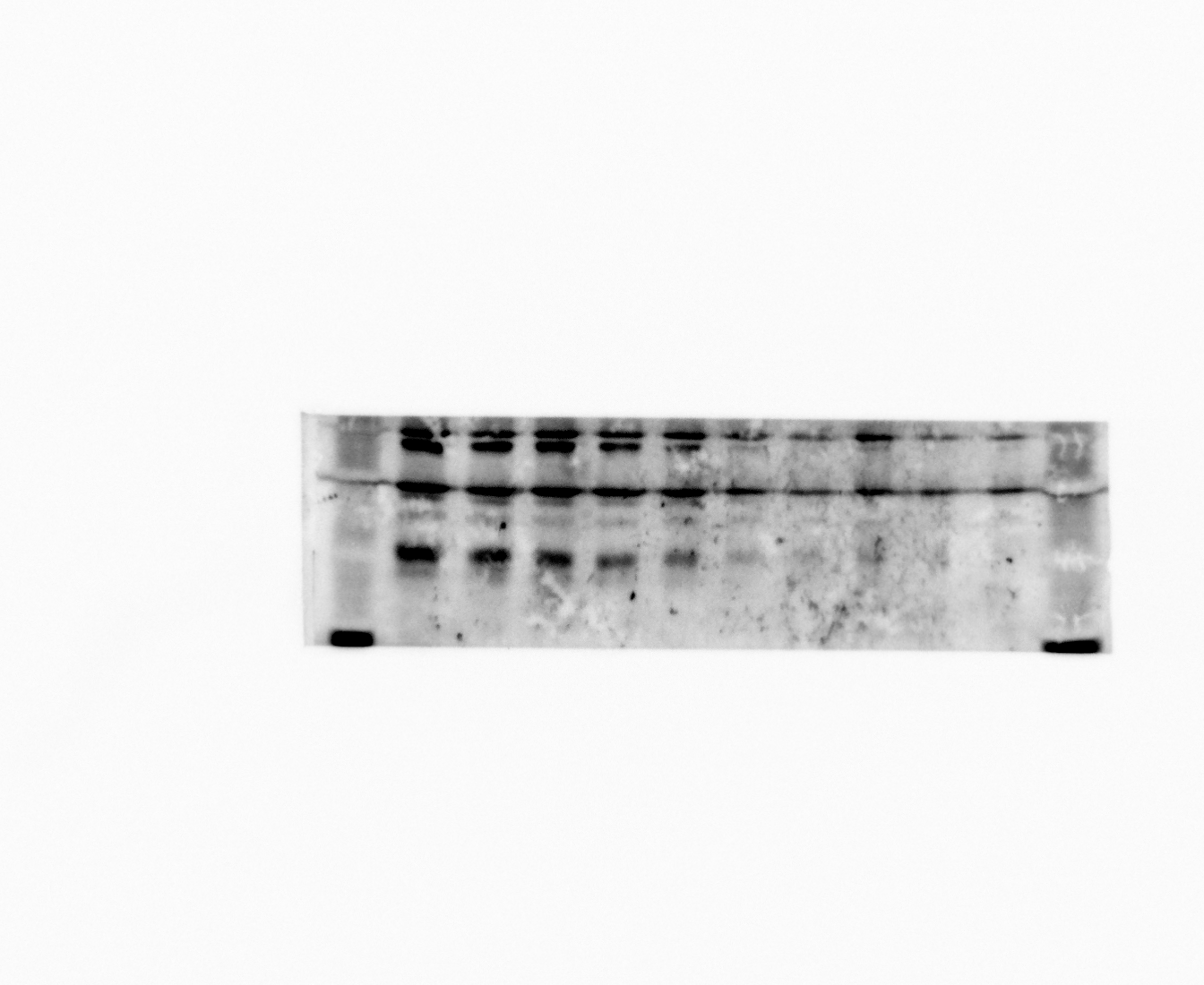

Supplement: Supplementary file 12 — Source data Fig. 6 [file 44321_2025_335_MOESM12_ESM.zip › Figure 6/Figure 6-G/western dmso Gnas.tif]

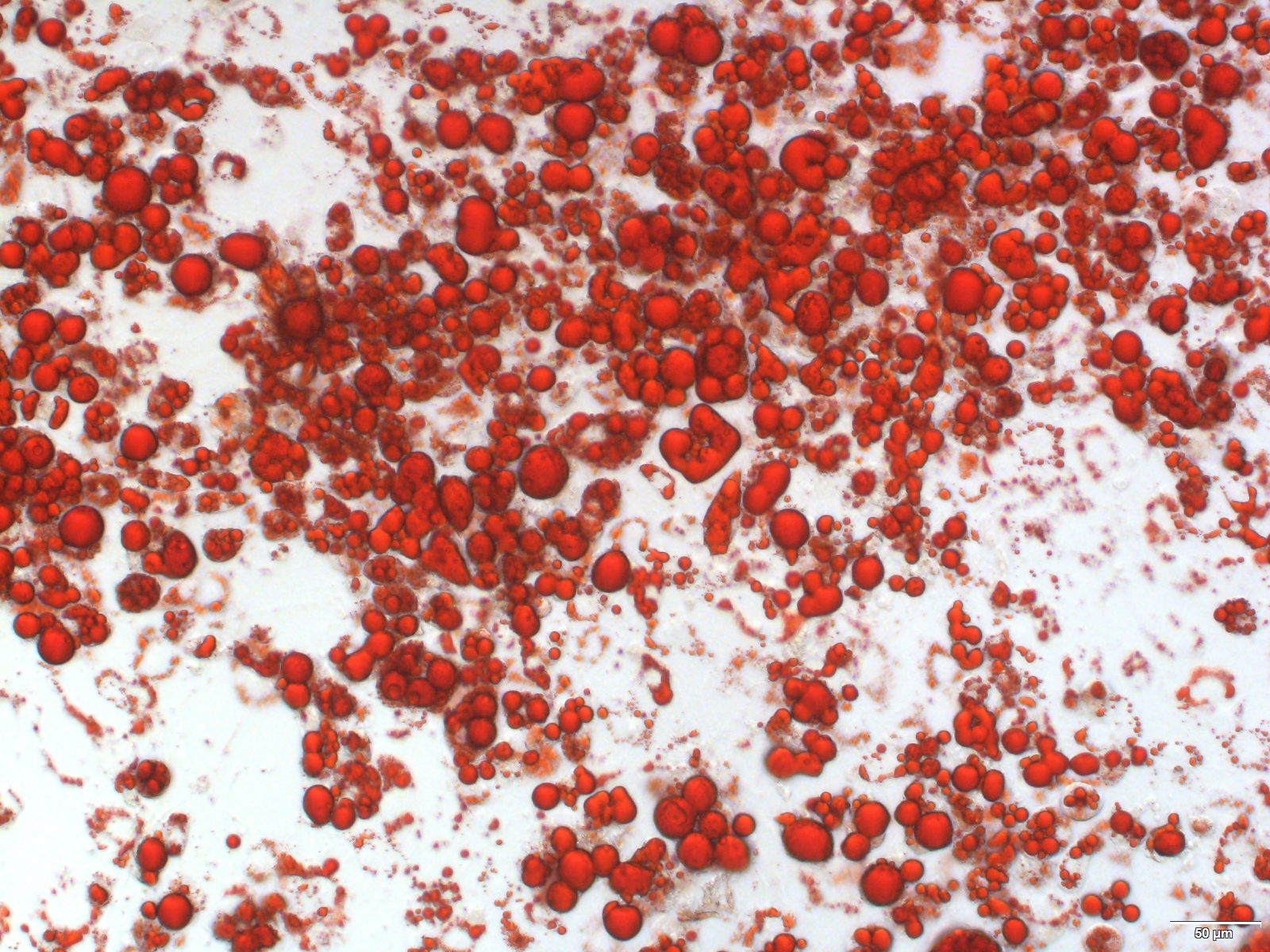

Supplement: Supplementary file 13 — Source data Fig. 7 [file 44321_2025_335_MOESM13_ESM.zip › Figure 7/Figure 7-J/Oil Red O staining/SQ22536-20X.jpg]

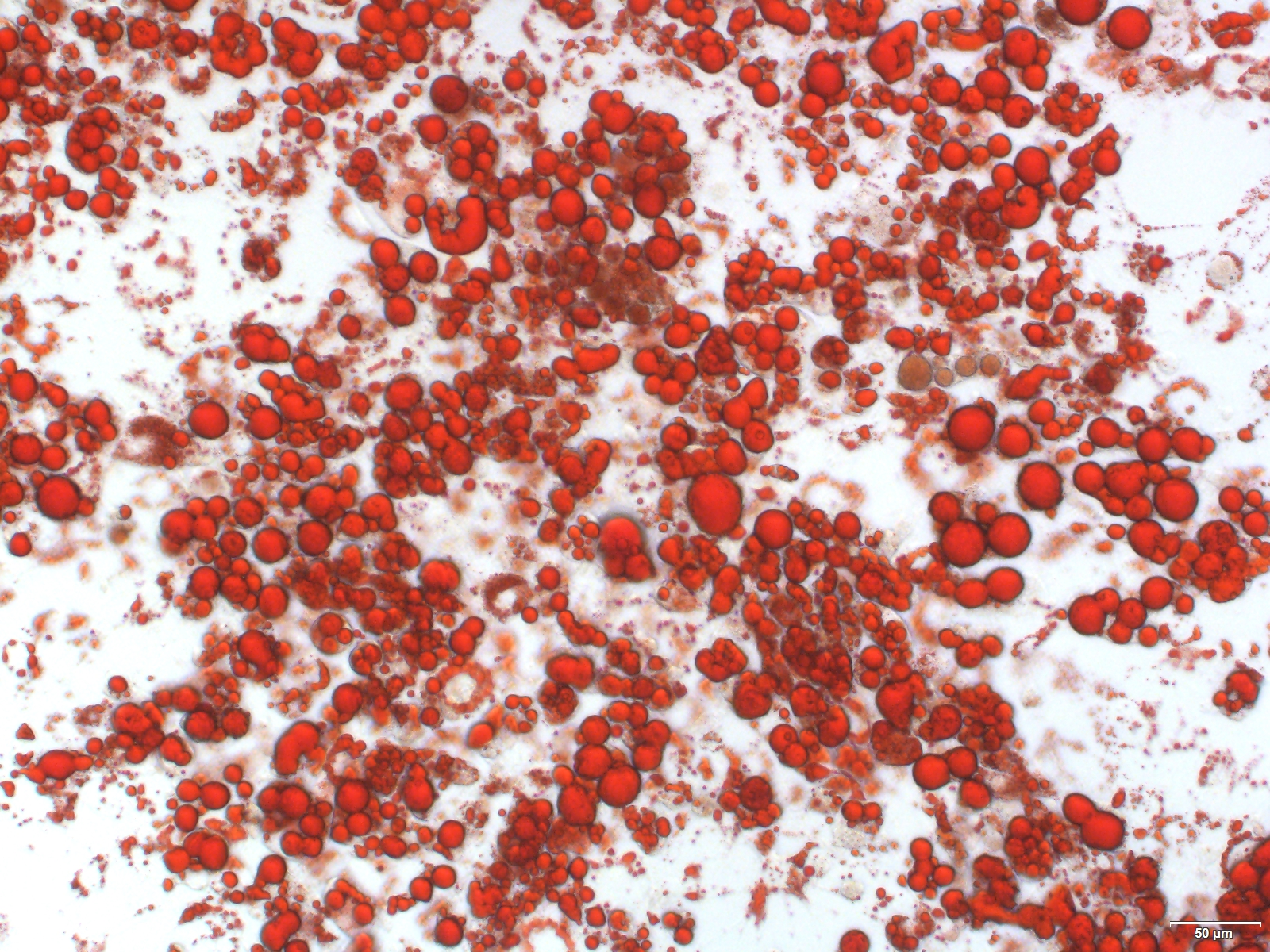

Supplement: Supplementary file 13 — Source data Fig. 7 [file 44321_2025_335_MOESM13_ESM.zip › Figure 7/Figure 7-J/Oil Red O staining/ISM+SQ22536.jpg]

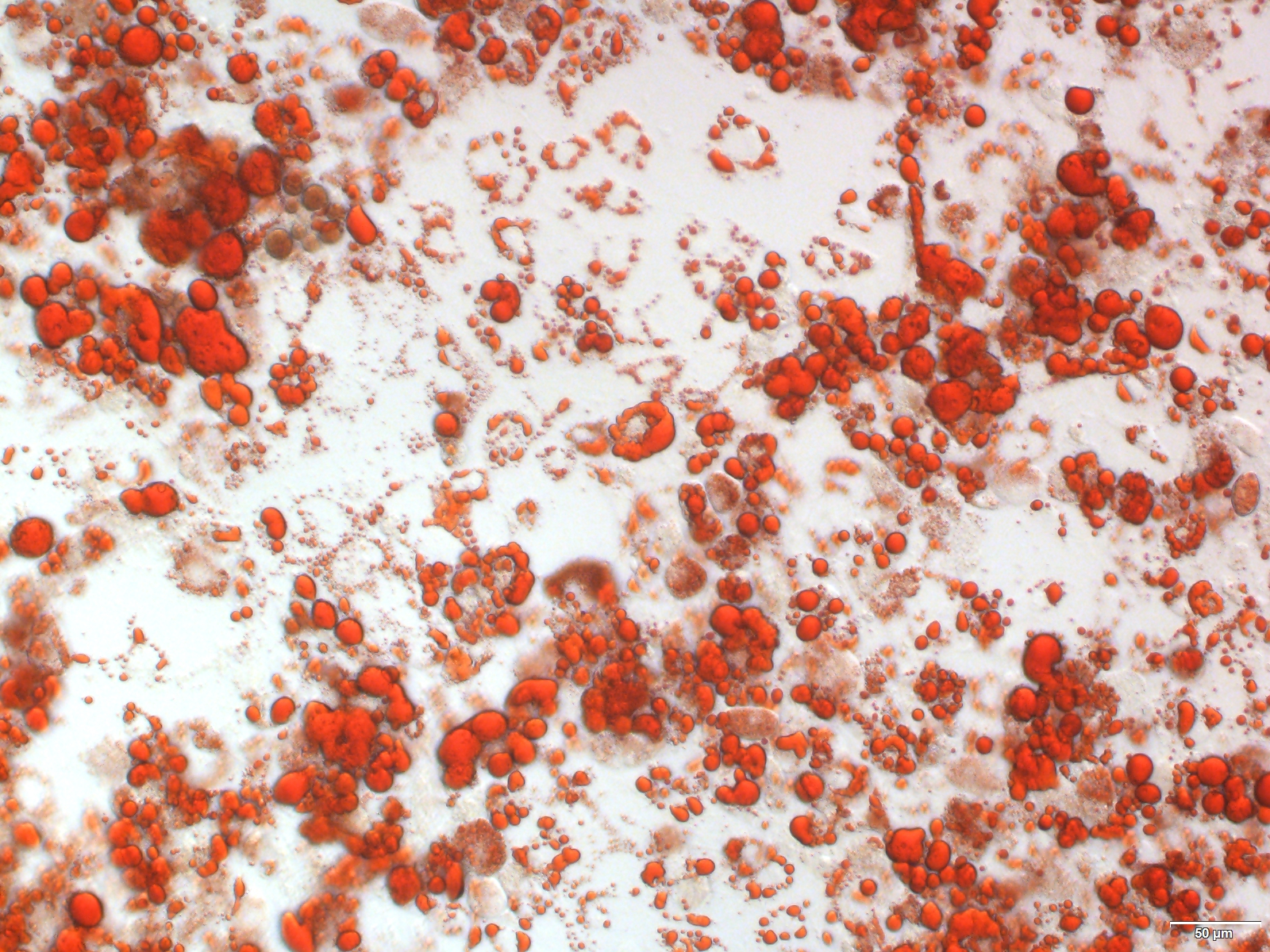

Supplement: Supplementary file 13 — Source data Fig. 7 [file 44321_2025_335_MOESM13_ESM.zip › Figure 7/Figure 7-J/Oil Red O staining/ISM-20X.jpg]

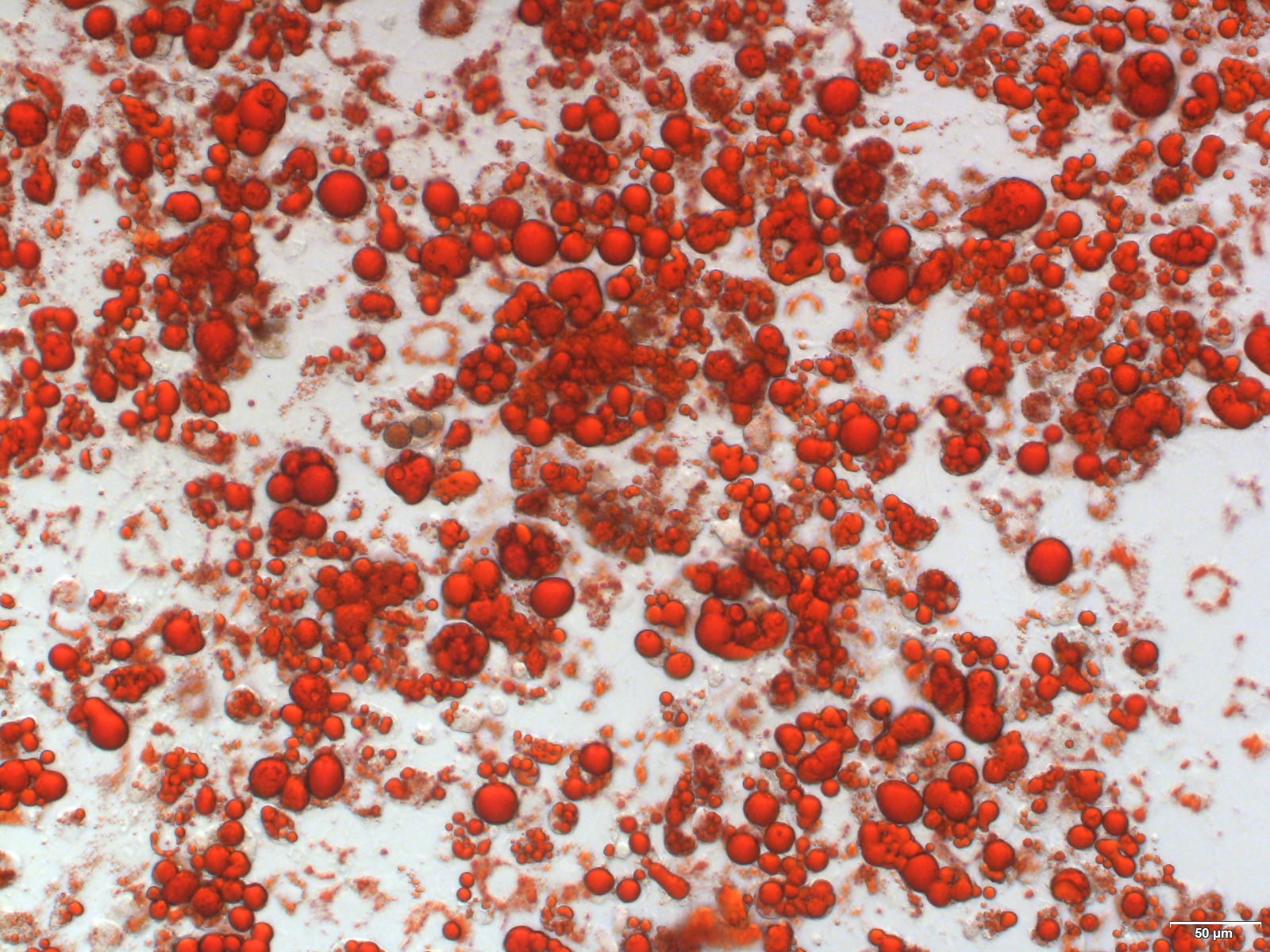

Supplement: Supplementary file 13 — Source data Fig. 7 [file 44321_2025_335_MOESM13_ESM.zip › Figure 7/Figure 7-J/Oil Red O staining/Control-20x.jpg]

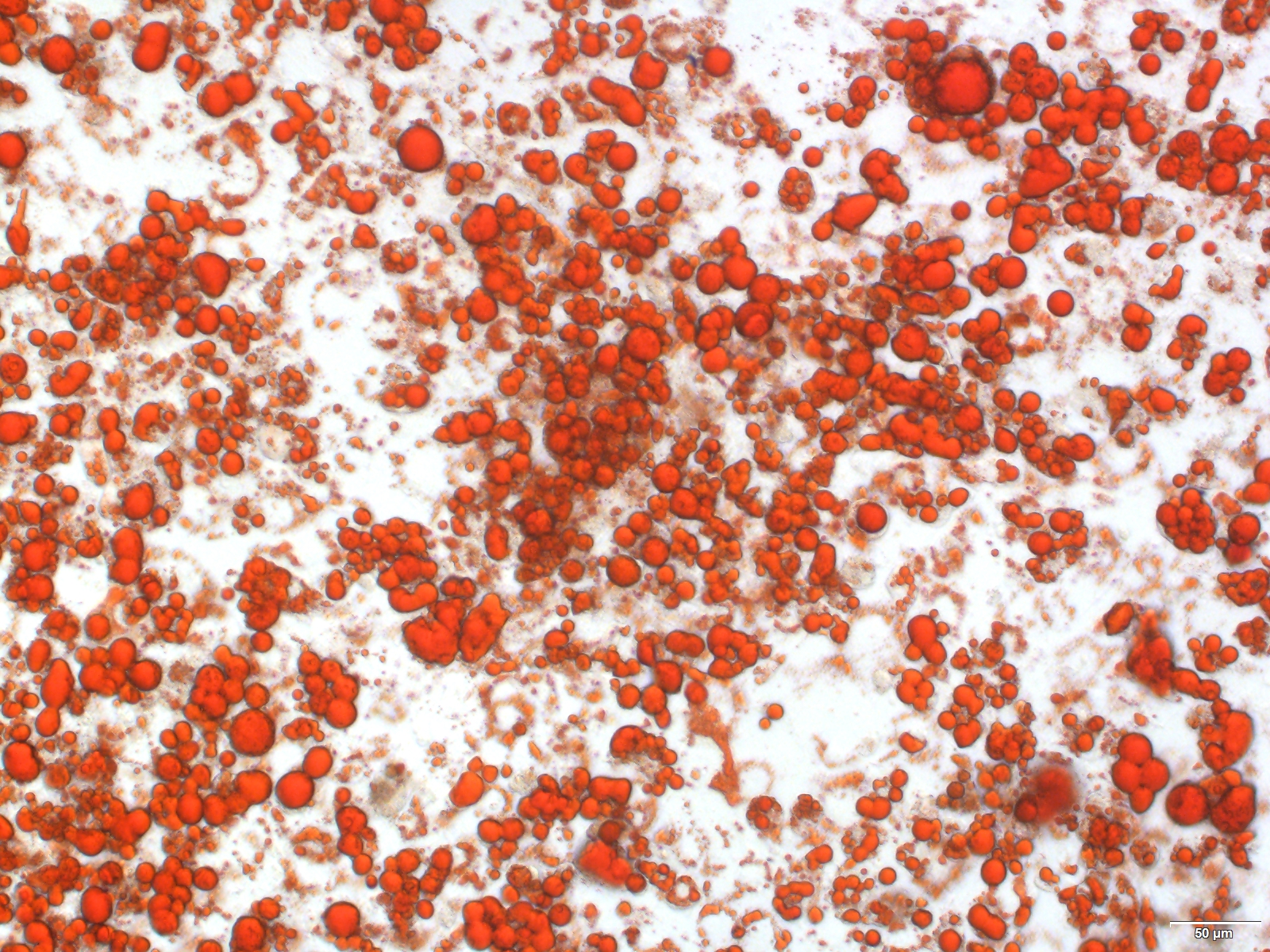

Supplement: Supplementary file 13 — Source data Fig. 7 [file 44321_2025_335_MOESM13_ESM.zip › Figure 7/Figure 7-D/Oil Red O staining/siGnas-20x.jpg]

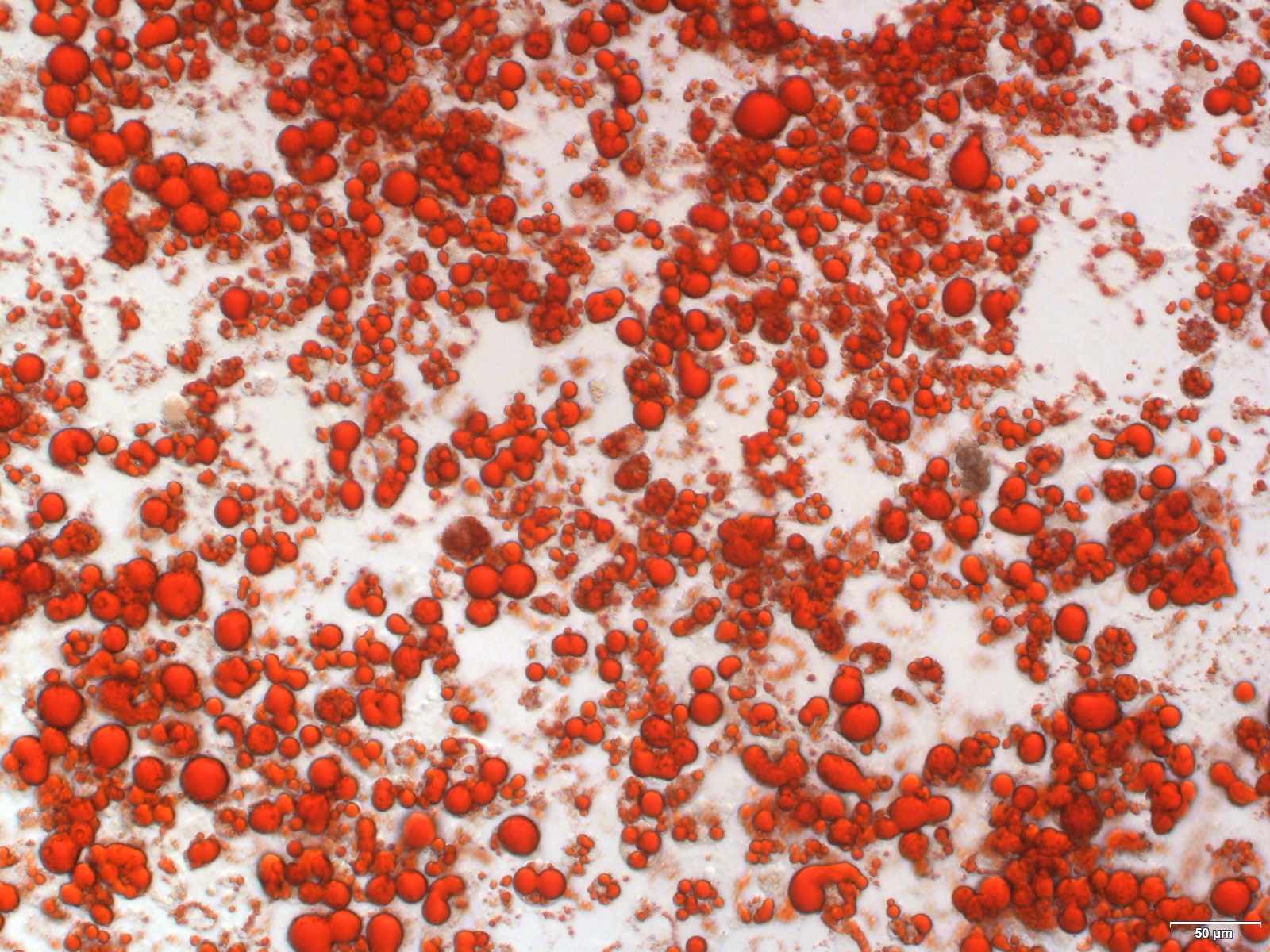

Supplement: Supplementary file 13 — Source data Fig. 7 [file 44321_2025_335_MOESM13_ESM.zip › Figure 7/Figure 7-D/Oil Red O staining/ISM+siGnas-20x.jpg]

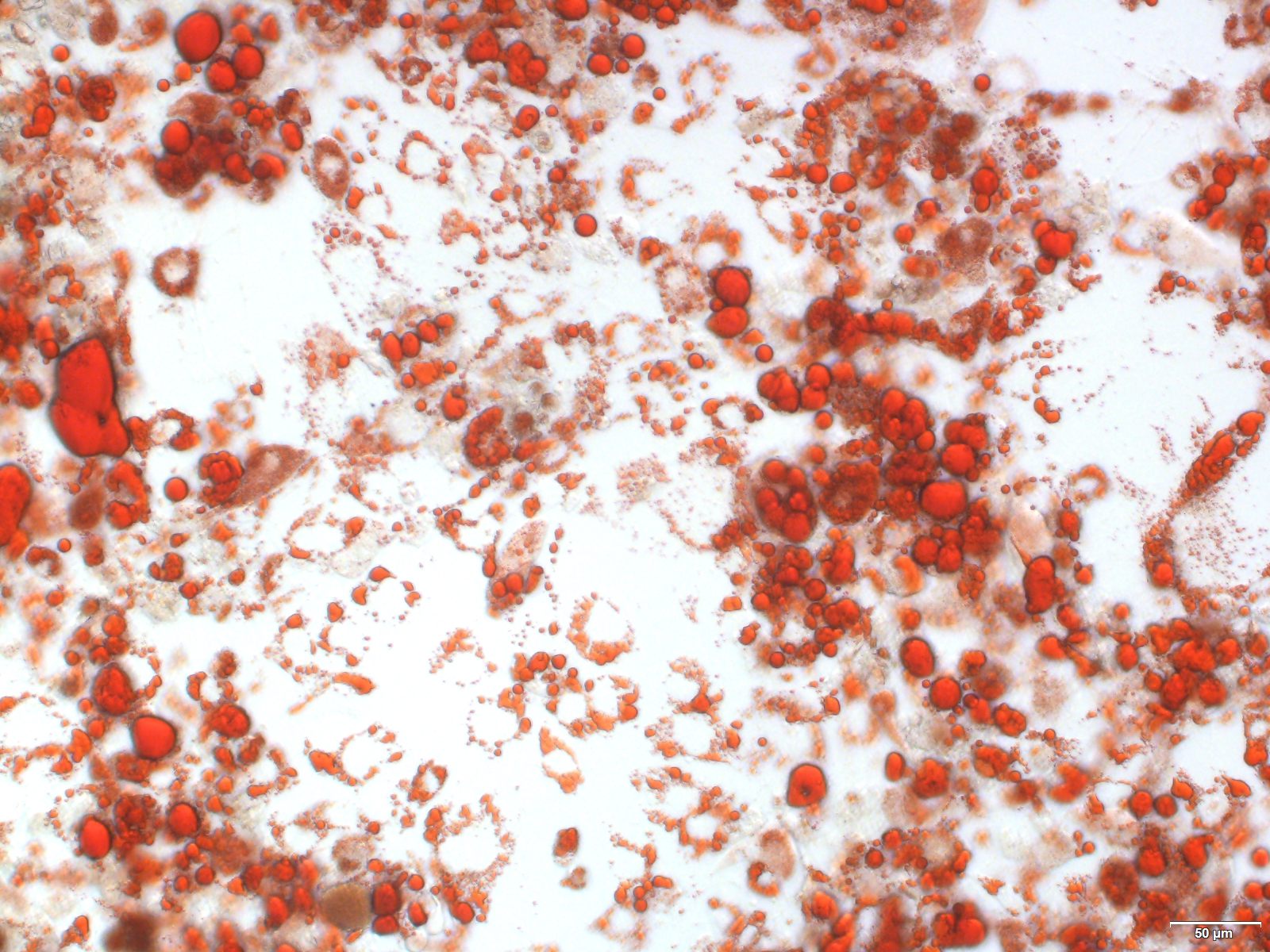

Supplement: Supplementary file 13 — Source data Fig. 7 [file 44321_2025_335_MOESM13_ESM.zip › Figure 7/Figure 7-D/Oil Red O staining/ISM-20X.jpg]

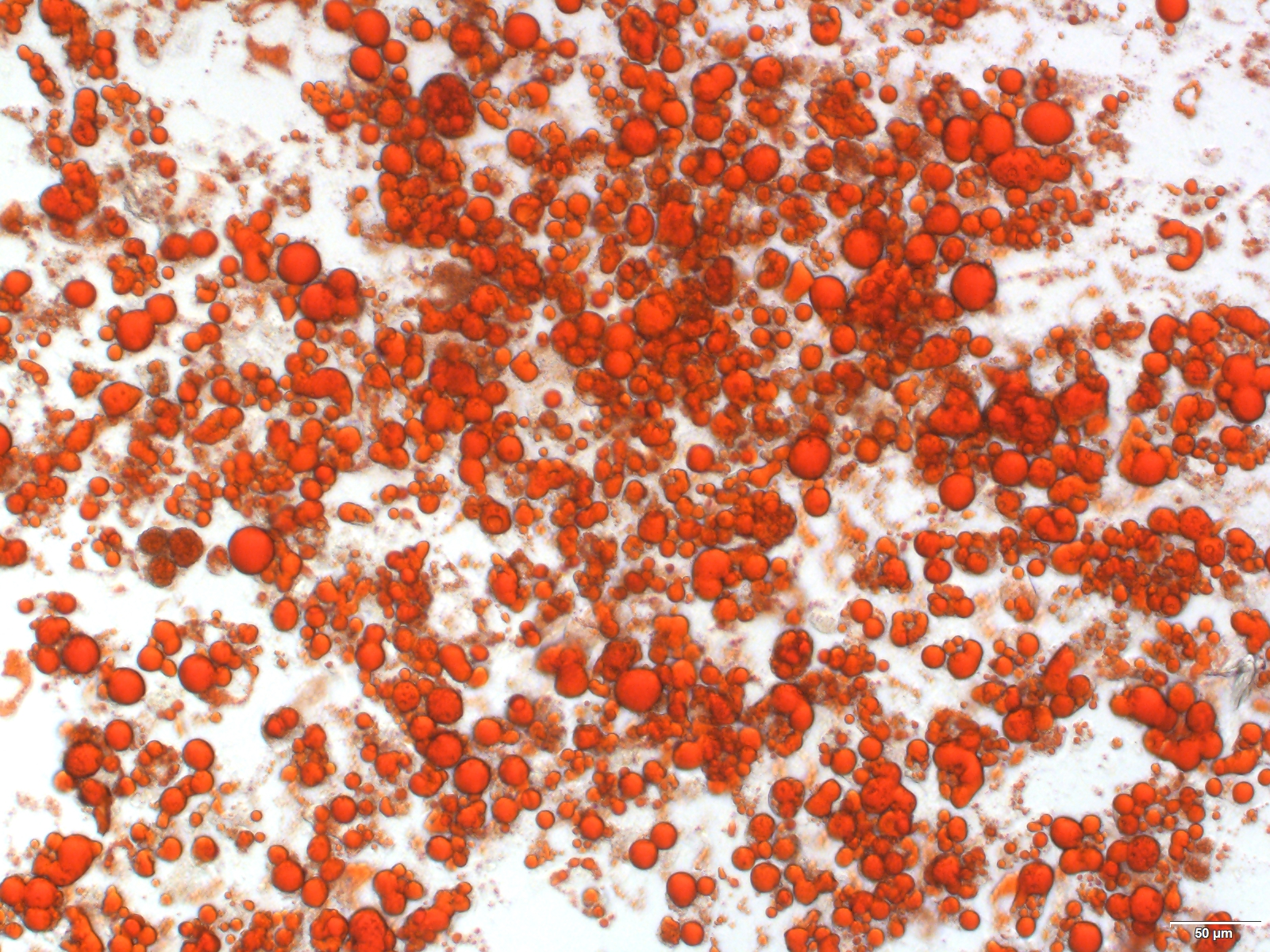

Supplement: Supplementary file 13 — Source data Fig. 7 [file 44321_2025_335_MOESM13_ESM.zip › Figure 7/Figure 7-D/Oil Red O staining/Control-20x.jpg]
