# Supplementary material for: Isomeranzin activates Gnas-AMPK signaling to drive white adipose browning and curb obesity in mice
Source: EMBO Mol Med. 2025 Nov 26;18(1):55–90. doi: 10.1038/s44321-025-00335-y (PMC12808274; doi:10.1038/s44321-025-00335-y)
Supplement: Supplementary file 14 — Expanded View Figures [file 44321_2025_335_MOESM14_ESM.pdf]

# Expanded View Figures

**Figure EV1. Identification of small-molecule compounds promoting adipocyte browning using CMap.**

(**A**) Volcano plot of differentially expressed genes in the RNA-seq dataset GSE164219 from cold exposure-induced browning of iWAT in mice ($n = 3$). Statistical test: Moderated t-tests and Benjamini-Hochberg procedure. (**B**) Volcano plot of differentially expressed genes in the RNA-seq dataset GSE133619 from cold exposure-induced browning of iWAT in mice ($n = 3$). Statistical test: Moderated t-tests and Benjamini-Hochberg procedure. (**C**) Volcano plot of differentially expressed genes in the RNA-seq dataset GSE129083 from β-adrenergic agonist CL316,243-induced browning of iWAT in mice ($n = 4$). Statistical test: Moderated t-tests and Benjamini-Hochberg procedure. (**D**) Volcano plot of differentially expressed genes in the RNA-seq dataset GSE98132 from β-adrenergic agonist CL316,243-induced browning of iWAT in mice ($n = 3$). Statistical test: Moderated t-tests and Benjamini-Hochberg procedure. (**E**) Schematic illustration of the treatment method for maturation and differentiation induction in primary inguinal adipocytes, along with the administration of candidate compounds (10 μM) screened from CMap. (**F**) Quantitative analysis of relative protein expression levels of Pgc1α in mature primary inguinal adipocytes from control and ISM (50 μM) treated groups ($n = 3$). Statistical test: Unpaired Student's two-tailed t-tests. $p < 0.0001$. (**G**) Quantitative analysis of OCR in mature primary inguinal adipocytes from control and ISM (50 μM) treated groups in Fig. 2O ($n = 3$). Statistical test: Unpaired Student's two-tailed t-tests. Basal respiration, $p = 0.0142$; Proton leak, $p = 0.0299$; Maximal respiration, $p = 0.0100$; Spare respiratory capacity, $p = 0.0043$; Non-mitochondrial respiration, $p = 0.8941$; ATP production, $p = 0.3578$. (**H**) Schematic illustration of the treatment method for adipogenesis in adipocytes with ISM. (**I**) Relative mRNA expression levels of Pparγ, C/Ebpα, and Fabp4 in mature primary inguinal adipocytes treated with different concentrations of ISM ($n = 3$). Statistical test: one-way ANOVA followed by Dunnett's multiple comparisons test. Pparγ: 0 μM vs. 2 μM, $p = 0.6106$; 0 μM vs. 10 μM, $p = 0.6716$; 0 μM vs. 50 μM, $p = 0.7857$; C/Ebpα: 0 μM vs. 2 μM, $p = 0.2238$; 0 μM vs. 10 μM, $p = 0.2367$; 0 μM vs. 50 μM, $p = 0.1733$; Fabp4: 0 μM vs. 2 μM, p = 0.8648;0 μM vs. 10 μM, $p = 0.3654$; 0 μM vs. 50 μM, $p = 0.9361$. (**J**) Quantitative analysis of relative protein expression levels of Pparγ, C/Ebpα, and Fabp4 in mature primary inguinal adipocytes treated with varying concentrations of ISM ($n = 3$). Statistical test: one-way ANOVA followed by Dunnett's multiple comparisons test. Pparγ: 0 μM vs. 2 μM, $p = 0.3690$; 0 μM vs. 10 μM, $p = 0.9934$; 0 μM vs. 50 μM, $p = 0.5491$; C/Ebpα: 0 μM vs. 2 μM, $p = 0.2496$; 0 μM vs. 10 μM, $p = 0.4520$; 0 μM vs. 50 μM, $p = 0.8255$; Fabp4: 0 μM vs. 2 μM, $p = 0.6430$; 0 μM vs. 10 μM, $p = 0.8337$; 0 μM vs. 50 μM, $p = 0.9254$. $n$ presents biological replicates. Data are presented as mean ± SEM. Statistical significance is indicated as follows: $^{*}p < 0.05$, $^{**}p < 0.01$, $^{***}p < 0.001$, $^{****}p < 0.0001$, and ns indicates no significant difference.

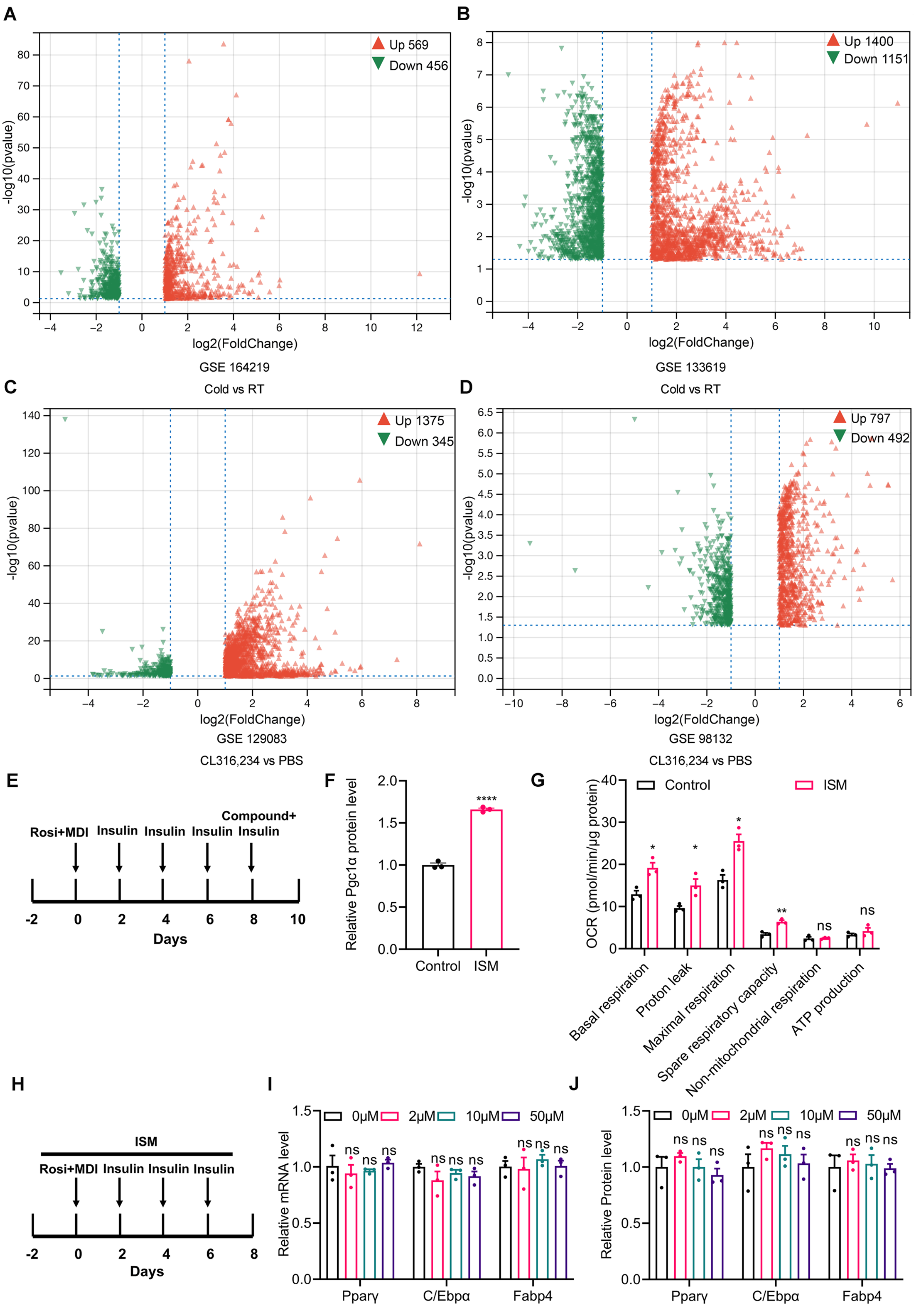
A
Up 569
Down 456
-log10(pvalue)
log2(FoldChange)
GSE 164219
Cold vs RT
B
Up 1400
Down 1151
-log10(pvalue)
log2(FoldChange)
GSE 133619
Cold vs RT
C
Up 1375
Down 345
-log10(pvalue)
log2(FoldChange)
GSE 129083
CL316,234 vs PBS
D
Up 797
Down 492
-log10(pvalue)
log2(FoldChange)
GSE 98132
CL316,234 vs PBS
E
Rosi+MDI Insulin Insulin Insulin Compound+ Insulin
-2 0 2 4 6 8 10
Days
F
Relative Pgc1α protein level
Control ISM
****
G
Control ISM
OCR (pmol/min/μg protein)
Basal respiration
Proton leak
Maximal respiration
Spare respiratory capacity
Non-mitochondrial respiration
ATP production
H
ISM
Rosi+MDI Insulin Insulin Insulin
-2 0 2 4 6 8
Days
I
0μM 2μM 10μM 50μM
Relative mRNA level
Pparγ C/Ebpα Fabp4
J
0μM 2μM 10μM 50μM
Relative Protein level
Pparγ C/Ebpα Fabp4

 © The Author(s)

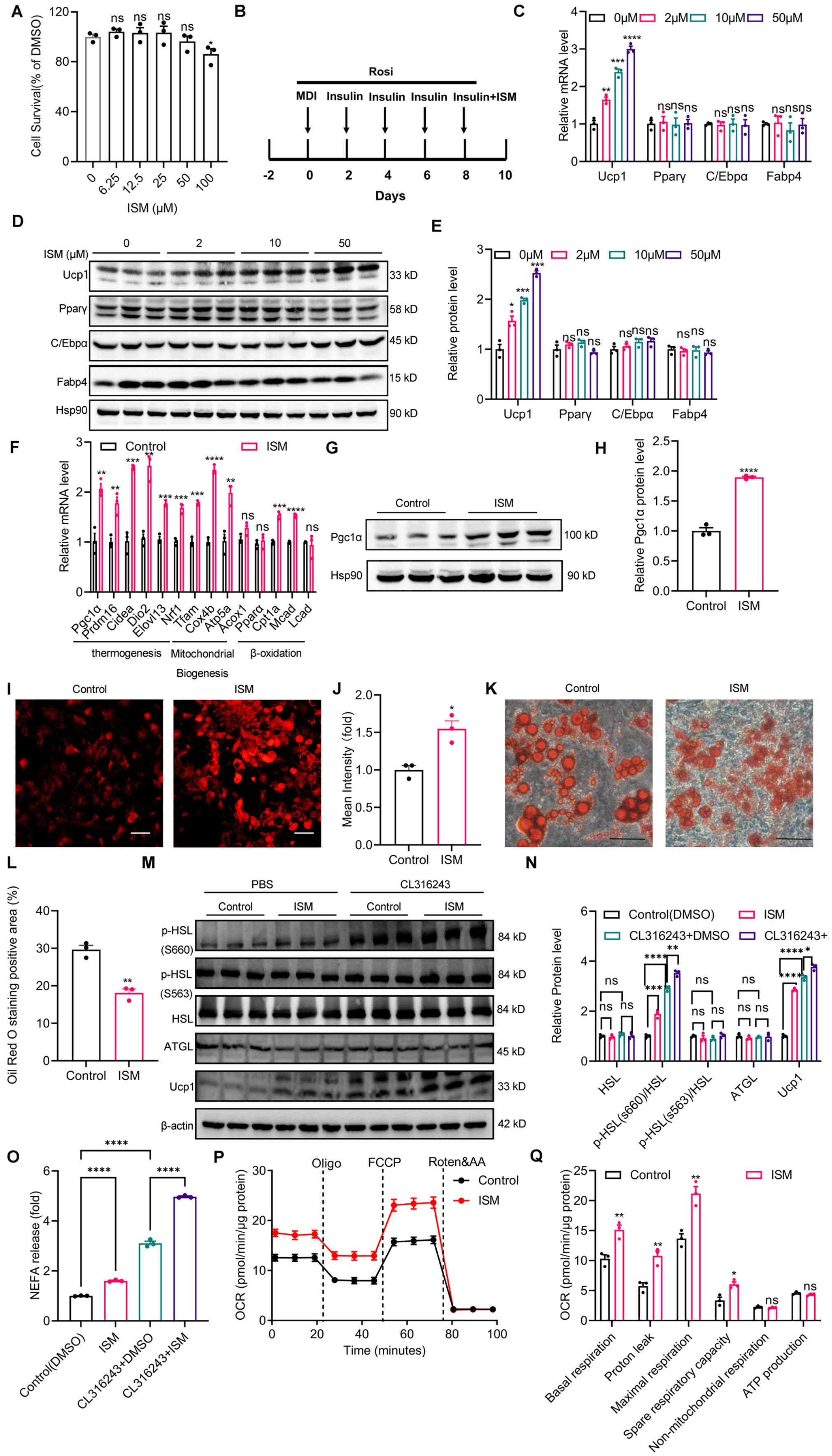
A
B
C
D
E
F
G
H
I
J
K
L
M
N
O
P
Q
Rosi
MDI Insulin Insulin Insulin Insulin+ISM
Days
ISM (μM)
Cell Survival(% of DMSO)
Relative mRNA level
Relative protein level
0μM 2μM 10μM 50μM
Ucp1 Pparγ C/Ebpα Fabp4 Hsp90
Control ISM
Pgc1α
Relative Pgc1α protein level
thermogenesis
Mitochondrial Biogenesis
β-oxidation
Mean Intensity (fold)
Oil Red O staining positive area (%)
PBS CL316243
p-HSL (S660)
p-HSL (S563)
HSL
ATGL
β-actin
Relative Protein level
Control(DMSO) ISM CL316243+DMSO CL316243+
NEFA release (fold)
Oligo FCCP Roten&AA
OCR (pmol/min/μg protein)
Time (minutes)
Basal respiration
Proton leak
Maximal respiration
Spare respiratory capacity
Non-mitochondrial respiration
ATP production

**Figure EV2. Validation of ISM as a Novel Thermogenic Inducer.**

(**A**) CCK8 assay measuring cell viability in human mesenchymal stem cells (HMSC) treated with different concentrations of ISM ($n = 3$). Statistical test: one-way ANOVA followed by Dunnett's multiple comparisons test.0 vs. 6.25, $p = 0.9070$; 0 vs. 12.5, $p = 0.9608$; 0 vs. 25, $p = 0.9549$; 0 vs. 50, $p = 0.9422$; 0 vs. 100, $p = 0.0420$. (**B**) Schematic illustration of the treatment method for maturation and differentiation induction in HMSC and the promotion of browning in mature human adipocytes by ISM. (**C**) Relative mRNA expression levels of Ucp1, Pparγ, C/Ebpα, and Fabp4 in mature human adipocytes derived from HMSC treated with different concentrations of ISM ($n = 3$). Statistical test: one-way ANOVA followed by Dunnett's multiple comparisons test. Ucp1: 0 μM vs. 2 μM, $p = 0.0042$; 0 μM vs. 10 μM, $p = 0.0003$; 0 μM vs. 50 μM, $p < 0.0001$; Pparγ: 0 μM vs. 2 μM, $p = 0.3909$; 0 μM vs. 10 μM, $p = 0.9130$; 0 μM vs. 50 μM, p = 0.8935; C/Ebpα: 0 μM vs. 2 μM, $p = 0.6652$; 0 μM vs. 10 μM, $p = 0.6192$; 0 μM vs. 50 μM, $p = 0.8497$; Fabp4: 0 μM vs. 2 μM, $p = 0.8454$; 0 μM vs. 10 μM, $p = 0.4445$; 0 μM vs. 50 μM, $p = 0.9231$. (**D**) Representative immunoblot images of Ucp1, Pparγ, C/Ebpα, and Fabp4 in mature human adipocytes derived from HMSCs treated with varying concentrations of ISM. (**E**) Quantitative analysis of relative protein expression levels of Ucp1, Pparγ, C/Ebpα, and Fabp4 in mature human adipocytes derived from HMSCs treated with varying concentrations of ISM ($n = 3$). Statistical test: one-way ANOVA followed by Dunnett's multiple comparisons test. Ucp1: 0 μM vs. 2 μM, $p = 0.0134$; 0 μM vs. 10 μM, $p = 0.0007$; 0 μM vs. 50 μM, $p = 0.0001$; Pparγ: 0 μM vs. 2 μM, $p = 0.3452$; 0 μM vs. 10 μM, $p = 0.2204$; 0 μM vs. 50 μM, $p = 0.5461$; C/Ebpα: 0 μM vs. 2 μM, $p = 0.4425$; 0 μM vs. 10 μM, $p = 0.1332$; 0 μM vs. 50 μM, $p = 0.1133$; Fabp4: 0 μM vs. 2 μM, $p = 0.6264$; 0 μM vs. 10 μM, $p = 0.8377$; 0 μM vs. 50 μM, $p = 0.3627$. (**F**) Relative mRNA expression levels of thermogenesis, mitochondrial biogenesis and β-oxidation related genes in mature human adipocytes derived from HMSCs from control and ISM (50 μM) treated groups ($n = 3$). Statistical test: Unpaired Student's two-tailed t-tests. Pgc1α, $p = 0.0045$; Prdm16, $p = 0.0060$; Cidea, $p = 0.0004$; Dio2, $p = 0.0013$; Elovl13, $p = 0.0004$; Nrf1, $p = 0.0008$; Tfam, $p = 0.4859$; Cox4b, $p = 0.0004$; Atp5a, $p = 0.0067$; Acox1, $p = 0.0745$; Pparα, $p = 0.4859$; Cpt1a, $p = 0.0004$; Mcad, $p < 0.0001$; Lcad, $p = 0.6934$. (**G**) Representative immunoblot images of Pgc1α in mature human adipocytes derived from HMSC in control and ISM (50 μM) treatment groups. (**H**) Quantitative analysis of relative protein expression levels of Pgc1α in mature human adipocytes derived from HMSC in control and ISM (50 μM) treatment groups ($n = 3$). Statistical test: Unpaired Student's two-tailed t-tests. $p < 0.0001$; (**I**) Representative MitoTracker staining images of mature human adipocytes derived from HMSC from control and ISM (50 μM) treated groups; scale bar = 50 μm. (**J**) Quantitative analysis of MitoTracker staining images in mature primary inguinal adipocytes from control and ISM (50 μM) treated groups ($n = 3$). Statistical test: Unpaired Student's two-tailed t-tests. $p = 0.0105$. (**K**) Representative Oil Red O staining images of mature human adipocytes derived from HMSC from control and ISM (50 μM) treated groups; scale bar = 50 μm. (**L**) Quantitative analysis of Oil Red O staining images in mature human adipocytes derived from HMSC from control and ISM (50 μM) treated groups ($n = 3$). Statistical test: Unpaired Student's two-tailed t-tests. $p = 0.0017$. After 24 h of ISM treatment, the cells were stimulated with 1 μM CL316243 for an additional 24 h. (**M**) Representative immunoblot images of p-HSL(Ser660), p-HSL(Ser563), HSL, ATGL and Ucp1 in mature human adipocytes derived from HMSC from different groups. (**N**) Quantitative analysis of relative protein expression levels of p-HSL(Ser660), p-HSL (Ser563), HSL, ATGL and Ucp1 in mature human adipocytes derived from HMSC from different groups ($n = 3$). Statistical test: one-way ANOVA followed by Tukey's multiple comparisons. HSL: Control (DMSO) vs. ISM, $p = 0.5801$; Control (DMSO) vs. CL316243 + DMSO, $p = 0.2296$; CL316243 + DMSO vs. CL316243 + ISM, $p = 0.4517$; p-HSL(S660)/HSL: Control (DMSO) vs. ISM, $p = 0.0009$; Control (DMSO) vs. CL316243 + DMSO, $p < 0.0001$; CL316243 + DMSO vs. CL316243 + ISM, $p = 0.0032$; p-HSL(S563)/HSL: Control (DMSO) vs. ISM, $p = 0.5010$; Control (DMSO) vs. CL316243 + DMSO, $p = 0.1447$; CL316243 + DMSO vs. CL316243 + ISM, $p = 0.1580$; ATGL: Control (DMSO) vs. ISM, $p = 0.3646$; Control (DMSO) vs. CL316243 + DMSO, $p = 0.5620$; CL316243 + DMSO vs. CL316243 + ISM, $p = 0.8974$; Ucp1: Control (DMSO) vs. ISM, $p < 0.0001$; Control (DMSO) vs. CL316243 + DMSO, $p < 0.0001$; CL316243 + DMSO vs. CL316243 + ISM, $p = 0.0146$. (**O**) NEFA levels in the mature human adipocytes derived from HMSC culture medium from different groups ($n = 3$). Statistical test: one-way ANOVA followed by Tukey's multiple comparisons. Control (DMSO) vs. ISM, $p < 0.0001$; Control (DMSO) vs. CL316243 + DMSO, $p < 0.0001$; CL316243 + DMSO vs. CL316243 + ISM, $p < 0.0001$. (**P**) Oxygen consumption rates (OCR) in mature human adipocytes derived from HMSC from control and ISM (50 μM) treated groups ($n = 3$). (**Q**) Quantitative analysis of OCR in mature human adipocytes derived HMSC from control and ISM (50 μM) treated groups ($n = 3$). Statistical test: Unpaired Student's two-tailed t-tests. Basal respiration, $p = 0.0095$; Proton leak, $p = 0.0070$; Maximal respiration, $p = 0.0058$; Spare respiratory capacity, $p = 0.0147$; Non-mitochondrial respiration, $p = 0.5466$; ATP production, $p = 0.3115$. *n* presents biological replicates. Data are presented as mean ± SEM. Statistical significance is indicated as follows: *$p < 0.05$, **$p < 0.01$, ***$p < 0.001$, ****$p < 0.0001$, and ns indicates no significant difference.

 © The Author(s)

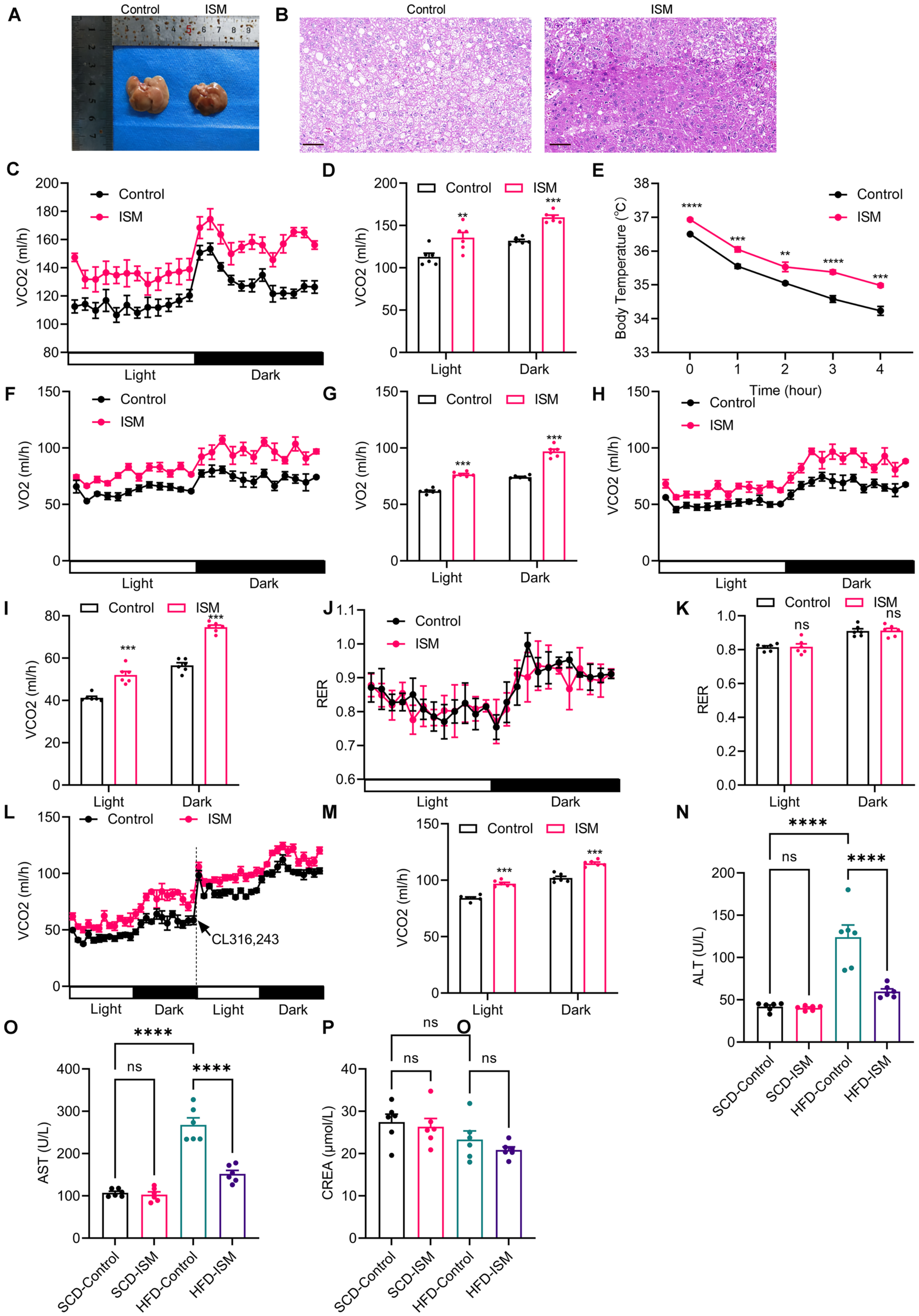
A
Control
ISM
B
Control
ISM
C
VCO2 (ml/h)
Control
ISM
Light
Dark
D
VCO2 (ml/h)
Control
ISM
Light
Dark
E
Body Temperature (°C)
Control
ISM
Time (hour)
F
VO2 (ml/h)
Control
ISM
Light
Dark
G
VO2 (ml/h)
Control
ISM
Light
Dark
H
VCO2 (ml/h)
Control
ISM
Light
Dark
I
VCO2 (ml/h)
Control
ISM
Light
Dark
J
RER
Control
ISM
Light
Dark
K
RER
Control
ISM
ns
Light
Dark
L
VCO2 (ml/h)
Control
ISM
CL316,243
Light
Dark
Light
Dark
M
VCO2 (ml/h)
Control
ISM
Light
Dark
N
ALT (U/L)
ns
SCD-Control
SCD-ISM
HFD-Control
HFD-ISM
O
AST (U/L)
ns
SCD-Control
SCD-ISM
HFD-Control
HFD-ISM
P
CREA (μmol/L)
ns
O
SCD-Control
SCD-ISM
HFD-Control
HFD-ISM

**Figure EV3. ISM protects against obesity induced by high-fat diet and improves obesity-related metabolic dysfunction through promoting thermogenesis in mice.**

(**A**) Representative images of the general appearance of livers from HFD mice. (**B**) Representative H&E staining images of liver sections; scale bar = 50 μm. (**C**) $VCO_2$ of HFD mice. (**D**) Quantitative analysis of $VCO_2$ in (**C**). Statistical test: ANCOVA, with body weight as a covariate. $VCO_2$-Light, $p = 0.004$; $VCO_2$-Dark, $p < 0.001$. (**E**) Rectal temperature of HFD mice under different treatments measured at specified times at 4 °C ($n = 6$). Statistical test: Unpaired Student's two-tailed t-tests. 0 h, $p < 0.0001$; 1 h, $p = 0.0005$; 2 h, $p = 0.0075$; 3 h, $p < 0.0001$; 4 h, $p = 0.0003$. (**F**, **G**) $VO_2$ of mice injected with either vehicle or ISM and fed a high-fat diet for 4 weeks ($n = 6$). Statistical test: ANCOVA, with body weight as a covariate.$VO_2$-Light, $p < 0.001$; $VO_2$-Dark, $p < 0.001$. (**H**, **I**) $VCO_2$ of mice injected with either vehicle or ISM and fed a high-fat diet for 4 weeks ($n = 6$). Statistical test: ANCOVA, with body weight as a covariate. $VCO_2$-Light, $p < 0.001$; $VCO_2$-Dark, $p < 0.001$. (**J**, **K**) Respiratory exchange ratio (RER) of mice injected with either vehicle or ISM and fed a high-fat diet for 4 weeks ($n = 6$). Statistical test: Unpaired Student's two-tailed t-tests. RER-Light, $p = 0.9647$; RER-Dark, $p = 0.3803$. (**L**) $VCO_2$ of mice treated with CL316243. (**M**) Quantitative analysis of $VCO_2$ in mice after CL316243 treatment ($n = 6$). Statistical test: ANCOVA, with body weight as a covariate. $VCO_2$-Light, $p < 0.001$; $VCO_2$-Dark, $p < 0.001$. (**N**) Quantitative analysis of serum ALT levels in mice from different groups ($n = 6$). Statistical test: one-way ANOVA followed by Tukey's multiple comparisons. SCD-Control vs. SCD-ISM, $p = 0.9989$; SCD-Control vs. HFD-Control, $p < 0.0001$; HFD-Control vs. HFD-ISM, $p < 0.0001$. (**O**) Quantitative analysis of serum AST levels in mice from different groups ($n = 6$). Statistical test: one-way ANOVA followed by Tukey's multiple comparisons. SCD-Control vs. SCD-ISM, $p = 0.9903$; SCD-Control vs. HFD-Control, $p < 0.0001$; HFD-Control vs. HFD-ISM, $p < 0.0001$. (**P**) Quantitative analysis of serum CREA levels in mice from different groups ($n = 6$). Statistical test: one-way ANOVA followed by Tukey's multiple comparisons. SCD-Control vs. SCD-ISM, $p = 0.9681$; SCD-Control vs. HFD-Control, $p = 0.3478$; HFD-Control vs. HFD-ISM, $p = 0.7391$. *n* presents biological replicates. Data are presented as mean ± SEM. Statistical significance is indicated as follows: *$p < 0.05$, **$p < 0.01$, ***$p < 0.001$, ****$p < 0.0001$, and ns indicates no significant difference.


© The Author(s)

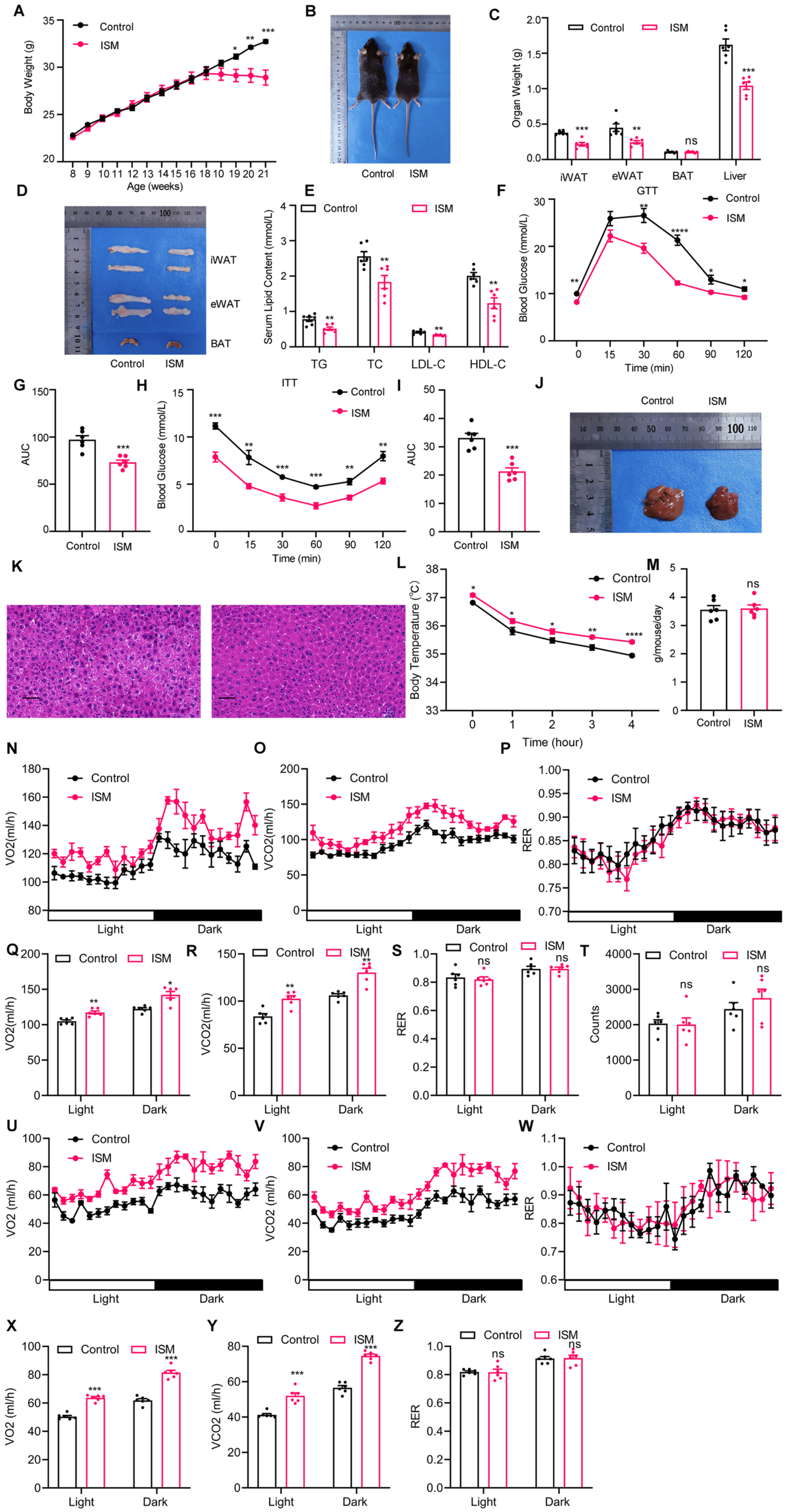
A
Body Weight (g)
Age (weeks)
Control
ISM
B
Control ISM
C
Organ Weight (g)
Control
ISM
iWAT
eWAT
BAT
Liver
D
iWAT
eWAT
BAT
Control ISM
E
Serum Lipid Content (mmol/L)
TG
TC
LDL-C
HDL-C
F
GTT
Blood Glucose (mmol/L)
Time (min)
G
AUC
H
ITT
Blood Glucose (mmol/L)
Time (min)
I
AUC
J
Control ISM
K
L
Body Temperature (°C)
Time (hour)
M
g/mouse/day
N
VO2(ml/h)
Light
Dark
O
VCO2(ml/h)
P
RER
Q
R
S
T
Counts
U
VO2 (ml/h)
V
VCO2 (ml/h)
W
X
Y
Z

**Figure EV4. Administration of ISM also improves glucose tolerance and insulin sensitivity in mice fed a standard chow diet (SCD).**

Mice were intraperitoneally injected with either vehicle or ISM and fed a standard chow diet for 13 weeks ($n = 6$). *n* presents biological replicates. (**A**) Body weight of mice in different groups after feeding on a standard chow diet. 19w, $p = 0.0173$; 20w, $p = 0.0025$; 21w, $p = 0.0009$. (**B**) Representative images of the general appearance of mice prior to euthanasia. (**C**) Weights of tissue organs (inguinal subcutaneous adipose tissue, epididymal visceral adipose tissue, brown adipose tissue, and liver) from mice. iWAT, $p = 0.0001$; eWAT, $p = 0.0058$; BAT, $p = 0.9624$; Liver, $p = 0.0002$. (**D**) Representative images of the general appearance of adipose tissues (iWAT, eWAT, and BAT) from mice. (**E**) Serum lipid content (triglycerides, total cholesterol, low-density lipoprotein cholesterol, high-density lipoprotein cholesterol) after feeding on a standard chow diet for 13 weeks. TG, $p = 0.0059$; TC, $p = 0.0090$; LDL-C, $p = 0.0037$; HDL-C, $p = 0.0015$. (**F**) Glucose tolerance test (GTT) after feeding on SCD for 11 weeks. 0 min, $p = 0.0023$; 15 min, $p = 0.0881$; 30 min, $p = 0.0034$; 60 min, $p < 0.0001$; 90 min, $p = 0.0205$; 120 min, $p = 0.0134$. (**G**) Quantitative analysis of the glucose tolerance test (GTT). $p = 0.0005$. (**H**) Insulin tolerance test (ITT) after feeding on SCD for 12 weeks. 0 min, $p = 0.0003$; 15 min, $p = 0.0033$; 30 min, $p = 0.0008$; 60 min, $p = 0.0002$; 90 min, $p = 0.0021$; 120 min, $p = 0.0010$. (**I**) Quantitative analysis of the insulin tolerance test (ITT). $p = 0.0002$. (**J**) Representative images of the general appearance of livers from SCD mice. (**K**) Representative H&E staining images of liver sections; scale bar = 50 μm. (**L**) Rectal temperature of SCD mice under different treatments measured at specified times at 4 °C. 0 h, $p = 0.0267$; 1 h, $p = 0.0426$; 2 h, $p = 0.0324$; 3 h, $p = 0.0059$; 4 h, $p < 0.0001$. (**M**) Food intake in mice. $p = 0.8306$. (**N–P**) $VO_2$, $VCO_2$ and Respiratory exchange ratio (RER) of SCD mice. (**Q–S**) Quantitative analysis of $VO_2$, $VCO_2$ and RER of SCD mice in (**N–P**). $VO_2$-Light, $p = 0.007$; $VO_2$-Dark, $p = 0.018$; $VCO_2$-Light, $p = 0.001$; $VCO_2$-Dark, $p = 0.001$; RER-Light, $p = 0.6621$; RER-Dark, $p = 0.9868$. (**T**) Mean physical activity in mice. Light, $p = 0.9077$; Dark, $p = 0.3431$. (**U–W**) $VO_2$, $VCO_2$ and RER of mice injected with either vehicle or ISM and fed a standard chow diet for 4 weeks ($n = 6$). *n* presents biological replicates. (**X–Z**) Quantitative analysis of $VO_2$, $VCO_2$ and RER of SCD mice in (**U–W**) ($n = 6$). $VO_2$-Light, $p < 0.001$; $VO_2$-Dark, $p < 0.001$; $VCO_2$-Light, $p < 0.001$; $VCO_2$-Dark, $p < 0.001$; RER-Light, $p = 0.8021$; RER-Dark, $p = 0.7714$. Data are presented as mean ± SEM. Statistical test: The energy metabolism data, including $VO_2$ and $VCO_2$ were analyzed by ANCOVA, with body weight as a covariate. The others: Unpaired Student's two-tailed t-tests. Statistical significance is indicated as follows: *$p < 0.05$, **$p < 0.01$, ***$p < 0.001$, ****$p < 0.0001$, and ns indicates no significant difference.


© The Author(s)

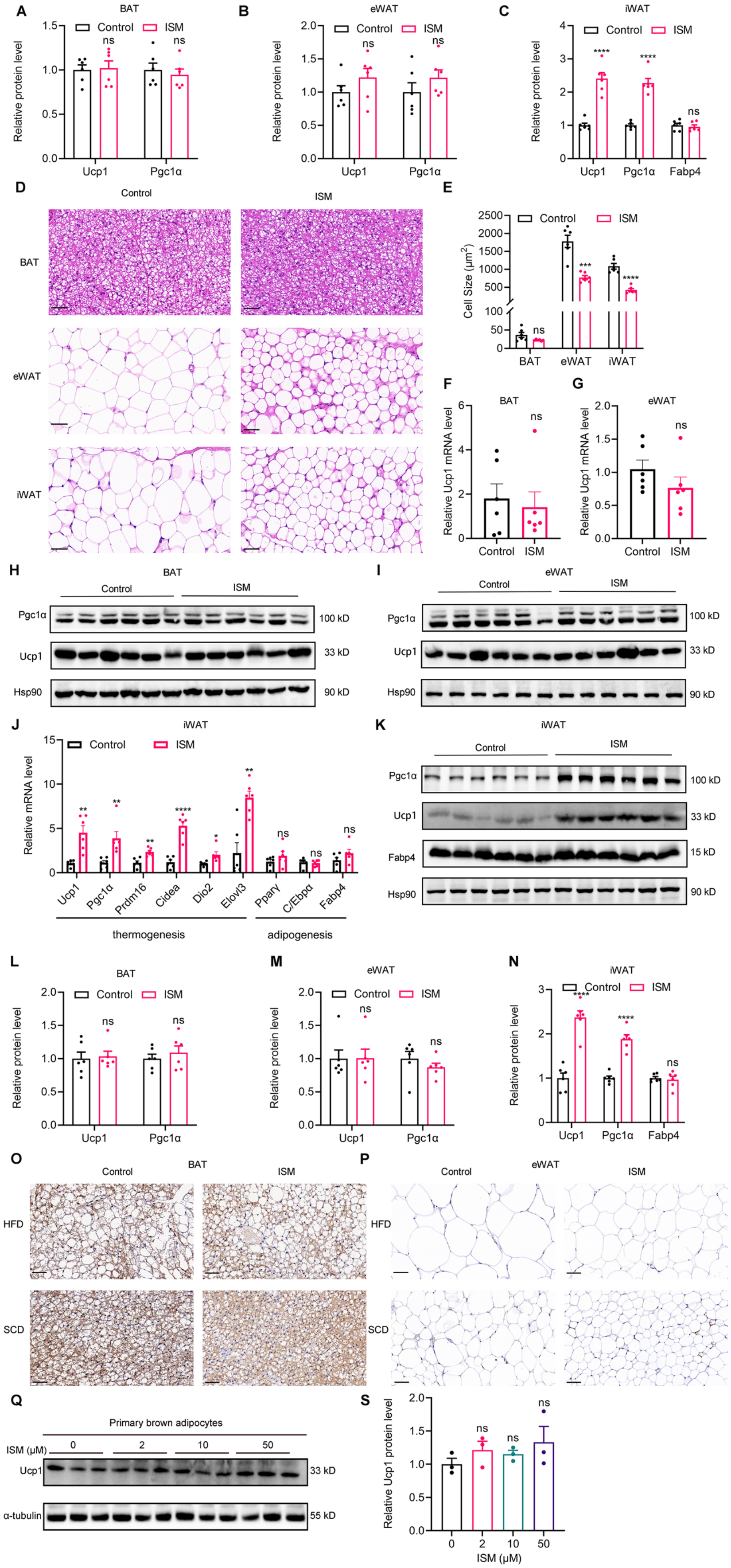
A
BAT
Control
ISM
Relative protein level
Ucp1
Pgc1α
B
eWAT
C
iWAT
Fabp4
D
E
Cell Size (μm²)
eWAT
F
Relative Ucp1 mRNA level
G
H
I
J
Relative mRNA level
Ucp1
Pgc1α
Prdm16
Cidea
Dio2
Elovl3
Pparγ
C/Ebpα
Fabp4
thermogenesis
adipogenesis
K
Hsp90
100 kD
33 kD
90 kD
15 kD
L
M
N
O
HFD
SCD
P
Q
Primary brown adipocytes
ISM (μM)
α-tubulin
55 kD
S
Relative Ucp1 protein level
ISM (μM)

**Figure EV5. ISM administration also promotes subcutaneous adipose browning and increases thermogenesis in mice fed a standard chow diet (SCD).**

(**A**) Quantitative analysis of relative protein expression levels for the proteins shown in Fig. 4E ($n = 6$). Ucp1, $p = 0.8490$; Pgc1α, $p = 0.6006$. (**B**) Quantitative analysis of relative protein expression levels for the proteins shown in Fig. 4F ($n = 6$). Ucp1, $p = 0.2182$; Pgc1α, $p = 0.2610$. (**C**) Quantitative analysis of relative protein expression levels for the proteins shown in Fig. 4H ($n = 6$). Ucp1, $p < 0.0001$; Pgc1α, $p < 0.0001$; Fabp4, $p = 0.6071$. (**D**) Representative H&E staining images of BAT, eWAT, and iWAT sections; scale bar = 50 µm. (**E**) Quantitative analysis of adipocyte size in BAT, eWAT, and iWAT ($n = 6$). BAT, $p = 0.0708$; eWAT, $p = 0.0002$; iWAT, $p < 0.0001$. (**F**) Relative mRNA expression levels of Ucp1 in BAT ($n = 6$). $p = 0.6939$. (**G**) Relative mRNA expression levels of Ucp1 in eWAT ($n = 6$). $p = 0.2254$. (**H**) Representative immunoblot images of Pgc1α and Ucp1 in BAT. (**I**) Representative immunoblot images of Pgc1α and Ucp1 in eWAT. (**J**) Relative mRNA expression levels of thermogenesis and adipogenesis related genes in iWAT ($n = 6$). Ucp1, $p = 0.0015$; Pgc1α, $p = 0.0063$; Prdm16, $p = 0.0016$; Cidea, $p < 0.0001$; Dio2, $p = 0.0150$; Elovl3, $p = 0.0010$; Pparγ, $p = 0.2402$; C/Ebpα, $p = 0.7316$; Fabp4, $p = 0.1663$. (**K**) Representative immunoblot images of Pgc1α, Ucp1, and Fabp4 in iWAT. (**L**) Quantitative analysis of relative protein expression levels for the proteins shown in (**H**) ($n = 6$). Ucp1, $p = 0.8064$; Pgc1α, $p = 0.4848$. (**M**) Quantitative analysis of relative protein expression levels for the proteins shown in (**I**) ($n = 6$). Ucp1, $p = 0.9714$; Pgc1α, $p = 0.3209$. (**N**) Quantitative analysis of relative protein expression levels for the proteins shown in (**K**) ($n = 6$). Ucp1, $p < 0.0001$; Pgc1α, $p < 0.0001$; Fabp4, $p = 0.6702$. (**O**) Representative immunohistochemical staining images of Ucp1 in BAT sections; scale bar = 50 µm. (**P**) Representative immunohistochemical staining images of Ucp1 in eWAT sections; scale bar = 50 µm. (**Q**) Representative immunoblot images of Ucp1 in mature primary brown adipocytes treated with varying concentrations of ISM. (**S**) Quantitative analysis of relative protein expression levels of Ucp1 in mature primary brown adipocytes treated with varying concentrations of ISM ($n = 3$). 0 vs. 2, $p = 0.6256$; 0 vs. 10, $p = 0.8106$; 0 vs. 50, $p = 0.3200$. *n* presents biological replicates. Data are presented as mean ± SEM. Statistical test: (**S**): one-way ANOVA followed by Dunnett's multiple comparisons test. The others: unpaired Student's two-tailed t-tests. Statistical significance is indicated as follows: *$p < 0.05$, **$p < 0.01$, ***$p < 0.001$, ****$p < 0.0001$, and ns indicates no significant difference.

 © The Author(s)

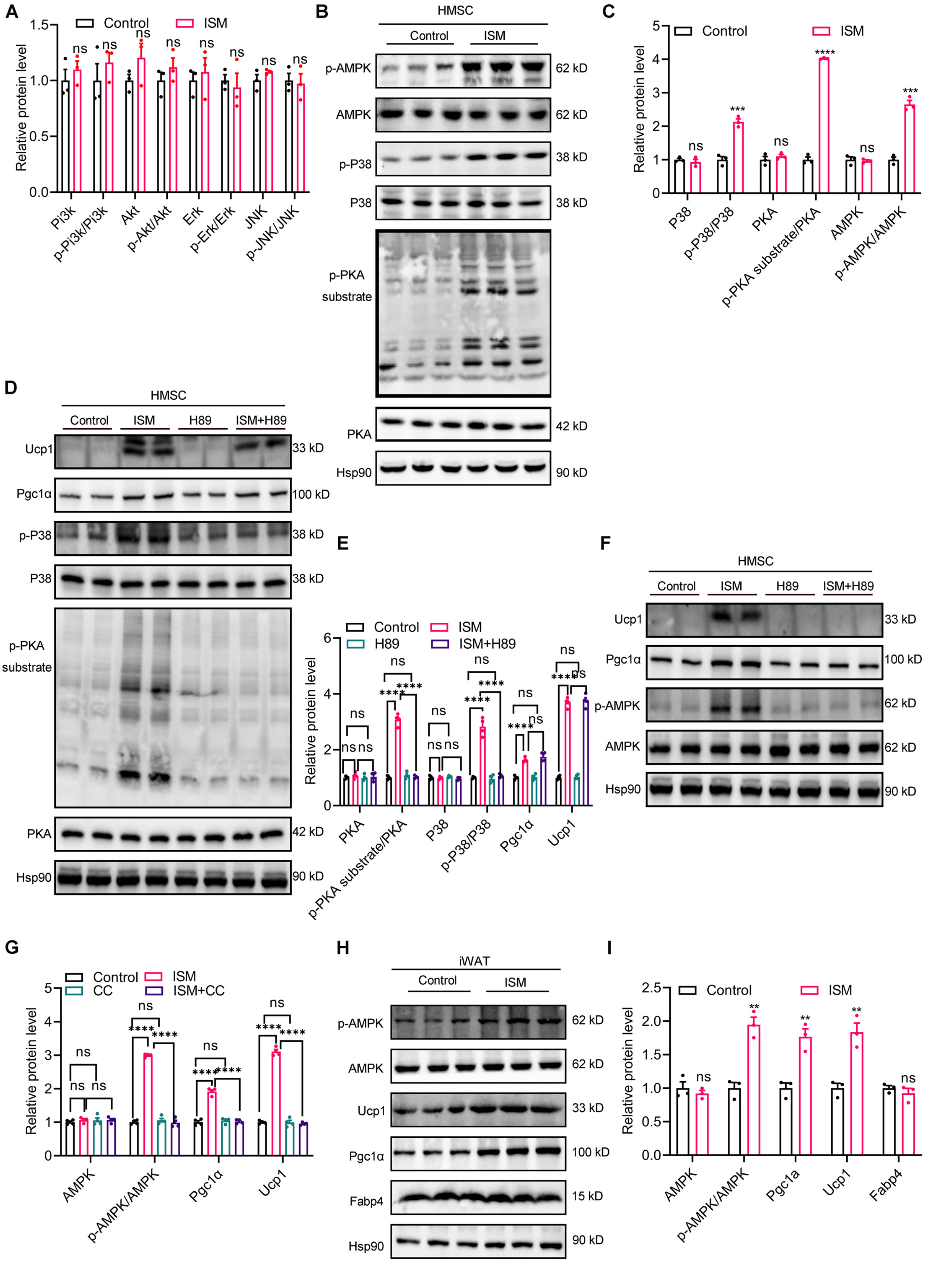
A
Control
ISM
Relative protein level
1.5
1.0
0.5
0.0
ns
Pi3k
p-Pi3k/Pi3k
Akt
p-Akt/Akt
Erk
p-Erk/Erk
JNK
p-JNK/JNK
B
HMSC
Control
ISM
p-AMPK
62 kD
AMPK
62 kD
p-P38
38 kD
P38
38 kD
p-PKA substrate
PKA
42 kD
Hsp90
90 kD
C
Control
ISM
Relative protein level
5
4
3
2
1
0
ns
***
****
P38
p-P38/P38
PKA
p-PKA substrate/PKA
AMPK
p-AMPK/AMPK
D
HMSC
Control
ISM
H89
ISM+H89
Ucp1
33 kD
Pgc1α
100 kD
p-P38
38 kD
P38
38 kD
p-PKA substrate
PKA
42 kD
Hsp90
90 kD
E
Control
ISM
H89
ISM+H89
Relative protein level
6
4
2
0
ns
****
PKA
p-PKA substrate/PKA
P38
p-P38/P38
Pgc1α
Ucp1
F
HMSC
Control
ISM
H89
ISM+H89
Ucp1
33 kD
Pgc1α
100 kD
p-AMPK
62 kD
AMPK
62 kD
Hsp90
90 kD
G
Control
ISM
CC
ISM+CC
Relative protein level
5
4
3
2
1
0
ns
****
AMPK
p-AMPK/AMPK
Pgc1α
Ucp1
H
iWAT
Control
ISM
p-AMPK
62 kD
AMPK
62 kD
Ucp1
33 kD
Pgc1α
100 kD
Fabp4
15 kD
Hsp90
90 kD
I
Control
ISM
Relative protein level
2.5
2.0
1.5
1.0
0.5
0.0
ns
**
AMPK
p-AMPK/AMPK
Pgc1a
Ucp1
Fabp4

© The Author(s)

**Figure EV6. ISM promotes browning by activating the AMPK pathway.**

(**A**) Quantitative analysis of relative protein expression levels for the signaling pathway proteins shown in Fig. 5C ($n = 3$). Unpaired Student's two-tailed t-tests. Pi3k, $p = 0.4749$; p-Pi3k/Pi3k, $p = 0.4071$; Akt, $p = 0.2135$; p-Akt/Akt, $p = 0.3419$; Erk, $p = 0.6300$; p-Erk/Erk, $p = 0.6766$; JNK, $p = 0.2356$; p-JNK/JNK, $p = 0.8187$. (**B**) Representative immunoblot images of signaling pathway proteins in mature human adipocytes from control and ISM (50 μM) treatment groups. (**C**) Quantitative analysis of relative protein expression levels for the signaling pathway proteins shown in (**B**) ($n = 3$). Unpaired Student's two-tailed t-tests. P38, $p = 0.4837$; p-P38/P38, $p = 0.0010$; PKA, $p = 0.4066$; p-PKA substrate/PKA, $p < 0.0001$; AMPK, $p = 0.6970$; p-AMPK/AMPK, $p = 0.0003$. (**D**) Representative immunoblot images of signaling pathway proteins in mature human adipocytes treated with or without PKA inhibitor H89 (30 μM) for 2 h, followed by ISM or DMSO treatment for 48 h. (**E**) Quantitative analysis of relative protein expression levels for the signaling pathway proteins shown in (**D**) ($n = 3$). One-way ANOVA followed by Tukey's multiple comparisons. PKA: Control vs. ISM, $p = 0.3360$; Control vs. H89, $p = 0.9175$; ISM vs. ISM + H89, $p = 0.7420$; p-PKA substrate/PKA: Control vs. ISM, $p < 0.0001$; Control vs. H89, $p = 0.2637$; ISM vs. ISM + H89, $p < 0.0001$; P38: Control vs. ISM, $p = 0.7647$; Control vs. H89, $p = 0.4293$; ISM vs. ISM + H89, $p = 0.4467$; p-P38/P38: Control vs. ISM, $p < 0.0001$; Control vs. H89, $p = 0.5466$; ISM vs. ISM + H89, $p < 0.0001$; Pgc1α: Control vs. ISM, $p < 0.0001$; Control vs. H89, $p = 0.8756$; ISM vs. ISM + H89, $p = 0.2669$; Ucp1: Control vs. ISM, $p < 0.0001$; Control vs. H89, $p = 0.5887$; ISM vs. ISM + H89, $p = 0.6622$. (**F**) Representative immunoblot images of signaling pathway proteins in mature human adipocytes treated with or without AMPK inhibitor CC (10 μM) for 2 h, followed by ISM or DMSO treatment for 48 h. (**G**) Quantitative analysis of relative protein expression levels for the signaling pathway proteins shown in (**F**) ($n = 3$). One-way ANOVA followed by Tukey's multiple comparisons. AMPK: Control vs. ISM, $p = 0.2372$; Control vs. CC, $p = 0.5120$; ISM vs. ISM + CC, $p = 0.9842$; p-AMPK/AMPK: Control vs. ISM, $p < 0.0001$; Control vs. CC, $p = 0.3807$; ISM vs. ISM + CC, $p < 0.0001$; Pgc1α: Control vs. ISM, $p < 0.0001$; Control vs. CC, $p = 0.4606$; ISM vs. ISM + CC, $p < 0.0001$; Ucp1: Control vs. ISM, $p < 0.0001$; Control vs. CC, $p = 0.9252$; ISM vs. ISM + CC, $p < 0.0001$. (**H**) Representative immunoblot images of AMPK, p-AMPK, Ucp1, Pgc1α, and Fabp4 in iWAT from mice fed a normal diet and injected with ISM or vehicle. (**I**) Quantitative analysis of relative protein expression levels for the proteins shown in (**H**) ($n = 3$). Unpaired Student's two-tailed t-tests. AMPK, $p = 0.4924$; p-AMPK/AMPK, $p = 0.0026$; Pgc1α, $p = 0.0055$; Ucp1, $p = 0.0056$; Fabp4, $p = 0.4072$. *n* presents biological replicates. Data are presented as mean ± SEM. Statistical significance is indicated as follows: *$p < 0.05$, **$p < 0.01$, ***$p < 0.001$, ****$p < 0.0001$, and ns indicates no significant difference.


© The Author(s)

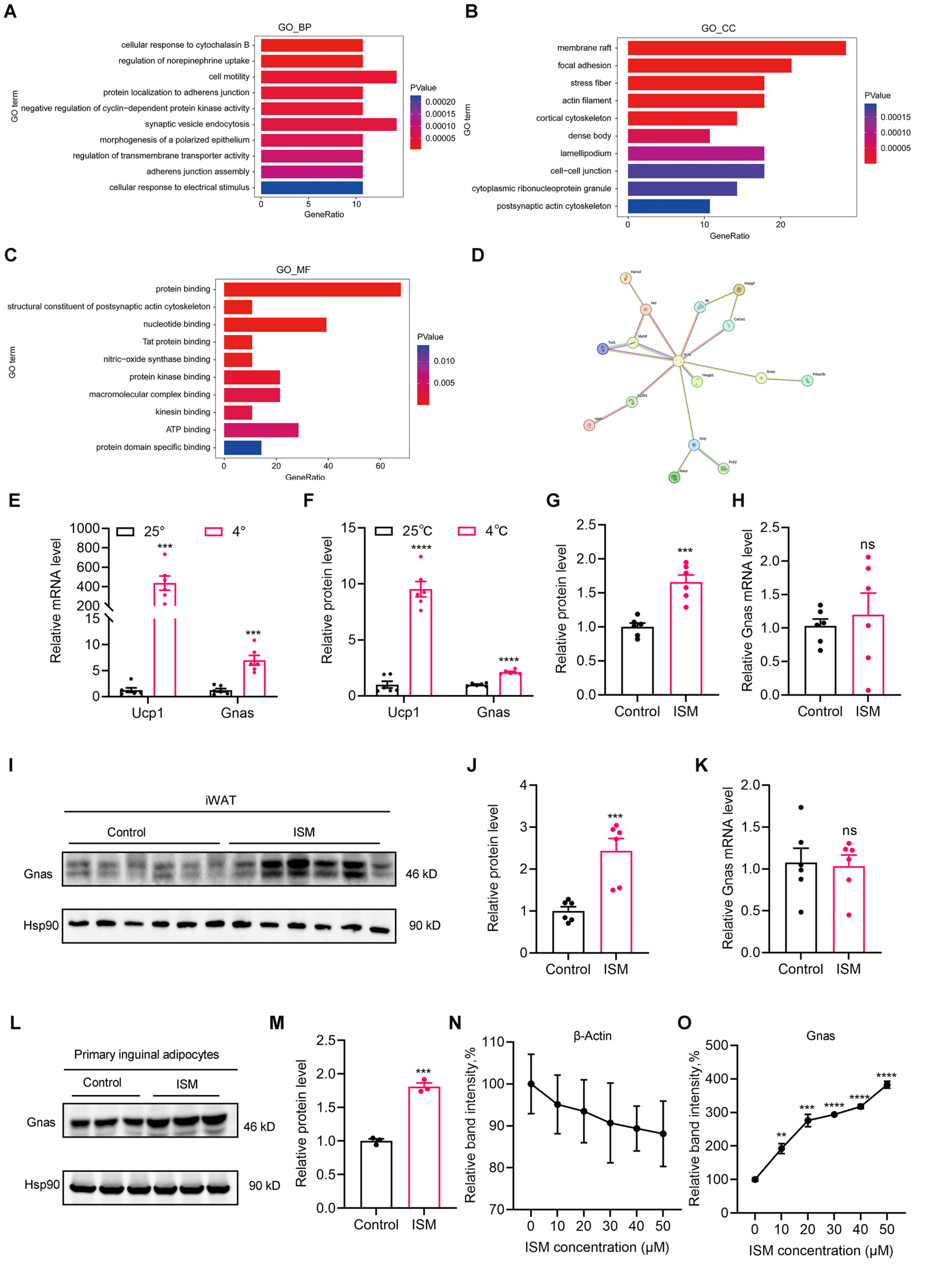
A
GO_BP
cellular response to cytochalasin B
regulation of norepinephrine uptake
cell motility
protein localization to adherens junction
negative regulation of cyclin−dependent protein kinase activity
synaptic vesicle endocytosis
morphogenesis of a polarized epithelium
regulation of transmembrane transporter activity
adherens junction assembly
cellular response to electrical stimulus
GO term
GeneRatio
PValue
B
GO_CC
membrane raft
focal adhesion
stress fiber
actin filament
cortical cytoskeleton
dense body
lamellipodium
cell−cell junction
cytoplasmic ribonucleoprotein granule
postsynaptic actin cytoskeleton
C
GO_MF
protein binding
structural constituent of postsynaptic actin cytoskeleton
nucleotide binding
Tat protein binding
nitric−oxide synthase binding
protein kinase binding
macromolecular complex binding
kinesin binding
ATP binding
protein domain specific binding
D
E
25° 4°
Relative mRNA level
Ucp1 Gnas
F
25℃ 4℃
Relative protein level
Ucp1 Gnas
G
Relative protein level
Control ISM
H
Relative Gnas mRNA level
ns
Control ISM
I
iWAT
Control ISM
Gnas 46 kD
Hsp90 90 kD
J
Relative protein level
Control ISM
K
Relative Gnas mRNA level
ns
Control ISM
L
Primary inguinal adipocytes
Control ISM
Gnas 46 kD
Hsp90 90 kD
M
Relative protein level
Control ISM
N
β-Actin
Relative band intensity,%
ISM concentration (μM)
O
Gnas
Relative band intensity,%
ISM concentration (μM)

**Figure EV7. Discovery of direct binding targets of ISM.**

(**A–C**) Bar charts of GO enrichment analysis for 26 potential target proteins. (**D**) Protein–protein interaction (PPI) network diagram of 26 potential target proteins. (**E**) Relative mRNA expression levels of Gnas and Ucp1 in iWAT of 8-week-old male C57BL/6J mice exposed to 25 °C or 4 °C environments for 3 days ($n = 6$). Ucp1, $p = 0.0001$; Gnas, $p = 0.0002$. (**F**) Quantitative analysis of relative expression levels for the proteins shown in Fig. 6D ($n = 6$). Ucp1, $p < 0.0001$; Gnas, $p < 0.0001$. (**G**) Quantitative analysis of relative protein levels for the Gnas shown in Fig. 6E ($n = 6$). $p = 0.0003$. (**H**) Relative mRNA levels of Gnas in iWAT of mice fed a high-fat diet and injected with ISM or vehicle ($n = 6$). $p = 0.6227$. (**I**) Representative immunoblot images of Gnas in iWAT of mice fed a normal diet and injected with ISM or vehicle. (**J**) Quantitative analysis of relative protein levels for the Gnas shown in (**I**) ($n = 6$). $p = 0.0009$. (**K**) Relative mRNA levels of Gnas in iWAT of mice fed a normal diet and injected with ISM or vehicle ($n = 6$). $p = 0.8522$. (**L**) Representative immunoblot images of Gnas in mature primary inguinal adipocytes from control and ISM (50 μM) treatment groups. (**M**) Quantitative analysis of relative protein levels for the Gnas shown in (**L**) ($n = 3$). $p = 0.0001$. (**N**) Quantitative analysis of relative grayscale values for β-actin in Fig. 6J ($n = 3$). 0 vs. 10, $p = 0.9860$; 0 vs. 20, $p = 0.9547$; 0 vs. 30, $p = 0.8439$; 0 vs. 40, $p = 0.7713$; 0 vs. 50, $p = 0.6964$. (**O**) Quantitative analysis of relative grayscale values for Gnas in Fig. 6J ($n = 3$). 0 vs. 10, $p = 0.0003$;0 vs. 20, $p < 0.0001$; 0 vs. 30, $p < 0.0001$; 0 vs. 40, $p < 0.0001$; 0 vs. 50, $p < 0.0001$. $n$ presents biological replicates. Data are presented as mean ± SEM. Statistical test: (**N** and **O**): one-way ANOVA followed by Dunnett's multiple comparisons test. The others: unpaired Student's two-tailed t-tests. Statistical significance is indicated as follows: *$p < 0.05$, **$p < 0.01$, ***$p < 0.001$, ****$p < 0.0001$, and ns indicates no significant difference.

 © The Author(s)
